# Ant parasitoidism in checkered beetles: *Phyllobaenus obscurus* developing inside intact cocoons of two species of the *Ectatomma ruidum* species complex

**DOI:** 10.1371/journal.pone.0335300

**Published:** 2026-03-20

**Authors:** Gabriela Pérez-Lachaud, Chantal Poteaux, John M. Leavengood Jr, Jean-Paul Lachaud

**Affiliations:** 1 Dpto. Conservación de la Biodiversidad, El Colegio de la Frontera Sur, Chetumal, Quintana Roo, Mexico; 2 Laboratoire d’Éthologie Expérimentale et Comparée UR 4443 (LEEC), Université Sorbonne Paris Nord, Villetaneuse, France; 3 United States Department of Agriculture, APHIS, PPQ, Tampa, Florida, United States of America; Charles University: Univerzita Karlova, CZECHIA

## Abstract

Known parasitoids of ants include species from several families of flies, wasps, strepsipterans, nematodes, and mites. Curiously, while myrmecophily is heavily biased towards Coleoptera, one of the most diverse and speciose insect orders, no beetles specialized as parasitoids of ants have been recorded, although the parasitoid habit has evolved at least 13 times within this order. Here we report on observations that strongly suggest that a checkered beetle species behaves as a parasitoid of ant brood. A total of 146 colonies or part of colonies of three species of the *Ectatomma ruidum* species complex (*E. ruidum* sp. 2, 3, 4) were excavated in several sites along the Pacific coastal plains of Oaxaca, Mexico, during three collecting campaigns (2015–2017). Overall, 11060 adults, 5795 cocoons and 2185 larvae were examined. Upon dissection, four intact, ethanol-preserved cocoons contained ant prepupae/pupae parasitized by characteristic campodeiform beetle larvae (prognathous head, three pairs of segmented legs, no prolegs, body with sparse but long pubescence), and a fifth cocoon presented a round exit hole. Sequencing of the COI gene fragment support assignment of the larva to Cleridae, consistent with the genus *Phyllobaenus* (Cleridae). An active, pink-colored larva that emerged from a cocoon containing the remains of the ant host pupa, was reared to adulthood and could be identified as *Phyllobaenus obscurus* (Gorham). Beetle larvae were found inside intact cocoons of two species: *E. ruidum* sp. 3 and sp. 4. The prevalence of parasitism is extremely low, representing less than 0.6% of available cocoons. Predatory during both adult and larval stages, checkered beetles are broadly known as predators of wood-boring and cone-boring beetles, and some species are facultative parasitoids of solitary bees or wasps or, very rarely, specialized in predating social insects. We assert that the novel discovery of clerid-ant brood parasitoidism within the subterranean host colony deviates yet further from any adaptation known to date among clerids.

## Introduction

Many invertebrates have adopted a predatory or parasitoid life strategy and play an important role in biological control [1–3]. While predators capture and consume multiple prey during their developmental and/or adult life stage, a parasitoid is an organism in which the immature stages develop on or within a single organism (the host), with this feeding activity eventually resulting in the death or sterilization of the host [4,5], whereas adult parasitoid organisms are free-living. A diverse array of parasitoids specializes in attacking adult ants or their brood. Known ant parasitoids include species of several insect orders and families: flies, twisted-wing parasites, and parasitic wasps [6–11], two families of Nematoda [12], and one mite family [13–15], but no coleopteran species has been recorded as parasitoid of ants yet. Ant parasitoids are highly specialized symbionts that display a variety of morphological, chemical, and behavioral strategies for accessing and successfully parasitizing their social hosts [13,16,17]. For example, species in the genera *Elasmosoma* Ruthe and *Neoneurus* Haliday (Hymenoptera, Braconidae) that parasitize adult ants outside the nest have morphological adaptations for grasping workers and securing oviposition (e.g., tarsal modifications, raptorial legs) and rely on rapidity to oviposit directly into the ant body [18]. Wasps in the Eucharitidae family lay eggs away from their hosts, in or on plant substrate; they possess a very mobile first-instar larva, termed planidium*,* which is responsible for accessing the hosts through phoresis on foraging ants or attached to prey [19–23]*.* Chemical volatiles of glandular origin used by ants as pheromones are co-opted by ant-decapitating flies (Phoridae) to locate potential hosts [24]. Once in the vicinity, phorid females rely on short-range chemical signals, such as cuticular hydrocarbons, to locate and select potential hosts [25].

According to Eggleton and Belshaw [4,26], the parasitoid habit has evolved independently at least 13 times in Coleoptera. Beetles do not have piercing ovipositors and the poor flying ability of the adult contrasts with the mobility of the larval stages [4,26]. As in the case of eucharitid wasps and Strepsiptera, known species of Coleoptera with a parasitoid lifestyle lay eggs away from the hosts and possess phoretic first-instar larvae, termed triungulin, that are responsible for locating and selecting suitable hosts (Meloidae, Ripiphoridae, some Cleridae [4,19]). The phoretic larvae attach to the adults of their hosts either on living plants, dead wood or soil. Most parasitoid coleopteran families appear to have evolved from ancestors associated with dead wood, with some shifting to attacking insects in the soil. Hosts of coleopteran parasitoids include Diplopoda, Thysanura, Blattaria, Thysanoptera, other Coleoptera, Hymenoptera (other than Formicidae), and Lepidoptera [4]. Compared with parasitoid wasps and flies, their host taxonomic diversity is rather low, suggesting that other factors may constrain the host range of coleopteran parasitoids [4]. Contradictorily, evolution of myrmecophily in insects is heavily biased towards the Coleoptera. The nature of colony exploitation by myrmecophilous beetles varies significantly, with species ranging from scavengers and refuse dwellers to tolerated or highly integrated guests [27]. Some adult myrmecophilous beetles, as in the genus *Cremastocheilus* Knoch [28] (Scarabaeidae) or in various Paussinae species [29] (Carabidae), are predatory on ant brood and exhibit various strategies to locate and exploit their prey within the ant host nest. Some species, such as *Myrmedonota xipe* Mathis & Eldredge (Staphylinidae), can even use specialized parasitoid host location cues to selectively prey on *Azteca sericeasur* Longino workers that have been previously parasitized by phorid flies [30]. However, to the best of our knowledge, no species of beetle has ever been recorded as developing as a parasitoid of adult ants or their brood. Here we report on observations that strongly suggest that a species of Cleridae develops as a parasitoid of ant brood.

Cleridae is a family of about 4710 described species (R. Gerstmeier, personal communication). Predatory during both adult and larval stages, these beetles are broadly known as predators of wood-boring and cone-boring beetles (e.g., bark beetles, longhorn beetles, powderpost beetles) and are often observed in the galleries of their prey [19,31]. However, several clerid genera are known to feed on gall-makers and their parasitoids (e.g., *Cymatodera* Gray, *Neohydnocera* Leavengood, *Phyllobaenus* Dejean, *Placopterus* Wolcott), on pollen as a primary or secondary protein source (some Clerinae, Korynetinae, Epiclininae), on grasshopper egg pods (*Trichodes* Herbst, *Aulicus* Spinola), on non-gallmaking caterpillars (e.g., *Aulicus, Phyllobaenus*), on insects associated with carrion and stored food products and their pests (*Necrobia* Latreille), or have been reared from cells of bee and wasp larvae (e.g., *Cymatodera, Lecontella* Wolcott & Chapin, *Phyllobaenus, Placopterus*, *Trichodes*) [31,32]. Many species exhibit several different larval feeding strategies, which all may appear to be specialized behaviors (see Table 1). For example, the larvae of *Placopterus thoracicus* (Olivier), which is strongly associated with wood-boring beetles, have been found inside insect galls, and the adults have been reared from cells of crabronid and pemphredonid wasps [48]. Similarly, in *Neohydnocera longicollis* (Ziegler), formerly referred to as *Hydnocera longicollis* Ziegler and *Isohydnocera curtipennis* (Newman), adults have been reported emerging from the galls of three different hosts in three different insect orders (Diptera, Hymenoptera and Lepidoptera) [38,45,56,57]. On the other hand, various species, mainly from the genus *Trichodes*, develop as cleptoparasitoids (development at the expense of a single host organism by at least partial usurpation of its food supply, resulting in the death of the host [4]), and many other species have been reared from galls or nest cells of Hymenoptera while also exhibiting other predatory behaviors [31,32,68] (see Table 1). In general, however, the natural histories of most species in these genera are poorly documented.

**Table 1:** Summary of known rearing records among North American Cleridae and larval feeding mode on insects other than wood, stem and twig borers.

Clerid subfamily and tribe	Species	From galls induced by insects	From nests/cells of solitary bees or wasps	From nests/cells of social Hymenoptera	Other insect prey	Larval feeding mode	References
Tillinae: no tribal assignment	*Cymatodera* sp.		Megachilidae Vespidae			Pr	[33,34]
	*Cymatodera ovipennis* LeConte		Apidae Megachilidae			Pr	[35]
	*Cymatodera undulata* (Say)	Cynipidae^Hy^	Sphecidae		Gall inquilines Chalcididae^Hy^ Cynipidae^H^ Eurytomidae^Hy^ Pteromalidae^Hy^	Pr	[36–39]
	*Lecontella brunnea* (Spinola)		Unident. bees Megachilidae Sphecidae Vespidae			Pr, Pa?	[40–42]
Clerinae: Clerini	*Aulicus terrestris* Linsley				Acrididae^Or^	Pr	[43]
	*Enoclerus rosmarus* (Say)	Unident. insect Tephritidae^Di^			Gall inquilines	Pr	[44,45]
	*Enoclerus* sp.				Tortricidae^Le^	Pr	[46]
	*Enoclerus zonatus* (Klug)				*Blastobasidae^Le^ *Nitidulidae^Le^ *Prodoxidae^Le^	Pr	[47]
	*Placopterus thoracicus* (Olivier)	Cynipidae^Hy^	Crabronidae Pemphredonidae		Gall inquilines	Pr	[37,48]
Clerinae: Dieropsini	*Trichodes apivorus* Germar**		Unident. bees Unident. wasps			Pr	[49,50]
	*Trichodes bibalteatus* LeConte		Megachilidae			Pr	[51]
	*Trichodes bicinctus* Green		Apidae			Pr	[51]
	*Trichodes nuttalli* (Kirby)**		Unident. bees Unident. wasps		Acrididae^Or^	Pr	[50,51]
	*Trichodes oregonensis* Barr				Acrididae^Or^		[51]
	*Trichodes oresterus* Wolcott		Megachilidae				[51]
	*Trichodes ornatus bonnevillensis* Foster		Megachilidae Vespidae			Pr	[33,34,51]
	*Trichodes ornatus douglasianus* White		Apidae Megachilidae Vespidae			Pr, Pa?	[51–54]
	*Trichodes ornatus hartwegianus* White		Megachilidae			Pr	[51]
	*Trichodes ornatus ornatus* Say		Unident. bees Unident. wasps Megachilidae		Post-copulatory cannibalism	Pr	[50,51,54]
	*Trichodes ornatus tenuosus* Foster		Megachilidae Sphecidae				[34,51,55]
	*Trichodes peninsularis horni* Wolcott & Chapin		Megachilidae			Pr	[39,51]
	*Trichodes simulator* Horn		Apidae Megachilidae			Pr, Pa?	[51]
Clerinae: Hydnocerini	*Neohydnocera longicollis* (Ziegler)	Tephritidae^Di^ Tenthredinidae^Hy^Gelechiidae^Le^			Gall inquilines	Pr, Pa?	[38,45,56,57]
	*Neohydnocera tabida* (LeConte)	Tephritidae^Di^			Gall inquilines	Pr	[58]
	*Phyllobaenus atriplexus* Foster	Cecidomyiidae^Di^			Gall inquilines Chalcididae^Hy^ Eulophidae^Hy^ Eupelmidae^Hy^ Eurytomidae^Hy^ Platygastridae^Hy^ Pteromalidae^Hy^ Torymidae^Hy^	Pr	[59–61]
	*Phyllobaenus caeruleipennis* (Wolcott)	Tephritidae^Di^			Gall inquilines	Pr	[62]
	*Phyllobaenus dubius* (Wolcott)				Cephidae^Hy^	Pr	[63]
	*Phyllobaenus humeralis* (Say)	Tephritidae^Di^			Gall inquilines	Pr	[45]
	*Phyllobaenus knausii* (Wickham)	Unident. insect			Gall inquilines	Pr	[57]
	*Phyllobaenus maritimus* Wolcott				Tortricidae^Le^	Pr	[46]
	***Phyllobaenus obscurus* (Gorham)**			**Formicidae** ^**Hy**^		**Pa**	**This study**
	*Phyllobaenus pubescens* (LeConte)	Unident. insect	Megachilidae		Gall inquilines Braconidae^Hy^ Noctuidae^Le^	Pr	[44,57,64–66]
	*Phyllobaenus scaber* (LeConte)	Gelechiidae^Le^			Gall inquilines Aphididae^He^ Braconidae^Hy^ Eurytomidae^Hy^ Tortricidae^Le^ Psocidae^Ps^ Unident. spiders	Pr	[67,68]
	*Phyllobaenus unifasciatus* (Say)	Cynipidae^Hy^ Unident. insect			Gall inquilines	Pr	[37,57]
	*Phyllobaenus subaeneus* (Spinola)				Tortricidae^Le^	Pr	[46]
	*Phyllobaenus unifasciatus* (Say)	Cynipidae^Hy^ Unident. insect			Gall inquilines	Pr	[37,57]
	*Phyllobaenus verticalis* (Say)	Cynipidae^Hy^			Gall inquilines	Pr	[37,38,69]

*: Putative prey attacked by larvae.

**: Questionable information. According to Foster [51], the references that identify *T. apivorus* as a predator in bee and wasp nests “*were inferred apparently from the specific name or from what was known of* Trichodes *biology in general*”. However, there are no reliable reports of bees or wasps serving as larval hosts for *T. apivorus* and the same seems true for *T. nuttalli* for which the only known larval host is the acridid Orthoptera, *Chloealtis conspersa* (Harris).

Di: Diptera; He: Hemiptera; Hy: Hymenoptera; Le: Lepidoptera; Or: Orthoptera; Pa: Parasitoid; Pa?: Facutative parasitoid; Pr: Predator; Ps: Psocoptera; Unident: unidentified.

In Europe, the association between *Trichodes umbellatarum* Olivier and the larvae of various bee species borders parasitoidism and predation [70,71], some larvae developing upon a single host as facultative parasitoids while others need several hosts to complete development, acting as predators. Similar shifts between predation and facultative parasitoidism have also been reported in the New World for some species such as *Lecontella brunnea* (Spinola), *Trichodes ornatus douglasianus* White, *T. simulator* Horn, and *N. longicollis* [42,51,52,57] (Table 1). However, no clerid species associated with hymenopterans can compare to the unique case of *Tarsobaenus letourneauae* Leavengood, Pinkerton & Rifkind and *T. piper* Leavengood, Pinkerton & Rifkind which exhibit a remarkable symbiotic relationship with ants and their host plants [72]. Larvae of both *Tarsobaenus* species have been reported inhabiting the petiole chambers of the Piperaceae *Piper cenocladum* Casimir de Candolle, and *P. obliquum* Ruiz & Pavón, respectively, which host the myrmicine ant *Pheidole bicornis* Forel, and feeding either on the ant brood when ants are present or on the plant-produced food bodies when ants are absent; remarkably, translocation experiments showed that, in the absence of ants, the clerid larva induced food-body production too [73]. Although the evolution of parasitism of this ant-plant mutualism is intriguing, we assert that the novel discovery of clerid-ant brood parasitism within *Ectatomma* colonies is without precedent among all previously known adaptations in clerids. Here we report on the first recorded instance of a checkered beetle developing as a parasitoid of the ant brood which also represents the first case of a primary parasitoid of ants from the order Coleoptera. We also summarize all known rearing records among North American Cleridae and discuss the evolutionary transitions and diversification of larval feeding habits within Cleridae.

## Materials and methods

### The ant hosts

The neotropical ant *Ectatomma ruidum* (Roger) has historically been considered as a widely distributed ant taxon throughout the Neotropics, from Mexico to Brazil [74–77], readily visually recognized. These ants have been subject of intense investigation into colony organization, communication, etc., serving as a species model system for several studies including research on intraspecific cleptobiosis, a very rare behavioral trait [78–80]. Recent studies have revealed that this taxon represents indeed a species complex [77,81–84] including at least 6 putative species, which can be separated based on molecular, chemical and acoustic signatures, and on behavioral traits, but with a considerably conserved morphology which has prevented species description. Two species have a wide distribution, from Mexico to northern Argentina (*E. ruidum* sp. 1 and *E. ruidum* sp. 2), and four other species are apparently restricted to few localities in southern Mexico, along the coast of Oaxaca [83,84] where this study was performed (see below). The most abundant and accessible species in the study area was *E. ruidum* sp. 4, which was observed in at least three sites in the municipality of Santa María Tonameca. The species *E. ruidum* sp. 2 (although the most common in Mexico) was only found in a low coastal forest area in the Municipality of Santa María Huatulco, and the distribution of *E. ruidum* sp. 3 was extremely limited, as it is known to occur at only one site (Rancheria Yerba Santa, in the Municipality of San Pedro Mixtepec) that is no longer accessible because it has become private property. For more details on collection sites, see [83,84].

These ants occur in a wide range of habitats in the Neotropics, including plantations and conserved forests, from sea level up to 1600 m [85,86]. Colonies of these species are monodomous with one single exception [87]. Typically, *E. ruidum* nests have a single entrance about 3–4 mm wide dug in the ground [88,89]. In Chiapas, southern Mexico, colonies are made up of 50–200 individuals [90] and colony density can be very high locally (up to 11,200 nests per hectare [91]). These ants have a very generalist diet, and their foraging activity is mainly diurnal [89]. In general, colonies are monogynous, but in some regions they are facultatively polygynous with size-dimorphic queens (macro and microgynes) [92,93].

### Study site, ant sampling, and parasitoid identification

Beetle larvae were collected along with the ants as part of a larger project aimed at characterizing/unveiling cryptic species in the *E. ruidum* complex and assessing the diversity of some of their already recorded parasitoids (parasitic Hymenoptera, Diptera, and Nematodes, see [94]). Collecting an equal number of colonies from the different cryptic species of this ant complex was not a prerequisite for this program, and therefore no effort was made in this regard. Furthermore, the genetic identification of the species could only be carried out retrospectively. Colonies or part of colonies were collected in several sites during three collecting campaigns (November 2015, October 2016 and October/November 2017) in the Pacific Coastal Plain of Oaxaca, Mexico (Table 2). Sampling was carried out in relatively undisturbed areas (very low urbanization and non-intensive farming) and sites were selected based on the presence of the target ants and their accessibility (both physically and in terms of landowner permission, see “Collection permits” below). Climate is typical of the Pacific watershed lowlands in southern Mexico, i.e., warm sub-humid with precipitation concentrated in the summer months (Aw0 [95]); mean annual temperature is 22–28 °C; mean annual precipitation is ca. 900 mm. The dominant plant communities in the lowlands (50–450 m asl) are classified as seasonally dry tropical forest [96].

**Table 2:** Summary of the collection sites in the Oaxaca coast (Mexico) and number of colonies of the different *Ectatomma ruidum* species sampled during 2015-2017.

*Ectatomma ruidum* species *sensu* Meza-Lázaro et al. [82]	Site	GPS coordinates	Altitud (m asl)	Nb. of sampled colonies (colonies with cocoons)		
				2015	2016	2017
*E. ruidum* sp. 2	Bajos de Coyula	15°43′58.8“ N, 96°17′24” W	125	5 (4)	28 (14)	–
*E. ruidum* sp. 3	Yerba Santa	15°56′28“N, 97°04′27” W	156	4 (4)	24 (18)	–
*E. ruidum* sp. 4	Piedras Negras	15°43′49.7“N, 96°40′21.7” W	18	–	22 (13)	–
*E. ruidum* sp. 4	El Zapotal (Mazunte)	15°41′7.4“ N, 96°33′42.5” W	42	4 (4)	22(9)	15 (15)
*E. ruidum* sp. 4	Puente Cuatode	15°43’55.2” N, 96°30’32.4” W	89	–	22 (17)	–
				13 (12)	118 (71)	15 (15)

In 2015, three people participated in nest excavations, and 13 colonies from three species (*E. ruidum* sp.1, sp. 2, and sp. 3) were collected from three sites, 12 of them with cocoons. In 2016, six people were available and we were able to sample more sites (five) and excavate more nests (118), with a total of 71 complete colonies collected from the same three species. In 2017, only two people were available. Furthermore, access to certain collection sites was either impossible due to the insecurity prevailing in the region at that time or denied by the owners of the land concerned due to socio-political and socio-economic tensions in the study area, reducing the number of collection sites to just one, where only 15 complete colonies of *E. ruidum* sp. 4 could be excavated.

At each site, the entrances of ant nests were detected using cookie crumbs as bait and following foraging ants back to their nest. Colonies were excavated and all the individuals present in the nest chambers were collected in plastic bags and transported to the field lab for initial revision. In 2015 and 2017, cocoons of each colony were kept in Petri dishes for a couple of weeks to wait for parasitoid emergence and later inspected and dissected under a stereomicroscope. During the 2016 campaign, when sampling efforts were intensified, cocoons of each colony were separately preserved in 96° alcohol and revised and counted later under a stereomicroscope, as well as the rest of material. Ant larvae were thoroughly examined for the presence of any planidium or evidence of parasitoid attack (presence of scars evidencing a previous unsuccessful attack or signs of endoparasite presence, see [9,11]). All the cocoons collected were externally revised one by one and then were carefully dissected and their contents examined. The identity, number and developmental stage of any parasitoid were recorded. Adult ants were also closely examined for the presence of potential ecto- or endoparasites (e.g., phorid flies, strepsipterans, mites, nematodes, or planidia) attached to their body.

Eucharitid wasps were identified using available keys [97]. Beetles were morphologically identified by one of us (JML). Beetle larvae and the adult reared from cocoons (see Results section) were initially assigned to the same species based on morphology and context. Later, the sequence of the standard barcoding fragment (cytochrome oxidase I, COI) of one larva was obtained. Due to the reduced number of specimens and poor state of the adult, only one larva was extracted. DNA extraction, amplification, and sequencing followed standard protocols [98], using the zooplankton primers developed by Prosser et al. [99]. The COI fragment was sequenced bidirectionally; the sequences were edited using CodonCode v. 3.0.1 (CodonCode Corporation, Dedham, MA, USA). The quality of the forward and reverse reads was assessed using FASTQC [100], with sequence sites evaluated using a minimum quality score threshold of 40. Translation of the 657-nucleotide sequence into a protein sequence produced a chain of 219 amino acids, with no internal stop codons detected. The sequence, along with its corresponding amino acid sequence, was deposited in the Barcode of Life Database (BOLD, boldsystems.org, BOLD:AHE8421) and in GenBank (accession number PX893661). The genetic distances were computed using MEGA 7.0 [101] with the Kimura-2-parameter model.

Voucher specimens of ants (*E. ruidum* sp. 4: five workers, catalog # F-02128 to F-02132; *E. ruidum* sp. 3: five workers, catalog # F-02123 to F-02127, and three workers in the same sample vial, catalog # F-03222), the adult beetle and the extracted larva (catalog # AR-0742) were deposited in the Formicidae and Arthropoda collections of El Colegio de la Frontera Sur-Chetumal (ECO-CH-F and ECO-CH-AR, respectively).

### Collection permits

Field sampling complied with the current laws of Mexico (collection permit FAUT-0277 and FAUT-0208 from Secretaría del Medio Ambiente y Recursos Naturales – Dirección General de Vida Silvestre granted to GP-L and to Alejandro Zaldívar-Riverón (UNAM, Mexico) who helped during colony sampling, respectively). No other governmental permit was required to access the study sites, but formal permission was granted by the owners of the fields where ant nests were excavated.

## Results

### Colony composition and within-nest associated organisms

A total of 146 colonies or part of colonies of three species of the *Ectatomma ruidum* complex (*E. ruidum* sp. 2, sp. 3, and sp. 4 *sensu* Meza-Lázaro et al. [82]) were sampled. Cocoons were present in a total of 98 excavated colonies or part of colonies (18 colonies for *E. ruidum* sp. 2, 22 for *E. ruidum* sp. 3, and 58 for *E. ruidum* sp. 4), and larvae were found in 79 of these colonies (Tables 2, 3, S1). Overall, 11060 adults, 5795 cocoons and 2185 larvae were examined.

**Table 3:** Infestation rate of *Ectatomma ruidum* spp. cocoons by *Phyllobaenus obscurus.*

Year of collection	Species	Locality	Nb. of colonies with cocoons	Total nb. of cocoons	Total nb. of larvae	% of cocoon infestation (exact proportion)
2015	*E. ruidum* sp. 2	Bajos de Coyula	4	111	2	0
	*E. ruidum* sp. 3	Yerba Santa	4	172	72	0.6 (1/172)
	*E. ruidum* sp. 4	El Zapotal	4	233	87	0
2016	*E. ruidum* sp. 2	Bajos de Coyula	14	487	74	0
	*E. ruidum* sp. 3	Yerba Santa	18	1285	794	0
	*E. ruidum* sp. 4	Puente Cuatode	17	1229	371	0.3 (4/1229)
		El Zapotal	9	717	116	0
		Piedras Negras	13	490	191	0
2017	*E. ruidum* sp. 4	El Zapotal	15	1071	478	0
Total			98	5795	2185	< 0.1 (5/5795)

A total of 36 nematodes were collected from mermithized workers: three individuals in three different colonies of *E. ruidum* sp. 2, 33 individuals in 11 different colonies of *E. ruidum* sp. 3, and none in *E. ruidum* sp. 4. No larva was parasitized for any of the three species, whilst at dissection eight cocoons were found parasitized by eucharitid wasps belonging to an undetermined species from the *Kapala* Cameron clade. Nine individuals of *Kapala* sp. were obtained: a male pupa from a cocoon of *E. ruidum* sp. 2; one fed planidium and one mature larva from two cocoons from a colony of *E. ruidum* sp. 3; one male pupa, two adult males (on the same host), two adult females, and one adult in poor condition whose sex could not be determined from five cocoons from four colonies of *E. ruidum* sp. 4 (see S1 Table).

**Fig 1:**
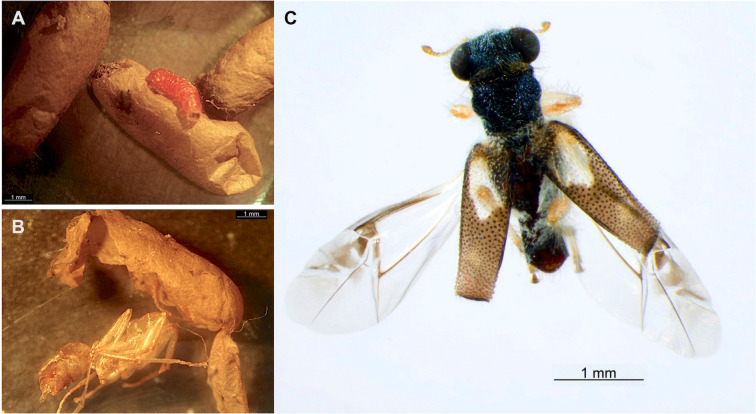
*Phyllobaenus obscurus* (Coleoptera: Cleridae). **(A)** a third-instar larva that has just emerged from the host cocoon; **(B)** remains of the *E. ruidum* sp. 3 pupa from which the mature beetle larva has emerged (the dissected cocoon can be observed); **(C)** *Phyllobaenus obscurus* adult reared from the larva that parasitized the pupa in (B). Photo credits: (A-B) Gabriela Pérez-Lachaud, (C) Humberto Bahena-Basave.

**Fig 2:**
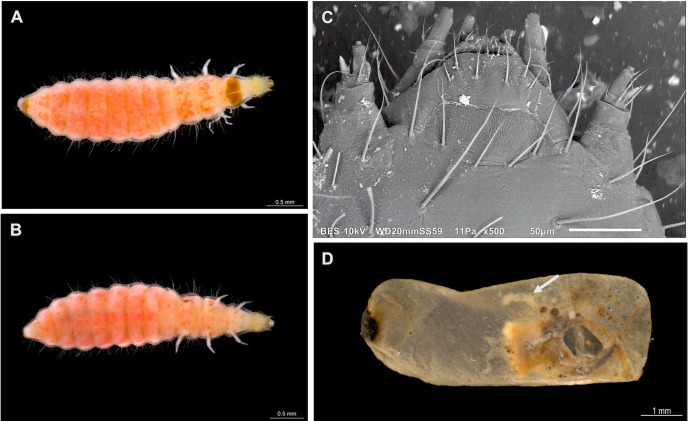
*Phyllobaenus obscurus* larvae and remains of the host within the host cocoon. **(A)** second-instar *P. obscurus* larva, dorsal view; **(B)** same, ventral view; **(C)** dorsal view of the head of a second-instar larva, low vacuum SEM photography; **(D)** an *E. ruidum* sp. 4 cocoon showing an exit hole after the departure of a *P. obscurus* larva (the ant pupa remains are visible, the white arrow pointing at a leg). Photo credits: (A,B,D) Humberto Bahena-Basave; (C) Gabriela Pérez-Lachaud and Manuel Elías-Gutiérrez.

In addition, five cocoons were parasitized by characteristic campodeiform beetle larvae (Table 3). In 2015, from a total of 516 cocoons maintained in observation, a single, pink-colored larva emerged 10 days later from isolated cocoons of a colony of *E. ruidum* sp. 3 collected at Ranchería Yerba Santa (representing 0.6% of cocoons of this species for that year, Fig 1A, S1 Video). The cocoon from which the larva had emerged showed signs of a single round exit hole. When it was dissected, the ant host pupa was found beheaded and only its empty exoskeleton remained (Fig 1B). The larva was isolated in a glass vial along with some fabric to help pupation and an adult belonging to a species of Cleridae (Insecta: Coleoptera) was reared. No other beetle specimens were obtained from the other cocoons after dissection. During the 2016 campaign, out of a total of 4,208 cocoons, four cocoons from *E. ruidum* sp. 4 collected at Puente Cuatode were parasitized by beetle larvae or presented signs of attack (Table 3). One second- and two third-instar larvae were obtained (Figs 2A–2C), each one found inside an intact cocoon on an ant pupa. Another cocoon presented a round hole, suggesting a larva had already left the host to pupate elsewhere and the remains of the host pupa were found inside (Fig 2D arrow). During 2017 only one site was sampled (El Zapotal, Mazunte) and 15 colonies of *E. ruidum* sp. 4 with 1071 cocoons were collected. No beetle or parasitoid wasp was found during this collecting campaign. Whatever the campaign, no eggs or triungulin larvae of Cleridae have ever been observed.

### Beetle parasitoid identity

**Fig 3:**
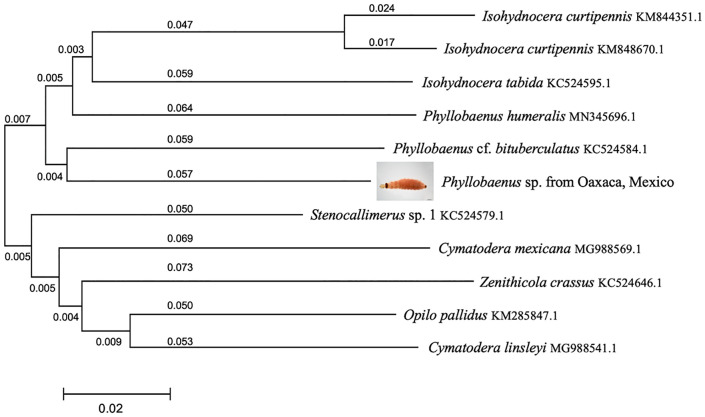
Illustrative neighbor-joining tree diagram showing the genetic relationship between our sample and the 10 most identical taxa from Blast, based on Kimura-2 genetic distances. Numbers indicate branch lengths. Species names are followed by Genbank accession numbers and correspond to those in Genbank platform; however, some species have been synonymized as follows: *Stenocallimerus* is a synonym of *Callimerus*, *Isohydnocera tabida* is a synonym of *Neohydnocera tabida*, and *Isohydnocera curtipennis* is a synonym of *Neohydnocera longicollis*. The scale refers to Kimura 2P distance. Photo credits: Humberto Bahena-Basave.

Based upon morphology, the adult could be identified as a specimen of *Phyllobaenus obscurus* (Gorham) (Cleridae: Hydnocerinae: Hydnocerini) (Fig 1C). The COI fragment supports assignment of the larva to the Cleridae, and is consistent with the genus *Phyllobaenus* (Fig 3). Blasting the sequence of our larva on the Genbank site returned the sequence of *Phyllobaenus* cf. *bituberculatus* (NLG-2013 voucher 4694, Genbank accession number KC524584.1) as the closest available sequence with 88.74% of identity (best score of 804). The genetic distance based on Kimura-2-parameter model between these two sequences is 0.116 ± 0.013. Unfortunately, the poor state of the beetle adult prevented extraction, and no sequence could be obtained.

## Discussion

Here we provide strong evidence that a checkered beetle behaves as parasitoid of the pupae of ants of the *E. ruidum* species complex in Oaxaca, Mexico. The adult was identified as *Phyllobaenus obscurus* based on morphology, while the COI fragment of the larva supports assignment to Cleridae and is consistent with *Phyllobaenus.* There is no public COI sequence for *P. obscurus* available for comparison with our larva and thus species level confirmation awaits a reference sequence for this taxon in the future. However, the evidence presented here supports a reliable connection between the larva and adult beetle based on morphology, rearing, and co-occurrence; so, the most parsimonious hypothesis is that the larvae and adult beetle found in this study belong to the same species of *Phyllobaenus*.

Although the eggs or triungulin larvae of the clerid beetle were not found in association with these ants, our observations showed that the larva of *P. obscurus* developed at the expense of a single ant pupa enclosed in an intact cocoon (neither pierced nor opened), resulting in the death of the host after the clerid larva had finished feeding. These facts do not correspond in any way to predation cases reported for many other beetles, such as rove beetles and other checkered beetles, where several hosts are preyed upon before development is completed. In our observations, within each cocoon, only one host was available and large enough to allow the development of a parasitoid the size of *P. obscurus*. The genus *Phyllobaenus*, the second largest Hydnocerini genus after *Callimerus* Gorham, comprises 114 described species [102–105]. Hydnocerini are considered general ant mimics, due either to their wasp-waisting color patterns, their elongate body shape (also sometimes creating a physical “waisting” effect), or their very movement patterns that are similar to those of an ant running quickly up and down plant substrates and changing directions erratically [32,106–108]. For example, *Neohydnocera aegra* (Newman) strikingly resembles a species of *Pseudomyrmex* Lund and has been found abundantly among these ants [109], and *Phyllobaenus cryptocerinus* (Gorham) is a reported mimic of *Cephalotes* Latreille ants (= *Cryptocerus* Latreille) [106], but no species from this clade has ever been reported as an intranidal symbiont of ants. Our findings constitute the first record of a beetle species adapted to parasitize ant brood within the defended boundaries of the nest of rather aggressive ants.

Like many of its congeners, *P. obscurus* is an active clerid with a highly variable color pattern and body size (2–5 mm). This species is known from Mexico, Guatemala, Costa Rica, and Panama [110–113]. Araujo et al. [114] included Brazil based on an unvouchered record with no data nor depository presented from a personal database (see “Unverified Distributions” in [115]). Prior to this study it was known only from Durango, Morelos and Tamaulipas in Mexico [116,117]. The data presented herein for Oaxaca and an unpublished record from Chiapas ([Verbatim label data] 3000’, “El Chorreadero” by Hwy. 190, 1.25 km E Tuxtla Gutierrez, under debris, gravel bank of stream in deep ravine, 25-XII-1972, H. Frania, FSCA, Florida State Collection of Arthropods) are new state records for Mexico. Despite the broad distribution of this rather common species, nothing was known about its natural history prior to our findings, and the larva and larval feeding habits were unknown.

Most specimens of *P. obscurus* have been collected by beating the foliage of live trees and occasionally shrubs (JML personal observations; accounts from numerous beetle collectors). Specimen label data indicate few specimens collected with other methods (e.g., lights, fogging, Malaise trap) (Leavengood, unpublished data). Notably, label data for the Chiapas new state record for Mexico (see above) indicate an adult was caught on the ground and under debris, which is most unusual. In the Western Hemisphere, hydnocerines are not observed on the ground, but usually high in foliage (e.g., [108]) and there are no records of *Phyllobaenus* collected in pitfall traps. Author JML has been presented two separate accounts of a *Phyllobaenus* specimen emerging from an ant hill in the ground. But no specimens were produced nor identifications made. So, with only one vouchered record (Chiapas, FSCA), two unvouchered events (ant hill emergence observations), and the five brood parasitoid observations noted herein (recorded in different years, at two different sites 65 km apart, and concerning two closely related but nevertheless different ant species), any record of *Phyllobaenus* at ground level (or below) is highly unusual, yet all but one such record are specifically associated with ants. Furthermore, it should be noted that our records concern clerid larvae that were sampled inside ant nests, in deep chambers, and developed on ant pupae enclosed in intact silken cocoons, which excludes the possibility of any accidental or fortuitous presence of the larvae within the ant nests.

Myrmecophilous taxa are not scattered randomly across the arthropod tree of life. Rather, their evolution is strongly biased to certain groups and, among insects, towards the Coleoptera which “*contains a large pool of lineages with the potential for transitioning to myrmecophily*” [27]. At least 33 of the beetle families, representing approximately one-fifth, have thus far been documented to include one or more taxa that exhibits close association with ant colonies [27]. Many of the most familiar beetle families contain at least one lineage that inhabits ant colonies, including the mainly aquatic beetle family Hydrophilidae [118]. Indeed, at least two species of *Sphaerocetum* Fikáček have been collected in mixed nests of *Camponotus* Mayr and *Crematogaster* Lund, and a larva of *S. arboreum* Fikáček et al. was collected in an ant nest along with the adults, implying the entire life cycle may take place inside the ant nest, suggesting that these beetles likely prey on ant larvae [118].

Ants and their nests are reliable, fixed resources in space and time [119]. Furthermore, ant brood in tropical species is present all year round so it is not surprising that a diverse array of parasitoids has adapted to ants as hosts, and new family-level records of species parasitizing ants or their brood are recorded from time to time (e.g., [10,120–122]). In addition, as suggested by Parker [27], several ecological constraints and phenotypic attributes may predispose some taxa to undergo the evolutionary transition from free-living to myrmecophily which, in combination, could explain the myrmecophile beetle bias: i) the huge species richness of Coleoptera, ii) their ecological predisposition to encountering and exploring ant colonies, and iii) their possession of a major protective preadaptation in the form of elytra. Additionally, the size of the myrmecophile relative to the ant host species (small myrmecophile species/large host), a limuloid body form, and other protective morpho-anatomical adaptations (i.e., pedofossae) are traits that facilitate the integration of some intruders into colony life (see [13,123–125]).

Although we clearly established that the larvae of this clerid develop as parasitoids, whether this species is a facultative or obligate parasitoid of the ant brood remains to be clarified. The line between predation, parasitoidism, and parasitism is often quite blurred, and some species may even switch between strategies depending on the context. For example, the mite *Macrodynichus sellnicki* (Hirschmann & Zirngiebl-Nicol) is a common parasitoid of the pupae of the invasive ant *Nylanderia fulva* (Mayr), but can also develop on a larger host, the native *E. ruidum* sp. 2 in Colombia [15]. While solitary attacks do not result in the death of the host, mites attacking in groups drain the host of its internal fluids and kill it. Therefore, the nature of the parasitic or parasitoidic relationship between *M. sellnicki* and *E. ruidum* sp. 2, is context dependent, varying according to the intensity of the attack. Similarly, the larvae of the chechered beetle *T. umbellatarum*, approaches the boundary between predation and parasitoidism [70,71], demonstrating here too a context-dependent change in lifestyle. Elucidating the lifestyle of *P. obscurus*, whether a facultative or obligate parasitoid, warrants further investigation.

Even though the prevalence of parasitism in certain ant parasitoids can sometimes reach high levels (e.g., [125–128]), extremely low prevalence levels, such as those observed in this study, are quite common among many highly specialized parasites and parasitoids of social insects. For example, only eight cocoons were found parasitized by *Kapala* eucharitid wasps in this study and various examples of extremely low prevalence of parasitism have previously been reported for many other ant associates [9,129–135]. Plausible explanations for rarity include low host encounter rate, strong host colony defenses, seasonality, or sampling detection limits, for example. In addition, given the broad geographic range of *P. obscurus* compared to the restricted distribution of *E. ruidum* sp. 3 and sp. 4, further research is needed to address the possibility of specialized local host use if the beetle uses different hosts across regions or if suitable conditions are patchy. Coevolution between ants and their rare, specialized parasitoids is intriguing because, by definition, a parasitoid kills its host. However, lethal outcomes for individual host ants rarely result in the destruction of the colony, and parasitoidism in ants resembles more to a chronic disease, allowing for stable coexistence between parasitoids and ants. This is certainly one of the reasons why so few parasites and parasitoids actually have the potential to act as biological control agents against pest ants.

Our finding of an unusual feeding mode for a checkered beetle is surprising but not entirely unexpected as other checkered beetles belonging to different tribes border the line between predator and parasitoid [42,51,52,57,70,71], likely depending on the relative size of their prey/host. It suggests that the parasitoid lifestyle may have evolved several times within the Cleridae. Notably, our record is also the first evidence of beetles parasitizing successfully the immature stages of ants within their subterranean nests, suggesting a major evolutionary shift in the biology of Hydnocerini. Our results further support a previously stated hypothesis about the evolution of ant parasitoidism in clades with predatory habits. As suggested for other unusual ant parasitoids such as the microdontine syrphid fly *Hypselosyrphus trigonus* Hull [121], the only parasitoid species among this clade of predators, it is likely that parasitoidism in *P. obscurus* evolved from a predatory ancestor. According to Godfray [136], the transition from a predatory to a parasitoid lifestyle may be straightforward since a predator that requires only a single prey item for its development is, by definition, a parasitoid.

There are still some gaps in knowledge regarding the biology of this clerid. Like other ant parasitoids such as eucharitid wasps of the genera *Kapala*, *Isomerala* Shipp, *Obeza* Heraty, or *Pseudochalcura* Ashmead [8], *P. obscurus* develops at the expense of host larvae/pupae under the protection of a cocoon, avoiding deadly interactions with their host ants. But, as our observations show, when larval development is completed, the larva exits the host cocoon to pupate. Clerids are known to commonly pupate near their prey (e.g., in galleries of their prey, or tree crevices nearby or inside the host pupal cell [57,58]), but the site of pupation of *P. obscurus* remains to be uncovered. Its high mobility and small size (only 2–5 mm for the adult) relative to the *Ectatomma* hosts (8–9 mm) may help avoiding ant aggressiveness. Infiltration into the ant nest is probably by phoresis of the mobile first-instar larva (triungulin) as occurs in other ant parasitoids which lay eggs away from the host [7,20,22], but this needs confirmation.

Although some checkered beetle species are considered of importance as potential biological control agents, there are very few rearing records for Cleridae in general, and data on their natural life history are scarce (Table 1). However, results of laboratory and field studies underscore the plasticity and adaptability in the food habits of Cleridae (e.g., [54] and references in Table 1). The shift in foraging behavior reported here, from predation towards parasitoidism, is likely to confirm the diversified feeding traits within the Cleridae.

## Supporting information

S1 Video A *Phyllobaenus obscurus* larva that has just emerged from its *Ectatomma ruidum* sp. 3 host cocoon. (MOV)

S1 Table Detailed collection data for the three species of the *Ectatomma ruidum* complex studied: species concerned, location, date of collection, colony composition. µQ: number of microgynes.
(XLSX)

## References

[1] Waage JK, Hassell MP. Parasitoids as biological control agents – a fundamental approach. Parasitology. 1982;84(4):241–68. doi: 10.1017/s003118200005366x

[2] Greany PD, Vinson SB, Lewis WJ. Insect Parasitoids: Finding New Opportunities for Biological Control. BioScience. 1984;34(11):690–6. doi: 10.2307/1309661

[3] Koller J, Sutter L, Gonthier J, Collatz J, Norgrove L. Entomopathogens and Parasitoids Allied in Biocontrol: A Systematic Review. Pathogens. 2023;12(7):957. doi: 10.3390/pathogens12070957 37513804 PMC10383084

[4] Eggleton P, Belshaw R. Insect parasitoids: an evolutionary overview. Philosophical Transactions of the Royal Society of London Series B: Biological Sciences. 1992;337(1279):1–20. doi: 10.1098/rstb.1992.0079

[5] Quicke DLJ. Parasitic wasps. London: Chapman & Hall; 1997.

[6] Feener DHJ, Brown BV. Diptera as parasitoids. Annu Rev Entomol. 1997;42:73–97. doi: 10.1146/annurev.ento.42.1.73 15012308

[7] Kathirithamby J. Host-parasitoid associations in Strepsiptera. Annu Rev Entomol. 2009;54:227–49. doi: 10.1146/annurev.ento.54.110807.090525 18817508

[8] Lachaud JP, Pérez-Lachaud G. Diversity of species and behavior of hymenopteran parasitoids of ants: a review. Psyche. 2012;2012:134746. doi: 10.1155/2012/134746

[9] Pérez-Lachaud G, Lachaud J-P. Arboreal ant colonies as “hot-points” of cryptic diversity for myrmecophiles: the weaver ant Camponotus sp. aff. textor and its interaction network with its associates. PLoS One. 2014;9(6):e100155. doi: 10.1371/journal.pone.0100155 24941047 PMC4062527

[10] González CT, Wcislo WT, Cambra R, Wheeler TA, Fernández-Marín H. A new ectoparasitoid species of Pseudogaurax Malloch, 1915 (Diptera: Chloropidae), attacking the fungus-growing ant, Apterostigma dentigerum Wheeler, 1925 (Hymenoptera: Formicidae). Ann Entomol Soc Am. 2016;109(4):639–45. doi: 10.1093/aesa/saw023

[11] Pérez-Lachaud G, Jahyny BJB, Ståhls G, Rotheray G, Delabie JHC, Lachaud J-P. Rediscovery and reclassification of the dipteran taxon Nothomicrodon Wheeler, an exclusive endoparasitoid of gyne ant larvae. Sci Rep. 2017;7:45530. doi: 10.1038/srep45530 28361946 PMC5374537

[12] Poinar G Jr. Nematode parasites and associates of ants: past and present. Psyche. 2012;2012:192017. doi: 10.1155/2012/192017

[13] Lachaud J-P, Klompen H, Pérez-Lachaud G. Macrodinychus mites as parasitoids of invasive ants: an overlooked parasitic association. Sci Rep. 2016;6:29995. doi: 10.1038/srep29995 27444515 PMC4956750

[14] Brückner A, Klompen H, Bruce AI, Hashim R, von Beeren C. Infection of army ant pupae by two new parasitoid mites (Mesostigmata: Uropodina). PeerJ. 2017;5:e3870. doi: 10.7717/peerj.3870 29038753 PMC5639873

[15] Pérez-Lachaud G, Klompen H, Poteaux C, Santamaría C, Armbrecht I, Beugnon G, et al. Context dependent life-history shift in Macrodinychus sellnicki mites attacking a native ant host in Colombia. Sci Rep. 2019;9(1):8394. doi: 10.1038/s41598-019-44791-2 31182745 PMC6557818

[16] Vander Meer RK, Jouvenaz DP, Wojcik DP. Chemical mimicry in a parasitoid (Hymenoptera: Eucharitidae) of fire ants (Hymenoptera: Formicidae). J Chem Ecol. 1989;15(8):2247–61. doi: 10.1007/BF01014113 24272384

[17] Pérez-Lachaud G, Bartolo-Reyes JC, Quiroa-Montalván CM, Cruz-López L, Lenoir A, Lachaud J-P. How to escape from the host nest: imperfect chemical mimicry in eucharitid parasitoids and exploitation of the ants’ hygienic behavior. J Insect Physiol. 2015;75:63–72. doi: 10.1016/j.jinsphys.2015.03.003 25770980

[18] Gómez Durán J-M, van Achterberg C. Oviposition behaviour of four ant parasitoids (Hymenoptera, Braconidae, Euphorinae, Neoneurini and Ichneumonidae, Hybrizontinae), with the description of three new European species. Zookeys. 2011;(125):59–106. doi: 10.3897/zookeys.125.1754 21998538 PMC3185369

[19] Clausen CP. Entomophagous insects. 1st ed. New York: McGraw-Hill; 1940.

[20] Clausen CP. The oviposition habits of the Eucharidae (Hymenoptera). J Wash Acad Sci. 1940;30(12):504–16.

[21] Heraty JM. A revision of the genera of Eucharitidae (Hymenoptera: Chalcidoidea) of the world. Mem Amer Ent Inst. 2002;68:1–359.

[22] Carey B, Visscher K, Heraty J. Nectary use for gaining access to an ant host by the parasitoid Orasema simulatrix (Hymenoptera, Eucharitidae). J Hym Res. 2012;27:47–65. doi: 10.3897/jhr.27.3067

[23] Herreid JS, Heraty JM. Hitchhikers at the dinner table: a revisionary study of a group of ant parasitoids (Hymenoptera: Eucharitidae) specializing in the use of extrafloral nectaries for host access. Syst Entomol. 2017;42(1):204–29. doi: 10.1111/syen.12206

[24] Mathis KA, Philpott SM. Current understanding and future prospects of host selection, acceptance, discrimination, and regulation of phorid fly parasitoids that attack ants. Psyche. 2012;2012:895424. doi: 10.1155/2012/895424

[25] Mathis KA, Tsutsui ND. Cuticular Hydrocarbon Cues Are Used for Host Acceptance by Pseudacteon spp. Phorid Flies that Attack Azteca sericeasur Ants. J Chem Ecol. 2016;42(4):286–93. doi: 10.1007/s10886-016-0694-y 27130489

[26] Eggleton P, Belshaw R. Comparisons of dipteran, hymenopteran and coleopteran parasitoid: provisional phylogenetic explanations. Biol J Linn Soc. 1993;48(3):213–26. doi: 10.1006/bijl.1993.1015

[27] Parker J. Myrmecophily in beetles (Coleoptera): evolutionary patterns and biological mechanisms. Myrmecol News. 2016;22:65–108. doi: 10.25849/myrmecol.news_022:065

[28] Alpert GD, Ritcher PO. Notes on the Life Cycle and Myrmecophilous Adaptations of Cremastocheilus Armatus(Coleoptera: Scarabaeidae). Psyche: A Journal of Entomology. 1975;82(3–4):283–91. doi: 10.1155/1975/79470

[29] Geiselhardt SF, Peschke K, Nagel P. A review of myrmecophily in ant nest beetles (Coleoptera: Carabidae: Paussinae): linking early observations with recent findings. Naturwissenschaften. 2007;94(11):871–94. doi: 10.1007/s00114-007-0271-x 17563864

[30] Mathis KA, Tsutsui ND. Dead ant walking: a myrmecophilous beetle predator uses parasitoid host location cues to selectively prey on parasitized ants. Proc Biol Sci. 2016;283(1836):20161281. doi: 10.1098/rspb.2016.1281 27512148 PMC5013774

[31] Chapman EG, Leavengood JM, Dupuis JR. The Cleridae and Thanerocleridae of Eastern North America, with Illustrated Keys, Updated Distributions, and Special Emphasis on the Kentucky Fauna. Zootaxa. 2025;5639(1):1–88. doi: 10.11646/zootaxa.5639.1.1 41119132

[32] Leavengood JM Jr. The checkered beetles (Coleoptera: Cleridae) of Florida. M.Sc. Thesis, University of Florida. 2008. Available from: http://etd.fcla.edu/UF/UFE0023794/leavengood_j.pdf

[33] Parker FD. Nests and nest associates of two “red” Odynerus wasps (Hymenoptera: Eumenidae). J Kansas Entomol Soc. 1984;57(3):521–5.

[34] Parker FD, Bohart RM. Host parasite associations in some twig-nesting Hymenoptera from Western North America. Part II. Pan-Pac Entomol. 1968;44(1):1–6.

[35] MacSwain JW. Notes on the habits of the predator Cymatodera ovipennis Say with a description of the pupa. Pan-Pac Entomol. 1945;21(3):97–100.

[36] Balduf WV. On the habits and development of a checkered beetle (Cymatodera undulata Say, Cleridae). Trans Am Entomol Soc. 1926;52(1):29–37.

[37] Eliason EA, Potter DA. Biology of Callirhytis cornigera (Hymenoptera: Cynipidae) and the arthropod community inhabiting its galls. Environmental Entomology. 2000;29(3):551–9. doi: 10.1603/0046-225X-29.3.551

[38] Gosling DCL. An Annotated List of the Checkered Beetles (Coleoptera: Cleridae) of Michigan. The Great Lakes Entomologist. 2025;13(2):65–76. doi: 10.22543/0090-0222.2551

[39] Krombein KV. Trap-Nesting Wasps and Bees: Life Histories, Nests, and Associates. Washington, D.C., Smithsonian Press; 1967.

[40] Bitner RM. Predation by the larvae of Lecontella cancellata (LeConte) (Coleoptera: Cleridae) on seven species of aculeate Hymenoptera. Entomological News. 1972;83(1):23–6.

[41] Knull JN. Notes on Coleoptera.—No. 3. Entomological News. 1932;43(2):42–5.

[42] Rau P. A note on Lecontella cancellata Lec. (Coleoptera: Cleridae) in cells of the mud-daubing wasp. Entomological News. 1944;55(8):197.

[43] Linsley EG. Studies in the genus Aulicus Spinola (Coleoptera-Cleridae). Univ Calif Publ Ent. 1936;6:249–62.

[44] Mawdsley JR. Ecological Notes on Species of Cleridae (Insecta: Coleoptera) Associated With the Prairie Flora of Central North America. The Great Lakes Entomologist. 2018;35(1). doi: 10.22543/0090-0222.2056

[45] Price JR. Post-release assessment of classical biological control of Canada thistle (*Cirsium arvense*) in the western United States. M.Sc. Thesis, University of Idaho. 2014. Available from https://objects.lib.uidaho.edu/etd/pdf/Price_idaho_0089M_10185.pdf

[46] Eikenbary RD, Fox RC. Arthropod predators of the Nantucket pine tip moth, Rhyacionia frustrana. Ann Entomol Soc Am. 1968;61(5):1218–21. doi: 10.1093/aesa/61.5.1218

[47] Powell JA. Prolonged diapause in Enoclerus zonatus (Cleridae). Coleopt Bull. 1975;29(1):44.

[48] Foster DE, Barr WF. Notes on the distribution and bionomics of some North American Cleridae. J Kansas Entomol Soc. 1972;45(1):122–5.

[49] Balduf WV. The bionomics of entomophagous Coleoptera. St Louis. 1935.

[50] Böving AG, Champlain AB. Larvae of North American beetles of the family Cleridae. Proc US Natl Mus. 1920;57(2323):575–649. doi: 10.5479/si.00963801.57-2323.575

[51] Foster DE. Revision of North American Trichodes (Herbst) (Coleoptera: Cleridae). Spec Publ Mus Texas Tech Univ. 1976;11:1–86.

[52] Essig EO. Trichodes ornatus Say. J Econ Entomol. 1934;27:724.

[53] Linsley EG. Notes on the habits of some beetles from the vicinity of Yosemite National Park. Bull So Calif Acad Sci. 1942;41:164–6.

[54] Linsley EG, MacSwain JW. Observations on the life history of Trichodes ornatus (Coleoptera, Cleridae), a larval predator in the nests of bees and wasps. Ann Entomol Soc Am. 1943;36(4):589–601. doi: 10.1093/aesa/36.4.589

[55] Davidson A. On the nesting habits of Anthidium consimile. Entomological News. 1896;7(1):22–6.

[56] Baker CF. Biological notes on some Colorado Coleoptera. Entomological News. 1895;6(1):27–9.

[57] Sabrosky CW. Notes on the larva and larval habit of Isohydnocera curtipennis (Newmn.) (Coleoptera, Cleridae). J Kansas Entomol Soc. 1934;7(2):65–8.

[58] Swenson SJ, Bell-Clement J, Schroeder S, Prischmann-Voldseth DA. Predatory and parasitic insects associated with Urophora cardui L. (Diptera: Tephritidae) galls on Canada thistle, Cirsium arvense L. (Asterales, Asteraceae) in North Dakota. Insects. 2022;13:646. doi: 10.3390/insects13070646 35886822 PMC9316070

[59] Foster DE. A new species of Phyllobaenus (Coleoptera: Cleridae). J Kansas Entol Soc. 1981;54(3):661–4.

[60] Goeden RD, Ricker DW, Müller H. Introduction, recovery, and limited establishment of Coleophora klimeschiella (Lepidoptera: Coleophoridae) on Russian thistles, Salsola australis, in Southern California. Environmental Entomology. 1987;16(4):1027–9. doi: 10.1093/ee/16.4.1027

[61] Hawkins BA, Goeden RD. Organization of a parasitoid community associated with a complex of galls on Atriplex spp. in southern California. Ecol Entomol. 1984;9(3):271–92. doi: 10.1111/j.1365-2311.1984.tb00851.x

[62] Baine Q, Casares EE, Hughes DWW, Martinson VG, Martinson EO. Arthropod communities associated with gall-inducing Aciurina bigeloviae and Aciurina trixa (Diptera: Tephritidae) in New Mexico. Ann Entomol Soc Am. 2024;117(2):107–17. doi: 10.1093/aesa/saad037

[63] Morrill WL, Weaver DK, Irish NJ, Barr WF. Phyllobaenus dubius (Wolcott) (Coleoptera: Cleridae), a new record of a predator of the wheat stem sawfly (Hymenoptera: Cephidae). J Kansas Entomol Soc. 2001;74(3):181–3.

[64] Farrar RR. A new record of the clerid, Phyllobaenus pubescens (LeConte) (Coleoptera), as a predator of Heliothis zea (Boddie) (Lepidoptera: Noctuidae) on cotton. Coleopt Bull. 1985;39(1):33–4.

[65] Pierce WD, Cushman RA, Hood CE, Hunter WD. The insect enemies of the cotton boll weevil. USDA Bur Entomol Bull. 1912;100:1–99.

[66] Rogers CE, Perkins WD, Seiler GT. Bionomics of Stibadium spumosum (Lepidoptera: Noctuidae), a pest of sunflower in the southern plains. Environ Entomol. 1986;15(6):1275–8. doi: 10.1093/ee/15.6.1275

[67] Coquillet DW. Notes on the habits of some California Coleoptera. Insect Life. 1912;4:260–2.

[68] Tilden JW. Notes on the habits of Phyllobaenus scabra (Le Conte) (Cleridae). Coleopt Bull. 1950;4(2):17–9.

[69] Osten Sacken R. On the cynipidae of North American oaks and their galls. Proc Entomol Soc Phil. 1861;1:47–72.

[70] Cros A. Le trichodes umbellatarum Ol., ses moeurs, son évolution. Bull Mus Natl Hist Nat. 1908;14:215–21.

[71] Cros A. Note sur le Trichodes umbellatarum Ol. Bull Soc Hist Nat Afr Nord. 1911;2(7–9):111–5, 135–40, 142–9.

[72] Leavengood JM Jr, Pinkerton MG, Rifkind J. Description of the new genus Tarsobaenus and three new species from Costa Rica (Coleoptera: Cleridae: Clerinae: Hydnocerini). Zootaxa. 2022;5138(2):167–76. doi: 10.11646/zootaxa.5138.2.4 36101029

[73] Letourneau DK. Code of ant-plant mutualism broken by parasite. Science. 1990;248(4952):215–7. doi: 10.1126/science.248.4952.215 17740138

[74] Brown WLJ. Contributions toward a reclassification of the Formicidae. II. Tribe Ectatommini (Hymenoptera). Bull Mus Comp Zool. 1958;118(5):175–362.

[75] Kugler C, Brown WLJ. Revisionary & other studies on the ant genus Ectatomma, including the descriptions of two new species. Search: Agriculture. 1982;24:1–8.

[76] Nettel-Hernanz A, Lachaud J-P, Fresneau D, Lopez- Muñoz RA, Poteaux C. Biogeography, cryptic diversity, and queen dimorphism evolution of the Neotropical ant genus Ectatomma Smith 1858 (Formicidae: Ectatomminae). Org Divers Evol. 2015;15(3):543–53. doi: 10.1007/s13127-015-0215-9

[77] Aguilar-Velasco RG, Poteaux C, Meza-Lázaro R, Lachaud J-P, Dubovikoff D, Zaldívar-Riverón A. Uncovering species boundaries in the Neotropical ant complex Ectatomma ruidum (Ectatomminae) under the presence of nuclear mitochondrial paralogues. Zool J Linn Soc. 2016;178(2):226–40. doi: 10.1111/zoj.12407

[78] Breed MD, Abel P, Bleuze TJ, Denton SE. Thievery, home ranges, and nestmate recognition inEctatomma ruidum. Oecologia. 1990;84(1):117–21. doi: 10.1007/BF00665604 28312784

[79] De Carli P, Lachaud J-P, Beugnon G, López-Méndez JA. Études en milieu naturel du comportement de cleptobiose chez la fourmi néotropicale Ectatomma ruidum (Hymenoptera, Ponerinae). Actes Coll Insectes Soc. 1998;11:29–32.

[80] Guénard B, McGlynn TP. Intraspecific Thievery in the Ant Ectatomma ruidum is Mediated by Food Availability. Biotropica. 2013;45(4):497–502. doi: 10.1111/btp.12031

[81] Meza-Lázaro RN, Poteaux C, Bayona-Vásquez NJ, Branstetter MG, Zaldívar-Riverón A. Extensive mitochondrial heteroplasmy in the neotropical ants of the Ectatomma ruidum complex (Formicidae: Ectatomminae). Mitochondrial DNA A DNA Mapp Seq Anal. 2018;29(8):1203–14. doi: 10.1080/24701394.2018.1431228 29385929

[82] Meza-Lázaro RN, Peña-Carrillo KI, Poteaux C, Lorenzi MC, Wetterer JK, Zaldívar-Riverón A. Genome and cuticular hydrocarbon-based species delimitation shed light on potential drivers of speciation in a Neotropical ant species complex. Ecol Evol. 2022;12(3):e8704. doi: 10.1002/ece3.8704 35342602 PMC8928884

[83] Peña-Carrillo KI, Poteaux C, Leroy C, Meza-Lázaro RN, Lachaud J-P, Zaldívar-Riverón A, et al. Highly divergent cuticular hydrocarbon profiles in the cleptobiotic ants of the Ectatomma ruidum species complex. Chemoecology. 2020;31(2):125–35. doi: 10.1007/s00049-020-00334-0

[84] Peña Carrillo KI, Lorenzi MC, Brault M, Devienne P, Lachaud J-P, Pavan G, et al. A new putative species in the Ectatomma ruidum complex (Formicidae: Ectatomminae) produces a species-specific distress call. Bioacoustics. 2021;31(3):332–47. doi: 10.1080/09524622.2021.1938226

[85] McGlynn TP, Graham R, Wilson J, Emerson J, Jandt JM, Jahren AH. Distinct types of foragers in the ant Ectatomma ruidum: typical foragers and furtive thieves. Anim Behav. 2015;109:243–7. doi: 10.1016/j.anbehav.2015.08.024

[86] Lachaud JP. Ectatomma. In: Starr CK, editor. Encyclopedia of Social Insects. Cham: Springer; 2021. p. 358–65. doi: 10.1007/978-3-319-90306-4_41-1

[87] Santamaría C, Armbrecht I, Lachaud J-P. Nest architecture and colony composition in two populations of Ectatomma ruidum sp. 2 (E. ruidum species complex) in southwestern Colombia. PLoS One. 2022;17(2):e0263382. doi: 10.1371/journal.pone.0263382 35108326 PMC8809609

[88] Lachaud J-P, Fresneau D, García - Pérez J. Étude des stratégies d’approvisionnement chez 3 espèces de fourmis ponérines (Hymenoptera, Formicidae). Folia Entomol Mex. 1984;61:159–77.

[89] Lachaud JP. Foraging activity and diet in some neotropical ponerine ants. I. Ectatomma ruidum Roger (Hymenoptera, Formicidae). Folia Entomol Mex. 1990;78:241–56.

[90] Lachaud JP. Recruitment by selective activation: an archaic type of mass recruitment in a ponerine ant (Ectatomma ruidum). Sociobiology. 1985;11(2):133–42.

[91] Schatz B, Lachaud J-P. Effect of high nest density on spatial relationships in two dominant ectatommine ants (Hymenoptera: Formicidae). Sociobiology. 2008;51(3):623–43.

[92] Lachaud JP, Cadena A, Schatz B, Pérez-Lachaud G, Ibarra-Núñez G. Queen dimorphism and reproductive capacity in the ponerine ant, Ectatomma ruidum Roger. Oecologia. 1999;120(4):515–23. doi: 10.1007/s004420050885 28308301

[93] Lenoir J-C, Lachaud J-P, Nettel A, Fresneau D, Poteaux C. The role of microgynes in the reproductive strategy of the neotropical ant Ectatomma ruidum. Naturwissenschaften. 2011;98(4):347–56. doi: 10.1007/s00114-011-0774-3 21380620

[94] Lachaud JP, Pérez-Lachaud G. Ectaheteromorph ants also host highly diverse parasitic communities: a review of parasitoids of the Neotropical genus Ectatomma. Insect Soc. 2015;62(2):121–32. doi: 10.1007/s00040-015-0390-x

[95] García E. Modificaciones al sistema de clasificación climática de Köppen (para adaptarlo a las condiciones de la república Mexicana). 5th ed. Mexico: Instituto de Geografía. UNAM; 2004.

[96] Salas-Morales SH, Meave JA. Elevational patterns in the vascular flora of a highly diverse region in southern Mexico. Plant Ecol. 2012;213(8):1209–20. doi: 10.1007/s11258-012-0077-6

[97] Silva TSR, Silva-Freitas JM, Schoeninger K. A synopsis of the Brazilian Eucharitidae (Hymenoptera: Chalcidoidea) fauna: an annotated checklist of the family in the country, with a revised key for the New World genera. Zootaxa. 2019;4564(2):zootaxa.4564.2.3. doi: 10.11646/zootaxa.4564.2.3 31716502

[98] Ivanova NV, Dewaard JR, Hebert PDN. An inexpensive, automation‐friendly protocol for recovering high‐quality DNA. Molecular Ecology Notes. 2006;6(4):998–1002. doi: 10.1111/j.1471-8286.2006.01428.x

[99] Prosser S, Martínez-Arce A, Elías-Gutiérrez M. A new set of primers for COI amplification from freshwater microcrustaceans. Mol Ecol Resour. 2013;13(6):1151–5. doi: 10.1111/1755-0998.12132 23795700

[100] Andrews S. FastQC: A quality control tool for high throughput sequence data. 2010. Available from: http://www.bioinformatics.babraham.ac.uk/projects/fastqc/

[101] Kumar S, Stecher G, Tamura K. MEGA7: Molecular Evolutionary Genetics Analysis Version 7.0 for Bigger Datasets. Mol Biol Evol. 2016;33(7):1870–4. doi: 10.1093/molbev/msw054 27004904 PMC8210823

[102] Leavengood JMJ. Nomenclatural and distributional notes regarding some North American species of Phyllobaenus (Coleoptera: Cleridae: Hydnocerinae). G It Ent. 2014;13(59):471–80.

[103] Leavengood JM Jr. Phyllobaenus thomasi and P. turnbowi, two new species from Mexico and Belize (Coleoptera: Cleridae: Hydnocerinae: Hydnocerini). Insecta Mundi. 2020;0833:1–6.

[104] Leavengood JM Jr. Living a lie: The truth behind *Isohydnocera curtipennis* (Newman) and other historically confused species of *Phyllobaenus* Dejean, with the description of the new genus *Neohydnocera* (Coleoptera: Cleridae: Clerinae: Hydnocerini: Hydnocerina). Insecta Mundi. 2025;1105:1–17. https://digitalcommons.unl.edu/insectamundi/1613

[105] Leavengood JM, Rifkind J. Phyllobaenus inusitatotibialis, a new species from southern Arizona (Coleoptera: Cleridae: Hydnocerinae: Hydnocerini). Proceedings of the Entomological Society of Washington. 2020;122(3):673–7. doi: 10.4289/0013-8797.122.3.673

[106] Gorham HS. Insecta. Coleoptera. Malacodermata. Cleridae. In: Godman FD, Salvin O, editors. Biologia Centrali-Americana. London: Dulau; 1882;3(2):113–68.

[107] Mawdsley JR. Mimicry in Cleridae (Coleoptera). Coleopt Bull. 1994;48(2):115–25.

[108] Leavengood JM Jr, Gemmill RM, Raber B, Quinn MA. Notes on the identification, distribution and natural history of Phyllobaenus corticinus (Gorham, 1883) (Coleoptera: Cleridae: Hydnocerinae) including the first report from the United States. Coleopt Bull. 2012;66(4):351–6. doi: 10.1649/072.066.0410

[109] Wheeler AGJ, Stocks IC. Wolcottia (= Isohydnocera) aegra (Coleoptera: Cleridae): association with grasses (Poaceae), native Spartina spp. and the introduced Eragrostis curvula, and resemblance to co-occurring pseudomyrmecine ants. Fla Entomol. 2009;92(4):648–52. doi: 10.1653/024.092.0419

[110] Gorham HS. Insecta. Coleoptera. Malacodermata. Cleridae. In: Godman FD, Salvin O, editors. Biologia Centrali-Americana. London: Dulau; 1883. p. 169–93.

[111] Wolcott AB. A review of the Cleridae of Costa Rica. Coleopt Contrib. 1927;1(10):1–104.

[112] Leavengood JMJ, Garner BH. Nomenclatural notes on some checkered beetle (Coleoptera: Cleridae) types of the Natural History Museum, London (BMNH). Zootaxa. 2014;3760(3):301–35. doi: 10.11646/zootaxa.3760.3.1 24870084

[113] Toledo-Hernández VH, Rifkind J, Corona-López AM, Flores-Palacios A, Leavengood JMJ. Faunistic composition of Cleridae (Coleoptera) in El Limón de Cuauchichinola, Morelos, Mexico. Ann Entomol Soc Am. 2015;108(5):771–6. doi: 10.1093/aesa/sav039

[114] Araujo Castillo S, Romero Napoles J, Rifkind J, Flores Dávila M, García Martínez O, Cerna Chávez E. Biodiversidad de la familia Cleridae (Coleoptera: Cleroidea) de México. Folia Entomol Mex Nueva Ser. 2024;10:20241001. doi: 10.53749/fem.2024.10.01

[115] Leavengood JMJ. Corrections and additions to the Hydnocerina (Coleoptera: Cleridae: Clerinae: Hydnocerini) of Mexico. Insecta Mundi. 2024;1054:1–5.

[116] Hernández-May MA, Pérez-De la Cruz M, Toledo-Hernández VH, Rifkind J, Leavengood JMJ, Guzmán-Vega GA, et al. Preliminary survey of the checkered beetles (Coleoptera: Cleridae) of Tamaulipas, Mexico. Coleopt Bull. 2021;75(3):661–9. doi: 10.1649/0010-065X-75.3.661

[117] Hernández-May MA, Leavengood JMJ, Gerónimo-Torres JDC, Pérez-De La Cruz M, Niño-Maldonado S. New records of Phyllobaenus Dejean (Coleoptera: Cleridae: Clerinae: Hydnocerini) from Mexico. Southwest Entomologist. 2022;47(4):865–70. doi: 10.3958/059.047.0409

[118] Fikáček M, Maruyama M, Komatsu T, von Beeren C, Vondráček D, Short AEZ. Protosternini (Coleoptera: Hydrophilidae) corroborated as monophyletic and its larva described for the first time: a review of the myrmecophilous genus Sphaerocetum. Invertebr Syst. 2015;29(1):23–36. doi: 10.1071/IS14026

[119] Hölldobler B, Wilson EO. The ants. Cambridge, MA: Harvard University Press; 1990.

[120] Pérez-Lachaud G, Noyes J, Lachaud J-P. First record of an encyrtid wasp (Hymenoptera: Chalcidoidea) as true primary parasitoid of ants (Hymenoptera: Formicidae). Fla Entomol. 2012;95(4):1066–76.

[121] Pérez-Lachaud G, Jervis MA, Reemer M, Lachaud J-P. An unusual, but not unexpected, evolutionary step taken by syrphid flies: the first record of true primary parasitoidism of ants by Microdontinae. Biol J Linn Soc. 2014;111(2):462–72. doi: 10.1111/bij.12220

[122] Mekonnen B, Haran J, Copeland RS, Pirk CWW, Yusuf AA, Deletre E. First report of a gall midge as a parasitoid of weaver ants. Entomol Gen. 2020;40(4):437–41. doi: 10.1127/entomologia/2020/0908

[123] Kistner DH. The social insects’ bestiary. In: Hermann HR, editor. Social insects. New York: Academic Press; 1982. p. 1–244.

[124] von Beeren C, Brückner A, Hoenle PO, Ospina-Jara B, Kronauer DJC, Blüthgen N. Multiple phenotypic traits as triggers of host attacks towards ant symbionts: body size, morphological gestalt, and chemical mimicry accuracy. Front Zool. 2021;18(1):46. doi: 10.1186/s12983-021-00427-8 34538256 PMC8451089

[125] Pérez-Lachaud G, Degallier N, Gomy Y, Elías-Gutiérrez M, Rocha FH, Lachaud J-P. Cohabitation with aggressive hosts: description of a new microhisterid species in nests of a ponerine ant with ecological notes. Sci Rep. 2023;13(1):18484. doi: 10.1038/s41598-023-45692-1 37898669 PMC10613264

[126] Heraty JM. Biology and importance of two eucharitid parasites of *Wasmannia* and *Solenopsis*. In: Williams D, editor. Exotic ants: biology, impact, and control of introduced species. Boulder: Westwiew Press; 1994. p. 104–20.

[127] Orr MR, Dahlsten DL, Benson WW. Ecological interactions among ants in the genus Linepithema, their phorid parasitoids, and ant competitors. Ecol Entomol. 2003;28(2):203–10. doi: 10.1046/j.1365-2311.2003.00506.x

[128] Lachaud J-P, Pérez-Lachaud G. Impact of natural parasitism by two eucharitid wasps on a potential biocontrol agent ant in southeastern Mexico. Biological Control. 2009;48(1):92–9. doi: 10.1016/j.biocontrol.2008.09.006

[129] Lachaud JP, Passera L. Données sur la biologie de trois Diapriidæ myrmécophiles: Plagiopria passerai Masner, Solenopsia imitatrix Wasmann et Lepidopria pedestris Kieffer. Insect Soc. 1982;29(4):561–8. doi: 10.1007/BF02224225

[130] Hansson C, Lachaud J-P, Pérez-Lachaud G. Entedoninae wasps (Hymenoptera, Chalcidoidea, Eulophidae) associated with ants (Hymenoptera, Formicidae) in tropical America, with new species and notes on their biology. Zookeys. 2011;(134):65–82. doi: 10.3897/zookeys.134.1653 22140342 PMC3229211

[131] von Beeren C, Maruyama M, Hashim R, Witte V. Differential host defense against multiple parasites in ants. Evol Ecol. 2011;25(2):259–76. doi: 10.1007/s10682-010-9420-3

[132] Pérez-Lachaud G, Lachaud J-P. Hidden biodiversity in entomological collections: The overlooked co-occurrence of dipteran and hymenopteran ant parasitoids in stored biological material. PLoS One. 2017;12(9):e0184614. doi: 10.1371/journal.pone.0184614 28926617 PMC5604966

[133] Pérez-Lachaud G, Lachaud J-P. Co-occurrence in ant primary parasitoids: a Camponotus rectangularis colony as host of two eucharitid wasp genera. PeerJ. 2021;9:e11949. doi: 10.7717/peerj.11949 34466288 PMC8380026

[134] Rocha FH, Lachaud J-P, Pérez-Lachaud G. Myrmecophilous organisms associated with colonies of the ponerine ant Neoponera villosa (Hymenoptera: Formicidae) nesting in Aechmea bracteata bromeliads: a biodiversity hotspot. Myrmecol News. 2020;30:73–92. doi: 10.25849/myrmecol.news_030:073

[135] Pérez-Lachaud G, Rocha FH, Pozo C, Kaminski LA, Seraphim N, Lachaud J-P. A new ant-butterfly symbiosis in the forest canopy fills an evolutionary gap. Sci Rep. 2021;11(1):20770. doi: 10.1038/s41598-021-00274-x 34675260 PMC8531015

[136] Godfray HCJ. Parasitoids: behavioral and evolutionary ecology. Princeton: Princeton University Press; 1994.

