# Supplementary material for: Ant parasitoidism in checkered beetles: Phyllobaenus obscurus developing inside intact cocoons of two species of the Ectatomma ruidum species complex
Source: PLoS One. 2026 Mar 20;21(3):e0335300. doi: 10.1371/journal.pone.0335300 (PMC13004353; doi:10.1371/journal.pone.0335300)
Supplement: Attachment — Submitted filename: Ant parasitoidism in checkered beetles.pdf [file pone.0335300.s005.pdf]

PLOS ONE

Ant parasitoidism in checkered beetles: Phyllobaenus obscurus developing inside intact cocoons of two species of the Ectatomma ruidum species complex

--Manuscript Draft--

**Manuscript Number:**	PONE-D-25-54426
**Article Type:**	Research Article
**Full Title:**	Ant parasitoidism in checkered beetles: Phyllobaenus obscurus developing inside intact cocoons of two species of the Ectatomma ruidum species complex
**Short Title:**	A clerid beetle as brood parasitoid of two Ectatomma ant species
**Corresponding Author:**	Jean-Paul Lachaud, PhD<br>El Colegio de la Frontera Sur - Unidad Chetumal<br>Chetumal, Quintana Roo MEXICO
**Keywords:**	Formicidae; Cleridae; Coleoptera; evolution of ant parasitoidism; interactions; myrmecophile; Mexico
**Abstract:**	Known parasitoids of ants include species from several families of flies, wasps, strepsipterans, nematodes, and mites. Curiously, while myrmecophily is heavily biased towards Coleoptera, one of the most diverse and speciose insect orders, no beetles specialized as parasitoids of ants have been recorded, although the parasitoid habit has evolved at least 13 times within this order. Here we report on observations that strongly suggest that a checkered beetle species behaves as a parasitoid of ant brood. A total of 146 colonies or part of colonies of three species of the Ectatomma ruidum species complex (E.ruidum sp. 2, 3, 4) were excavated in several sites along the Pacific coastal plains of Oaxaca, Mexico, during three collecting campaigns (2015-2017). Overall, 11060 adults, 5795 cocoons and 2185 larvae were examined. Upon dissection, four intact cocoons contained ant prepupae/pupae parasitized by characteristic campodeiform beetle larvae (prognathous head, three pairs of segmented legs on thorax, no prolegs, body with sparse but long pubescence), and a fifth cocoon presented a round exit hole. An active, pink-colored larva, emerged from a cocoon in 2015, was reared to the adult stage and could be identified as Phyllobaenus obscurus (Gorham) (Cleridae). Second and third instar larvae were found inside intact cocoons of two species: E.ruidum sp. 3 and sp. 4. The prevalence of parasitism is extremely low: less than 0.6% of cocoons available in each Ectatomma host populations. Predatory during both adult and larval stages, checkered beetles are broadly known as predators of wood-boring and cone-boring beetles, and some species are facultative parasitoids of solitary bees or wasps or, very rarely, specialized in predating social insects. We assert that the novel discovery of clerid-ant brood parasitoidism within the subterranean host colony deviates yet further from any known clerid adaptation to date.
**Order of Authors:**	Gabriela Pérez-Lachaud, PhD
	Chantal Poteaux, PhD
	John M. Leavengood Jr., PhD
	Jean-Paul Lachaud, PhD
**Additional Information:**	
**Question**	**Response**
**Financial Disclosure**<br><br>Enter a financial disclosure statement that describes the sources of funding for the work included in this submission. Review the submission guidelines for detailed requirements. View published research articles from *PLOS ONE* for specific	Yes

examples.<br><br>This statement is required for submission and **will appear in the published article** if the submission is accepted. Please make sure it is accurate.<br><br>**Funded studies**<br>Enter a statement with the following details:<br>• Initials of the authors who received each award<br>• Grant numbers awarded to each author<br>• The full name of each funder<br>• URL of each funder website<br>• Did the sponsors or funders play any role in the study design, data collection and analysis, decision to publish, or preparation of the manuscript?<br><br>Did you receive funding for this work?	
Please add funding details.<br>as follow-up to "**Financial Disclosure**<br><br>Enter a financial disclosure statement that describes the sources of funding for the work included in this submission. Review the submission guidelines for detailed requirements. View published research articles from *PLOS ONE* for specific examples.<br><br>This statement is required for submission and **will appear in the published article** if the submission is accepted. Please make sure it is accurate.<br><br>**Funded studies**<br>Enter a statement with the following details:<br>• Initials of the authors who received each award<br>• Grant numbers awarded to each author<br>• The full name of each funder<br>• URL of each funder website<br>• Did the sponsors or funders play any role in the study design, data collection and analysis, decision to publish, or preparation of the manuscript?	This study was partially funded by the project number M12A01, Ecos-Nord-CONACYT Program granted to CP and J-PL.

Powered by Editorial Manager® and ProduXion Manager® from Aries Systems Corporation

Did you receive funding for this work?"	
Please select the country of your main research funder (please select carefully as in some cases this is used in fee calculation).<br>as follow-up to "**Financial Disclosure**<br><br>Enter a financial disclosure statement that describes the sources of funding for the work included in this submission. Review the submission guidelines for detailed requirements. View published research articles from *PLOS ONE* for specific examples.<br><br>This statement is required for submission and **will appear in the published article** if the submission is accepted. Please make sure it is accurate.<br><br>**Funded studies**<br>Enter a statement with the following details:<br>• Initials of the authors who received each award<br>• Grant numbers awarded to each author<br>• The full name of each funder<br>• URL of each funder website<br>• Did the sponsors or funders play any role in the study design, data collection and analysis, decision to publish, or preparation of the manuscript?<br><br>Did you receive funding for this work?"	MEXICO - MX
**Competing Interests**<br><br>Use the instructions below to enter a competing interest statement for this submission. On behalf of all authors, disclose any competing interests that could be perceived to bias this work—acknowledging all financial support and any other relevant financial or non-financial competing interests.	The authors have declared that no competing interests exist.

This statement is required for submission and **will appear in the published article** if the submission is accepted. Please make sure it is accurate and that any funding sources listed in your Funding Information later in the submission form are also declared in your Financial Disclosure statement.<br><br>View published research articles from *PLOS ONE* for specific examples.<br><br>**NO authors have competing interests**<br><br>Enter: *The authors have declared that no competing interests exist.*<br><br>**Authors with competing interests**<br><br>Enter competing interest details beginning with this statement:<br><br>*I have read the journal's policy and the authors of this manuscript have the following competing interests: [insert competing interests here]*<br><br>* typeset	
**Ethics Statement**<br><br>Enter an ethics statement for this submission. This statement is required if the study involved:<br><br>• Human participants<br>• Human specimens or tissue<br>• Vertebrate animals or cephalopods<br>• Vertebrate embryos or tissues<br>• Field research<br><br>Write "N/A" if the submission does not require an ethics statement.<br><br>General guidance is provided below. Consult the submission guidelines for detailed instructions. **Make sure that all information entered here is included in the Methods section of the manuscript.**	The species used in this study (E. ruidum complex) are not endangered or protected in Mexico, and the collection of live ants complied with the current laws in Mexico.

Powered by Editorial Manager® and ProduXion Manager® from Aries Systems Corporation

**Format for specific study types**<br><br>**Human Subject Research (involving human participants and/or tissue)**<br>• Give the name of the institutional review board or ethics committee that approved the study<br>• Include the approval number and/or a statement indicating approval of this research<br>• Indicate the form of consent obtained (written/oral) or the reason that consent was not obtained (e.g. the data were analyzed anonymously)<br><br>**Animal Research (involving vertebrate animals, embryos or tissues)**<br>• Provide the name of the Institutional Animal Care and Use Committee (IACUC) or other relevant ethics board that reviewed the study protocol, and indicate whether they approved this research or granted a formal waiver of ethical approval<br>• Include an approval number if one was obtained<br>• If the study involved *non-human primates*, add *additional details* about animal welfare and steps taken to ameliorate suffering<br>• If anesthesia, euthanasia, or any kind of animal sacrifice is part of the study, include briefly which substances and/or methods were applied<br><br>**Field Research**<br><br>Include the following details if this study involves the collection of plant, animal, or other materials from a natural setting:<br>• Field permit number<br>• Name of the institution or relevant body that granted permission	
**Data Availability**<br><br>Authors are required to make all data underlying the findings described fully available, without restriction, and from the time of publication. PLOS allows rare exceptions to address legal and ethical concerns. See the PLOS Data Policy and FAQ for detailed information.	Yes - all data are fully available without restriction

A Data Availability Statement describing where the data can be found is required at submission. Your answers to this question constitute the Data Availability Statement and **will be published in the article**, if accepted.<br><br>**Important:** Stating 'data available on request from the author' is not sufficient. If your data are only available upon request, select 'No' for the first question and explain your exceptional situation in the text box.<br><br>Do the authors confirm that all data underlying the findings described in their manuscript are fully available without restriction?	
**Describe where the data may be found in full sentences. If you are copying our sample text, replace any instances of XXX with the appropriate details.**<br><br>• If the data are **held or will be held in a public repository**, include URLs, accession numbers or DOIs. If this information will only be available after acceptance, indicate this by ticking the box below. For example: *All XXX files are available from the XXX database (accession number(s) XXX, XXX.*).<br>• If the data are all contained **within the manuscript and/or Supporting Information files**, enter the following: *All relevant data are within the manuscript and its Supporting Information files.*<br>• If neither of these applies but you are able to provide **details of access elsewhere**, with or without limitations, please do so. For example:<br><br>*Data cannot be shared publicly because of [XXX]. Data are available from the XXX Institutional Data Access / Ethics Committee (contact via XXX) for researchers who meet the criteria for access to confidential data.*<br><br>*The data underlying the results presented in the study are available*	All relevant data are within the paper and its Supporting Information files.

from (include the name of the third party and contact information or URL).<br>• This text is appropriate if the data are owned by a third party and authors do not have permission to share the data.<br><br>* typeset	
Additional data availability information:	

Manuscript Main Text
Click here to access/download;Manuscript;Manuscript Main text.docx

Short title: A clerid beetle as brood parasitoid of two *Ectatomma* ant species

Ant parasitoidism in checkered beetles: *Phyllobaenus obscurus* developing inside intact cocoons of two species of the *Ectatomma ruidum* species complex

Gabriela Pérez-Lachaud[1*], Chantal Poteaux[2], John M. Leavengood Jr.[3], Jean-Paul Lachaud[1*]

[1]Dpto. Conservación de la Biodiversidad, El Colegio de la Frontera Sur, Chetumal, Quintana Roo, Mexico

[2]Laboratory of Comparative and Experimental Ethology, LEEC, Sorbonne Paris Nord University, Villetaneuse, France

[3]United States Department of Agriculture, APHIS, PPQ, Tampa, Florida, USA

*Corresponding authors: igperez@ecosur.mx (GP-L; ORCID: https://orcid.org/0000-0002-8924-1606); jlachaud@ecosur.mx (J-PL; ORCID: https://orcid.org/0000-0002-5082-8987)

Abstract

Known parasitoids of ants include species from several families of flies, wasps, strepsipterans, nematodes, and mites. Curiously, while myrmecophily is heavily biased towards Coleoptera, one of the most diverse and speciose insect orders, no beetles specialized as parasitoids of ants have been recorded, although the parasitoid habit has evolved at least 13 times within this order. Here we report on observations that strongly suggest that a checkered beetle species behaves as a parasitoid of ant brood. A total of 146 colonies or part of colonies of three species of the *Ectatomma ruidum* species complex (*E. ruidum* sp. 2, 3, 4) were excavated in several sites along the Pacific coastal plains of Oaxaca, Mexico, during three collecting campaigns (2015-2017). Overall, 11060 adults, 5795 cocoons and 2185 larvae were examined. Upon dissection, four intact cocoons contained ant prepupae/pupae parasitized by characteristic campodeiform beetle larvae (prognathous head, three pairs of segmented legs on thorax, no prolegs, body with sparse but long pubescence), and a fifth cocoon presented a round exit hole. An active, pink-colored larva, emerged from a cocoon in 2015, was reared to the adult stage and could be identified as *Phyllobaenus obscurus* (Gorham) (Cleridae). Second and third instar larvae were found inside intact cocoons of two species: *E. ruidum* sp. 3 and sp. 4. The prevalence of parasitism is extremely low: less than 0.6% of cocoons available in each *Ectatomma* host populations. Predatory during both adult and larval stages, checkered beetles are broadly known as predators of wood-boring and cone-boring beetles, and some species are facultative parasitoids of solitary bees or wasps or, very rarely, specialized in predating social insects. We assert that the novel discovery of clerid-ant brood parasitoidism within the subterranean host colony deviates yet further from any known clerid adaptation to date.

Key words: Formicidae, Cleridae, evolution of ant parasitoidism, myrmecophile, Mexico

Introduction

Many invertebrates have adopted a predatory or parasitoid life strategy and play an important role in biological control [1–3]. A parasitoid is an organism in which the immature stages develop on or within a single organism (the host), with this feeding activity eventually resulting in the death or sterilization of the host [4,5], whereas adults are free-living. A diverse array of parasitoids specializes in attacking adult ants or their brood. Known ant parasitoids include species of several insect orders and families: flies, twisted-wing parasites, and parasitic wasps [6–11], two families of Nematoda [12], and one mite family [13–15]. Ant parasitoids are highly specialized symbionts that display a variety of morphological, chemical, and behavioral strategies for accessing and successfully parasitizing their hosts [13,16,17]. For example, species in the genera *Elasmosoma* Ruthe and *Neoneurus* Haliday (Hymenoptera, Braconidae) that parasitize adult ants have morphological adaptations for grasping workers and securing oviposition (e.g., tarsal modifications, raptorial legs) and rely on rapidity to oviposit directly into the ant body [18]. Wasps in the Eucharitidae family lay eggs away from their hosts, in or on plant substrate; they possess a very mobile first-instar larva, termed *planidium,* which is responsible for accessing the hosts through phoresis on foraging ants or attached to prey [19–23]. Chemical volatiles of glandular origin used by ants as pheromones are co-opted by ant-decapitating flies (Phoridae) to locate potential hosts [24]. Once in the vicinity, phorid females rely on short-range chemical signals, such as cuticular hydrocarbons, to locate and select potential hosts [25].

According to Eggleton and Belshaw [4,26], the parasitoid habit has evolved independently at least 13 times in Coleoptera. Beetles do not have piercing ovipositors and the poor flying ability of the adult contrasts with the mobility of the larval stages [4,26]. As in the case of eucharitid wasps and Strepsiptera, known species of Coleoptera with a parasitoid lifestyle lay eggs away

from the hosts and possess phoretic first-instar larvae that are responsible for locating and selecting suitable hosts (Meloidae, Ripiphoridae, some Cleridae [4,19]). The phoretic larvae attach to the adults of their hosts either on living plants, dead wood or soil. Most parasitoid coleopteran families appear to have evolved from ancestors associated with dead wood, with some shifting to attacking insects in the soil. Host orders of coleopteran parasitoids include Diplopoda, Thysanura, Blattaria, Thysanoptera, other Coleoptera, Hymenoptera (other than Formicidae), and Lepidoptera [4]. Compared with parasitoid wasps and flies, their host taxonomic diversity is rather low, suggesting that other factors may constrain the host range of coleopteran parasitoids [4]. Contradictorily, evolution of myrmecophily is heavily biased towards the Coleoptera. The nature of colony exploitation by myrmecophilous beetles varies significantly, with species ranging from scavengers and refuse dwellers to tolerated or highly integrated guests [27]. Some adult myrmecophilous beetles, as in the genus *Cremastocheilus* Knoch [28] or in various Paussinae species [29], are predatory on ant brood and exhibit various strategies to locate and exploit their prey. Some species, such as *Myrmedonota xipe* Mathis & Eldredge, can even use specialized parasitoid host location cues to selectively prey on *Azteca sericeasur* Longino workers that have been previously parasitized by phorid flies [30]. However, to the best of our knowledge, no species of beetle has ever been recorded as developing as a parasitoid of adult ants or their brood. Here we report on observations that strongly suggest that a species of Cleridae develops as a parasitoid of ant brood.

Cleridae is a family of about 4710 described species (R. Gerstmeier, personal communication). Predatory during both adult and larval stages, these beetles are broadly known as predators of wood-boring and cone-boring beetles (e.g., bark beetles, longhorn beetles, powderpost beetles) and are often observed in the galleries of their prey [19,31]. In North America, this most accurately applies to the genera *Chariessa* Perty, *Cregya* LeConte,

Cymatodera Gray*, Enoclerus* Gahan*, Madoniella* Pic*, Monophylla* Spinola*, Neorthopleura* Barr*, Pelonium* Spinola*, Priocera* Kirby*, Pyticeroides* Kuwert*, Tarsostenus* Spinola*,* and *Thanasimus* Latreille*,* recorded in forestry studies and occasional anecdotal observations during sampling [31]. However, several clerid genera are known to feed on gall-makers and their parasitoids (e.g., *Cymatodera*, *Neohydnocera* Leavengood, *Phyllobaenus* Dejean, *Placopterus* Wolcott), on pollen as a primary or secondary protein source (some Clerinae, Korynetinae, Epiclininae), on grasshopper egg pods (*Trichodes* Herbst, *Aulicus* Spinola), on non-gallmaking caterpillars (e.g., *Aulicus, Phyllobaenus*), in the nest cells of larval bees and wasps (e.g., *Cymatodera, Lecontella* Wolcott & Chapin, *Phyllobaenus, Placopterus*, *Trichodes*), on insects associated with carrion (*Necrobia* Latreille) and stored food products and their pests (*Necrobia*) [31,32]. Many species exhibit several different larval feeding strategies, which all may appear to be specialized behaviors (see Table 1). For example, the larvae of *Placopterus thoracicus* (Olivier), which is strongly associated with wood-boring beetles, have been found inside insect galls, and the adults have been reared from cells of crabronid and pemphredonid wasps [48]. Similarly, in *Neohydnocera longicollis* (Ziegler), formerly referred to as *Hydnocera longicollis* Ziegler and *Isohydnocera curtipennis* (Newman), adults have been reported emerging from the galls of three different hosts in three different insect orders (Diptera, Hymenoptera and Lepidoptera) [38,45,48,56,57]. On the other hand, various species, mainly from the genus *Trichodes*, develop as cleptoparasitoids (development at the expense of a single host organism by at least partial usurpation of its food supply, resulting in the death of the host [4]), and many other species have been reared from galls or nest cells of Hymenoptera while also exhibiting other predatory behaviors [31,32,68] (see Table 1). In general, however, the natural histories of most species in these genera are poorly documented.

Table 1. Summary of known rearing records among North American Cleridae and larval feeding mode on insects other than wood, stem and twig borers.

Clerid subfamily and tribe	Species	From galls induced by insects	From nests/cells of solitary bees or wasps	From nests/cells of social Hymenoptera	Other insect prey	Larval feeding mode	References
Tillinae: no tribal assignement	*Cymatodera* sp.		Megachilidae<br>Vespidae			Pr	[33,34]
	Cymatodera ovipennis LeConte		Apidae<br>Megachilidae			Pr	[35]
	Cymatodera undulata (Say)	Cynipidae^{Hy}	Sphecidae		Gall inquilines<br>Chalcididae^{Hy}<br>Cynipidae^{H}<br>Eurytomidae^{Hy}<br>Pteromalidae^{Hy}	Pr	[36–39]
	Lecontella brunnea (Spinola)		Unident. bees<br>Megachilidae<br>Sphecidae<br>Vespidae			Pr, Pa?	[40–42]
Clerinae: Clerini	*Aulicus terrestris* Linsley				Acrididae^{Or}	Pr	[43]
	Enoclerus rosmarus (Say)	Unident. insect<br>Tephritidae^{Di}			Gall inquilines	Pr	[44,45]
	Enoclerus sp.				Tortricidae^{Le}	Pr	[46]
	Enoclerus zonatus (Klug)				*Blastobasidae^{Le}<br>*Nitidulidae^{Le}<br>*Prodoxidae^{Le}	Pr	[47]
	Placopterus thoracicus (Olivier)	Cynipidae^{Hy}	Crabronidae<br>Pemphredonidae		Gall inquilines	Pr	[37,48]
Clerinae: Dieropsini	*Trichodes apivorus* Germar**		Unident. bees<br>Unident. wasps			Pr	[49,50]
	Trichodes bibalteatus LeConte		Megachilidae			Pr	[51]
	Trichodes bicinctus Green		Apidae			Pr	[51]
	Trichodes nuttalli (Kirby)**		Unident. bees		Acrididae^{Or}	Pr	[50,51]

			Unident. wasps				
	Trichodes oregonensis Barr				AcrididaeOr		[51]
	Trichodes oresterus Wolcott		Megachilidae				[51]
	Trichodes ornatus bonnevillensis Foster		Megachilidae<br><br>Vespidae			Pr	[33,34,51]
	Trichodes ornatus douglasianus White		Apidae<br><br>Megachilidae<br><br>Vespidae			Pr, Pa?	[51–54]
	Trichodes ornatus hartwegianus White		Megachilidae			Pr	[51]
	Trichodes ornatus ornatus Say		Unident. bees<br><br>Unident. wasps<br><br>Megachilidae		Post-copulatory cannibalism	Pr	[50,51,54]
	Trichodes ornatus tenuosus Foster		Megachilidae<br><br>Sphecidae				[34,51,55]
	Trichodes peninsularis horni Wolcott & Chapin		Megachilidae			Pr	[39,51]
	Trichodes simulator Horn		Apidae<br><br>Megachilidae			Pr, Pa?	[51]
Clerinae: Hydnocerini	*Neohydnocera longicollis* (Ziegler)	TephritidaeDi<br><br>TenthredinidaeHy<br>GelechiidaeLe			Gall inquilines	Pr, Pa?	[38,45,56,57]
	Neohydnocera tabida (LeConte)	TephritidaeDi			Gall inquilines	Pr	[58]
	Phyllobaenus atriplexus Foster	CecidomyiidaeDi			Gall inquilines<br><br>ChalcididaeHy<br>EulophidaeHy<br><br>EupelmidaeHy<br>EurytomidaeHy<br>PlatygastridaeHy<br>PteromalidaeHy<br>TorymidaeHy	Pr	[59–61]
	Phyllobaenus caeruleipennis (Wolcott)	TephritidaeDi			Gall inquilines	Pr	[62]
	Phyllobaenus dubius (Wolcott)				CephidaeHy	Pr	[63]

	Phyllobaenus humeralis (Say)	Tephritidae^{Di}			Gall inquilines	Pr	[45]
	Phyllobaenus knausii (Wickham)	Unident. insect			Gall inquilines	Pr	[57]
	Phyllobaenus maritimus Wolcott				Tortricidae^{Le}	Pr	[46]
	***Phyllobaenus obscurus* (Gorham)**			**Formicidae^{Hy}**		**Pa**	**This study**
	Phyllobaenus pubescens (LeConte)	Unident. insect	Megachilidae		Gall inquilines<br>Braconidae^{Hy}<br>Noctuidae^{Le}	Pr	[44,57,64–66]
	Phyllobaenus scaber (LeConte)	Gelechiidae^{Le}			Gall inquilines<br>Aphididae^{He}<br>Braconidae^{Hy}<br>Eurytomidae^{Hy}<br>Tortricidae^{Le}<br>Psocidae^{Ps}<br>Unident. spiders	Pr	[67,68]
	Phyllobaenus unifasciatus (Say)	Cynipidae^{Hy}<br>Unident. insect			Gall inquilines	Pr	[37,57]
	Phyllobaenus subaeneus (Spinola)				Tortricidae^{Le}	Pr	[46]
	Phyllobaenus unifasciatus (Say)	Cynipidae^{Hy}<br>Unident. insect			Gall inquilines	Pr	[37,57]
	Phyllobaenus verticalis (Say)	Cynipidae^{Hy}			Gall inquilines	Pr	[37,38,69]

*: Putative prey attacked by larvae.

**: Questionable information. According to Foster (1976), the references that identify *T. apivorus* as a predator in bee and wasp nests "*were inferred apparently from the specific name or from what was known of* Trichodes *biology in genera*l". However, there are no reliable reports of bees or wasps serving as larval hosts for *T. apivorus* and the same seems true for *T. nuttalli* for wich the only known larval host is the acridid Orthoptera, *Chloealtis conspersa* (Harris). See text.

Di: Diptera; He: Hemiptera; Hy: Hymenoptera; Le: Lepidoptera; Or: Orthoptera; Pa: Parasitoid; Pa?: Facutative parasitoid; Pr: Predator; Ps: Psocoptera; Unident: unidentified.

In Europe, the association between *Trichodes umbellatarum* Olivier and the larvae of various bee species borders parasitoidism and predation [70,71], some larvae developing upon a single host as facultative parasitoids while others need several hosts acting as predators. Similar shifts between predation and facultative parasitoidism have also been reported in the New World for some species such as *Lecontella brunnea* (Spinola), *Trichodes ornatus douglasianus* White, *T. simulator* Horn, and *N. longicollis* [42,51,52,57] (Table 1). However, no clerid species associated with hymenopterans can compare to the unique case of *Tarsobaenus letourneauae* Leavengood, Pinkerton & Rifkind and *T. piper* Leavengood, Pinkerton & Rifkind which exhibit a remarkable symbiotic relationship with ants and their host plants [72]. Larvae of both *Tarsobaenus* species have been reported inhabiting the petiole chambers of the Piperaceae *Piper cenocladum* Casimir de Candolle, and *P. obliquum* Ruiz & Pavón, respectively, which host the myrmicine ant *Pheidole bicornis* Forel, and feeding either on the ant brood when ants are present or on the plant-produced food bodies when ants are absent; remarkably, translocation experiments showed that, in the absence of ants, the clerid larva induced food-body production too [73]. As intriguing a case this parasitism of an ant-plant mutualism presents, we assert that the novel discovery of clerid-ant brood parasitism within *Ectatomma* colonies is without precedent among all previously known clerid adaptations to date. Here we report on the first recorded instance of a checkered beetle developing as a parasitoid of the ant brood which also represents the first primary parasitoid of ants from the order Coleoptera. We also summarize all known rearing records among North American Cleridae and conclude by discussing the evolutionary transitions and diversification of larval feeding habits within Cleridae.

Material and methods

The ant hosts

The neotropical ant *Ectatomma ruidum* (Roger) has historically been considered as a widely distributed ant taxon throughout the Neotropics, from Mexico to Brazil [74–77], readily visually recognized. These ants have been subject of intense investigation into colony organization, communication, etc., serving as a species model system for several studies including research on intraspecific cleptobiosis, a very rare behavioral trait [78–80]. Recent studies have revealed that this taxon represents indeed a species complex [77,81–84] including at least 6 putative species, which can be separated based on molecular, chemical and acoustic signatures, and on behavioral traits, but with a considerably conserved morphology which has prevented species description. Two species have a wide distribution, from Mexico to northern Argentina (*E. ruidum* sp. 1 and *E. ruidum* sp. 2), and four other species are apparently restricted to few localities in southern Mexico, along the coast of Oaxaca [83–84].

These ants occur in a wide range of habitats in the Neotropics, including plantations and conserved forests, from sea level up to 1600 m [85,86]. Colonies of these species are monodomous with one single exception [87]. Typically, *E. ruidum* nests have a single entrance about 3–4 mm wide dug in the ground [88,89]. In Chiapas, southern Mexico, colonies are made up of 50 to 200 individuals [90] and colony density can be very high locally (up to 11,200 nests per hectare [91]). These ants have a very generalist diet, and their foraging activity is mainly diurnal [89]. In general, colonies are monogynous, but in some regions they are facultatively polygynous with size-dimorphic queens (macro and microgynes) [92,93]. The variability in such traits has long suggested the existence of a complex of species.

Study site and ant sampling

Beetle larvae were collected along with the ants as part of a larger project aimed at characterizing/unveiling cryptic species in the *E. ruidum* complex and assessing the diversity of some of their already recorded parasitoids (parasitic Hymenoptera, Diptera, and Nematodes, see [94]). Colonies or part of colonies were collected in several sites during three collecting campaigns (November 2015, October 2016 and October/November 2017) in the Pacific Coastal Plain of Oaxaca, Mexico (Fig 1, Table 2). Climate is typical of the Pacific watershed lowlands in southern Mexico, i.e., warm sub-humid with precipitation concentrated in the summer months (Aw0 [95]); mean annual temperature is 22-28 °C; mean annual precipitation is ca. 900 mm. The dominant plant communities in the lowlands (50–450 m asl) are classified as seasonally dry tropical forest [96].

Table 2. Summary of the collection sites in the Oaxaca coast (Mexico) and number of colonies of the different *Ectatomma ruidum* species sampled during 2015-2017.

Ectatomma ruidum species *sensu* Meza-Lázaro et al. [82]	Site	GPS coordinates	Altitud (m asl)	Nb. of sampled colonies (colonies with cocoons)		
				2015	2016	2017
E. ruidum sp. 2	Bajos de Coyula	15°43′58.8" N, 96°17′24" W	125	5 (4)	28 (14)	-
E. ruidum sp. 3	Yerba Santa	15°56′28" N, 97°04′27" W	156	4 (4)	24 (18)	-
E. ruidum sp. 4	Piedras Negras	15°43′49.7" N, 96°40′21.7" W	18	-	22 (13)	-
E. ruidum sp. 4	El Zapotal (Mazunte)	15°41′7.4" N, 96°33′42.5" W	42	4 (4)	22(9)	15 (15)
E. ruidum sp. 4	Puente Cuatode	15°43’55.2” N, 96°0’32.4” W	89	-	22 (17)	-
				13 (12)	118 (71)	15 (15)

Fig 1. Location of the study sites in the coastal plain of Oaxaca, Mexico. A) Ranchería Yerba Santa; B) Piedras Negras; C) El Zapotal (Mazunte); D) Puente Cuatode; E) Bajos de Coyula. Map credits: Holger Weissenberger.

At each site, the entrances of ant nests were detected using cookies as bait and following foraging ants back to their nest. Colonies were excavated and all the individuals present in the nest chambers were collected in plastic bags and transported to the field lab for initial revision. Cocoons of each colony were kept in Petri dishes for a couple of weeks to wait for parasitoid emergence. The rest of the material was preserved in 96° alcohol and revised and counted later under a stereomicroscope. The larvae were thoroughly examined for the presence of any *planidium* or evidence of parasitoid attack (presence of scars evidencing a previous unsuccessful attack or signs of endoparasite presence, see [9,11]). The cocoons were carefully dissected and their contents examined. The identity, number and developmental stage of the parasitoids were recorded. Adult ants were also closely examined for the presence of potential ecto- or endoparasites (e.g., phorid flies, strepsipterans, mites, nematodes, or *planidia*) attached to their body.

Parasitoid identification

The initial assignment of wasps was performed using morphological characters and available keys [97]. Beetles were identified by one of us (JML). Voucher specimens of ants and parasitoids were deposited in the Formicidae and Arthropoda collections of El Colegio de la Frontera Sur-Chetumal (ECO-CH-F and ECO-CH-AR, respectively). Field sampling complied with the current laws of Mexico (collection permit FAUT-0277 from Secretaría del Medio Ambiente y Recursos Naturales - Dirección General de Vida Silvestre granted to GP-L).

Results

A total of 146 colonies or part of colonies of three species of the *Ectatomma ruidum* complex (*E. ruidum* sp. 2, sp. 3, and sp. 4 *sensu* Meza-Lázaro et al. [82]) were sampled. Cocoons and/or larvae were present in a total of 98 excavated colonies or part of colonies (Tables 2, 3, Table S1). Overall, 11060 adults, 5795 cocoons and 2185 larvae were examined.

Table 3. Infestation rate of *Ectatomma ruidum* spp. cocoons by *Phyllobaenus obscurus*.

Year of collection	Species	Locality	Nb. of colonies with cocoons	Total nb. of cocoons	Total nb. of larvae	% of cocoon infestation (exact proportion)
2015	*E. ruidum* sp. 2	Bajos de Coyula	4	111	2	0
	E. ruidum sp. 3	Yerba Santa	4	172	72	0.6 (1/172)
	E. ruidum sp. 4	El Zapotal	4	233	87	0
2016	*E. ruidum* sp. 2	Bajos de Coyula	14	487	74	0
	E. ruidum sp. 3	Yerba Santa	18	1285	794	0
	E. ruidum sp. 4	Puente Cuatode	17	1229	371	0.3 (4/1229)
		El Zapotal	9	717	116	0
		Piedras Negras	13	490	191	0
2017	*E. ruidum* sp. 4	El Zapotal	15	1071	478	0
Total			98	5795	2185	< 0.1 (5/5795)

Workers were parasitized by nematodes (36 cases). The larvae were not parasitized, whilst at dissection eight cocoons were found parasitized by eucharitid wasps belonging to an undetermined species from the *Kapala* Cameron clade, and five cocoons were attacked by beetle larvae (Table 3). In 2015, 12 of the 13 colonies excavated had cocoons. From a total of 516 cocoons maintained in observation, a single, pink-colored larva emerged 10 days later (December 2) from cocoons of a colony of *E. ruidum* sp. 3 collected at Ranchería Yerba Santa (representing

0.6 % of cocoons of this species, Fig 2A, Video S1). The cocoon from which the larva had emerged showed signs of a single round exit hole. When it was dissected, the ant host pupa was found beheaded and only its empty exoskeleton remained (Fig 2B). The larva was isolated in a glass vial along with some fabric to help pupation. An adult belonging to a species of Cleridae (Insecta: Coleoptera) was reared in early January and could be identified as a specimen of *Phyllobaenus obscurus* (Gorham) (Cleridae: Clerinae: Hydnocerini) (Fig 2C). During the 2016 campaign, five sites and 118 colonies were sampled (71 with cocoons). Four cocoons from *E. ruidum* sp. 4 collected at Puente Cuatode, were parasitized by beetle larvae or presented signs of attack (Table 3). One first- and two second-instar larvae were obtained (Figs 3A–3C), each one found inside an intact cocoon on an ant pupa. Another cocoon presented a round hole, suggesting a larva had already left the host to pupate elsewhere (Fig 3D) and the remains of the host pupa were found inside. During 2017 only one site was sampled (El Zapotal, Mazunte; 15 colonies of *E. ruidum* sp. 4) due to instable sociopolitical conditions and insecurity in the region. No beetle or parasitoid wasp was found during this collecting campaign. Whatever the campaign, eggs of the Cleridae were never obtained.

Fig 2. *Phyllobaenus obscurus* (Coleoptera: Cleridae). A) a third-instar larva that has just emerged from the host cocoon; **B)** remains of the *E. ruidum* sp. 3 pupa from which the mature beetle larva has emerged (the dissected cocoon can be observed); C) *Phyllobaenus obscurus* adult reared from the larva that parasitized the pupa in (B). Photo credits: (A-B) Gabriela Pérez-Lachaud, (C) Humberto Bahena-Basave.

Fig 3. *Phyllobaenus obscurus* larvae and remains of the host within the host cocoon. A) Second-instar *P. obscurus* larva, dorsal view. B) Same, ventral view. C) Dorsal view of the head of a second-instar larva, low vacuum SEM photography. D) An *E. ruidum* sp. 4 cocoon showing an exit hole after the departure of a *P. obscurus* larva; the ant pupa remains are visible, the white

arrow pointing at a leg. Photo credits: (A,B,D) Humberto Bahena-Basave; (C) Gabriela Pérez-Lachaud and Manuel Elías-Gutiérrez.

Discussion

The genus *Phyllobaenus*, the second largest Hydnocerini genus after *Callimerus* Gorham, comprises 114 described species [98–101]. Hydnocerini are considered general ant mimics, due either to their wasp-waisting color patterns, their elongate body shape (also sometimes creating a physical "waisting" effect), or their very movement patterns [102–104]. For example, *Neohydnocera aegra* (Newman) strikingly resembles a species of *Pseudomyrmex* Lund and has been found abundantly among these ants [105], and *Phyllobaenus cryptocerinus* (Gorham) is a reported mimic of *Cephalotes* Latreille ants (= *Cryptocerus* Latreille) [102].

Like many of its congeners, *P. obscurus* is an active clerid with a highly variable color pattern and body size (2-5 mm). This intraspecific plasticity can make species determination difficult as there are many similar Hydnocerini species [100]. This species is known from Mexico, Guatemala, Costa Rica, and Panama [106–109]. Araujo et al. [110] included Brazil based on an unvouchered record with no data nor depository presented from a personal database (see "Unverified Distributions" in [111]). Prior to this study it was known only from Durango, Morelos and Tamaulipas in Mexico [112,113]. The data presented herein for Oaxaca and an unpublished record from Chiapas ([Verbatim label data] 3000', "El Chorreadero" by Hwy. 190, 1.25km E Tuxtla Gutierrez, under debris, gravel bank of stream in deep ravine, 25-XII-1972, H. Frania, FSCA, Florida State Collection of Arthropods) are new state records for Mexico. Despite the broad distribution of this rather common species, nothing was known about its natural history prior to our findings, and the larva and larval feeding habits were unknown.

Most specimens of *P. obscurus* have been collected by beating the foliage of live trees and occasionally shrubs (JML personal observations; accounts from numerous beetle collectors). Specimen label data indicate few specimens collected with other methods (e.g., lights, fogging, Malaise trap) (Leavengood, unpublished data). Notably, label data for the Chiapas new state record for Mexico (see above) indicate an adult was caught on the ground and under debris, which is most unusual. In the Western Hemisphere, hydnocerines are not observed on the ground, but usually high in foliage (e.g., [104]) and there are no specimen records of *Phyllobaenus* collected in pitfall traps. Author JML has been presented two separate accounts of a *Phyllobaenus* specimen emerging from an ant hill in the ground. But no specimens were produced nor identifications made. So, with only one vouchered record (Chiapas, FSCA), two unvouchered events (ant hill emergence observations), and the five brood parasitoid observations noted herein (recorded in different years, at two different places, and concerning two different ant species), any record of *Phyllobaenus* at ground level (or below) is highly unusual, yet all but one such record are specifically associated with ants. Furthermore, it should be noted that our records concern clerid larvae which were sampled inside ant nests, in deep chambers.

Myrmecophilous taxa are not scattered randomly across the arthropod tree of life. Rather, their evolution is strongly biased to certain groups and, among insects, towards the Coleoptera which "*contains a large pool of lineages with the potential for transitioning to myrmecophily*" (Parker 2016). At least 33 of the beetle families, representing approximately one-fifth, have thus far been documented to include one or more taxa that exhibits close association with ant colonies [27]. Many of the most familiar beetle families contain at least one lineage that inhabits ant colonies, including the mainly aquatic beetle family Hydrophilidae [114]. Indeed, at least two species of *Sphaerocetum* Fikáček have been collected in mixed nests of *Camponotus* Mayr and *Crematogaster* Lund, and a larva of *S. arboreum* Fikáček et al. was collected in an ant nest along

with the adults, implying the entire life cycle may take place inside the ant nest, suggesting that these beetles likely prey on ant larvae [114].

Ants and their nests are reliable, fixed resources in space, and potential targets that are available all year round [115]. In addition, as suggested by Parker [27], several ecological constraints and phenotypic attributes may predispose some taxa to undergo the evolutionary transition from free-living to myrmecophily which, in combination, could explain the myrmecophile beetle bias: i) the huge species richness of Coleoptera, ii) their ecological predisposition to encountering and exploring ant colonies, and iii) their possession of a major protective preadaptation in the form of elytra. Additionally, the size of the myrmecophile relative to the ant host species (small myrmecophile species/large host), a limuloid body form, and other protective morpho-anatomical adaptations (i.e., pedofossae) are traits that facilitate the integration of some intruders into colony life (see [13,116–118]).

Whether *P. obscurus* is a facultative or obligate parasitoid of the ant brood remains to be elucidated. Our finding of an unusual feeding mode for a checkered beetle is surprising but not entirely unexpected as other checkered beetles belonging to different tribes border the line between predator and parasitoid [42,51,52,57,70,71], likely depending on the relative size of their prey/host. It suggests that the parasitoid lifestyle may have evolved several times within the Cleridae. Notably, our record is also the first evidence of beetles parasitizing successfully the immature stages of ants within their subterranean nests, suggesting a major evolutionary shift in the biology of Hydnocerini. Our results further support a previously stated hypothesis about the evolution of ant parasitoidism in clades with predatory habits. As suggested for other unusual ant parasitoids such as the microdontine syrphid fly *Hypselosyrphus trigonus* Hull [119], the only parasitoid species among this clade of predators, it is likely that parasitoidism in *P. obscurus* evolved from a predatory ancestor. According to Godfray [120], the transition from a predatory to

a parasitoid lifestyle may be straightforward since a predator that requires only a single prey item for its development is, by definition, a parasitoid.

There are still some gaps in knowledge regarding the biology of this clerid. Like other ant parasitoids such as eucharitid wasps of the genera *Kapala*, *Isomerala* Shipp, *Obeza* Heraty, or *Pseudochalcura* Ashmead [8], *P. obscurus* develops at the expense of host larvae/pupae under the protection of a cocoon, avoiding deadly interactions with their host ants. But, as our observations show, when larval development is completed, the larva exits the host cocoon to pupate. Clerids are known to commonly pupate near their prey (e.g., in galleries of their prey, or tree crevices nearby or inside the host pupal cell [57,58]), but the site of pupation of *P. obscurus* remains to be uncovered. Its high mobility and small size (only 2-5 mm for the adult) relative to the *Ectatomma* hosts (8-9 mm) may help avoiding ant aggressiveness. Infiltration into the ant nest is probably by phoresis of the mobile first-instar larva (*triungulin*) as occurs in other ant parasitoids which lay eggs away from the host [7,20,22]. Escape behaviors are an important part of myrmecophile strategies to adapt to their hosts, and the mobility of the adult may be responsible for leaving the natal nest unnoticed and the protective function of the elytra for coping with the host aggressiveness.

Although some checkered beetle species are considered of importance as potential biological control agents, there are very few rearing records for Cleridae in general, and data on their natural life history are scarce (Table 1). However, results of laboratory and field studies underscore the plasticity and adaptability in the food habits of Cleridae (e.g., [54] and references in Table 1). This deviating lineage (from predation) of foraging behavior towards parasitoidism reported here is likely to be a confirmation of the diversified feeding traits within the Cleridae.

Acknowledgements

We are grateful to Ruby Meza-Lázaro, Alejandro Zaldívar-Riverón (UNAM, Mexico) and Carlos Santamaría (Universidad del Valle, Cali, Colombia) for their help during the 2016 field work; Humberto Bahena-Basave (Ecosur) for help with insect pictures, Holger Weissenberger (Ecosur) for help with the map, Manuel Elías-Gutiérrez (Ecosur) for help with SEM photography, and Roland Gerstmeier (Zoologische Staatssammlung München, Germany) for an up to date estimate of world clerid species.

References

1. Waage JK, Hassell MP. Parasitoids as biological control agents – a fundamental approach. Parasitology. 1982;84(4): 241–268. https://doi.org/10.1017/S003118200005366X
2. Greany PD, Vinson SB, Lewis WJ. Insect parasitoids: Finding new opportunities for biological control. BioScience 1984;34(11): 690–696. https://doi.org/10.2307/1309661
3. Koller J, Sutter L, Gonthier J, Collatz J, Norgrove L. Entomopathogens and parasitoids allied in biocontrol: a systematic review. Pathogens. 2023;12: 957. https://doi.org/10.3390/pathogens12070957
4. Eggleton P, Belshaw R. Insect parasitoids: an evolutionary overview. Phil Trans R Soc Lond B 1992;337(1279): 1–20. https://doi.org/10.1098/rstb.1992.0079
5. Quicke DLJ. Parasitic Wasps. London: Chapman & Hall; 1997.
6. Feener DH Jr, Brown BV. Diptera as parasitoids. Annu Rev Entomol 1997;42: 73–97. https://doi.org/10.1146/annurev.ento.42.1.73

7. Kathirithamby J. Host-parasitoid associations in Strepsiptera. Annu Rev Entomol 2009;54: 227–249. https://doi.org/10.1146/annurev.ento.54.110807.090525
8. Lachaud J-P, Pérez-Lachaud G. Diversity of species and behavior of hymenopteran parasitoids of ants: a review. Psyche. 2012;2012: 134746. https://doi.org/10.1155/2012/134746
9. Pérez-Lachaud G, Lachaud J-P. Arboreal ant colonies as 'hot-points' of cryptic diversity for myrmecophiles: the weaver ant *Camponotus* sp. aff. *textor* and its interaction network with its associates. PLoS ONE. 2014;9(6): e100155. https://doi.org/10.1371/journal.pone.0100155
10. González CT, Wcislo WT, Cambra R, Wheeler TA, Fernández-Marín H. A new ectoparasitoid species of *Pseudogaurax* Malloch, 1915 (Diptera: Chloropidae), attacking the fungus-growing ant, *Apterostigma dentigerum* Wheeler, 1925 (Hymenoptera: Formicidae). Ann Entomol Soc Am. 2016;109(4): 639–645. https://doi.org/10.1093/aesa/saw023
11. Pérez-Lachaud G, Jahyny BJB, Stahls G, Rotheray G, Delabie JHC, Lachaud J-P. Rediscovery and reclassification of the dipteran taxon *Nothomicrodon* Wheeler, an exclusive endoparasitoid of gyne ant larvae. Sci Rep. 2017;7: 45530. https://doi.org/10.1038/srep45530
12. Poinar G Jr. Nematode parasites and associates of ants: past and present. Psyche. 2012;2012: 192017. https://doi.org/10.1155/2012/192017
13. Lachaud J-P, Klompen H, Pérez-Lachaud G. *Macrodinychus* mites as parasitoids of invasive ants: an overlooked parasitic association. Sci Rep. 2016;6: 29995. https://doi.org/10.1038/srep29995

14. Brückner A, Klompen H, Bruce AI, Hashim R, von Beeren C. Infection of army ant pupae by two new parasitoid mites (Mesostigmata: Uropodina). PeerJ. 2017;5: e3870. https://doi.org/10.7717/peerj.3870
15. Pérez-Lachaud G, Klompen H, Poteaux C, Santamaría C, Armbrecht I, Beugnon G, Lachaud J-P. Context dependent life-history shift in *Macrodinychus sellnicki* mites attacking a native ant host in Colombia. Sci Rep. 2019;9: 8394. https://doi.org/10.1038/s41598-019-44791-2
16. Vander Meer RK, Jouvenaz DP, Wojcik DP. Chemical mimicry in a parasitoid (Hymenoptera: Eucharitidae) of fire ants (Hymenoptera: Formicidae). J Chem Ecol. 1989;15(8): 2247–2261. https://doi.org/10.1007/BF01014113
17. Pérez-Lachaud G, Bartolo-Reyes JC, Quiroa-Montalván CM, Cruz-López L, Lenoir A, Lachaud J-P. How to escape from the host nest: Imperfect chemical mimicry in eucharitid parasitoids and exploitation of the ants' hygienic behavior. J Insect Physiol. 2015;75: 63–72. http://dx.doi.org/10.1016/j.jinsphys.2015.03.003
18. Gómez Durán JM, van Achterberg C. Oviposition behaviour of four ant parasitoids (Hymenoptera, Braconidae, Euphorinae, Neoneurini and Ichneumonidae, Hybrizontinae), with the description of three new European species. ZooKeys. 2011;125: 59–106. https://doi.org/10.3897/zookeys.125.1754
19. Clausen CP. Entomophagous insects. 1st ed. New York: McGraw-Hill; 1940.
20. Clausen CP. The oviposition habits of the Eucharidae (Hymenoptera). J Wash Acad Sci. 1940;30(12): 504–516.
21. Heraty JM. A revision of the genera of Eucharitidae (Hymenoptera: Chalcidoidea) of the world. Mem Amer Ent Inst. 2002;68: 1–359.

22. Carey B, Visscher K, Heraty J. Nectary use for gaining access to an ant host by the parasitoid *Orasema simulatrix* (Hymenoptera, Eucharitidae). J Hym Res. 2012;27: 47–65. https://doi.org/10.3897/jhr.27.3067

23. Herreid JS, Heraty JM. Hitchhikers at the dinner table: a revisionary study of a group of ant parasitoids (Hymenoptera: Eucharitidae) specializing in the use of extrafloral nectaries for host access. Syst Entomol 2017;42(1): 204–229. https://doi.org/10.1111/syen.12206

24. Mathis KA, Philpott SM. Current understanding and future prospects of host selection, acceptance, discrimination, and regulation of phorid fly parasitoids that attack ants. Psyche 2012;2012: 895424 https://doi.org/10.1155/2012/895424

25. Mathis KA, Tsutsui ND. Cuticular hydrocarbon cues are used for host acceptance by *Pseudacteaon* spp. phorid flies that attack *Azteca sericeasur* ants. J Chem Ecol. 2016;42(4): 286–293. https://doi.org/10.1007/s10886-016-0694-y

26. Eggleton P, Belshaw R. Comparisons of dipteran, hymenopteran and coleopteran parasitoid: provisional phylogenetic explanations. Biol J Linn Soc 1993;48(3): 213–226. https://doi.org/10.1006/bijl.1993.1015

27. Parker J. Myrmecophily in beetles (Coleoptera): evolutionary patterns and biological mechanisms. Myrmecol News. 2016;22: 65–108. https://doi.org/10.25849/myrmecol.news_022:065

28. Alpert GD, Richter PO. Notes on the life cycle and myrmecophilous adaptations of *Cremastocheilus armatus* (Coleoptera: Scarabaeidae). Psyche. 1975;82: 283–291. https://doi.org/10.1155/1975/79470

29. Geiselhardt SF, Peschke K, Nagel P. A review of myrmecophily in ant nest beetles (Coleoptera: Carabidae: Paussinae): linking early observations with recent findings. Naturwissenschaften 2007;94(11): 871–894. https://doi.org/10.1007/s00114-007-0271-x

30. Mathis KA, Tsutsui ND. Dead ant walking: a myrmecophilous beetle predator uses parasitoid host location cues to selectively prey on parasitized ants. Proc R Soc B. 2016;283(1836): 20161281. http://dx.doi.org/10.1098/rspb.2016.1281

31. Chapman EG, Leavengood JM Jr, Dupuis JR. The Cleridae and Thanerocleridae of eastern North America, with illustrated keys, updated distributions, and special emphasis on the Kentucky fauna. Zootaxa 2025;5639(1): 1–88. https://doi.org/10.11646/zootaxa.5639.1.1

32. Leavengood JM Jr. The checkered beetles (Coleoptera: Cleridae) of Florida. M.Sc. Thesis, University of Florida. 2008. Available from : http://etd.fcla.edu/UF/UFE0023794/leavengood_j.pdf

33. Parker FD. Nests and nest associates of two "red" *Odynerus* wasps (Hymenoptera: Eumenidae). J Kansas Entomol Soc. 1984;57(3): 521–525. http://www.jstor.org/stable/25084551

34. Parker FD, Bohart RM. Host parasite associations in some twig-nesting Hymenoptera from Western North America. Part II. Pan-Pac Entomol. 1968;44(1): 1–6.

35. MacSwain JW. Notes on the habits of the predator *Cymatodera ovipennis* Say with a description of the pupa. Pan-Pac Entomol. 1945;21(3): 97–100.

36. Balduf WV. On the habits and development of a checkered beetle (*Cymatodera undulata* Say, Cleridae). Trans Am Entomol Soc. 1926;52(1): 29–37. https://www.jstor.org/stable/25077149

37. Eliason EA, Potter DA. Biology of *Callirhytis cornigera* (Hymenoptera: Cynipidae) and the arthropod community inhabiting its galls. Environ Entomol. 2000; 29(3): 551–559. https://doi.org/10.1603/0046-225X-29.3.551

38. Gosling DCL. An annotated list of the checkered beetles (Coleoptera: Cleridae) of Michigan. Gt Lakes Entomol. 1980;13(2): 65–76. https://doi.org/10.22543/0090-0222.2551

39. Krombein KV. Trap-Nesting Wasps and Bees: Life Histories, Nests, and Associates. Washington, D.C., Smithsonian Press. 1967.

40. Bitner RM. Predation by the larvae of *Lecontella cancellata* (LeConte) (Coleoptera: Cleridae) on seven species of aculeate Hymenoptera. Ent News. 1972;83(1): 23–26.

41. Knull JN. Notes on Coleoptera.—No.3. Ent News 1932;43(2): 42–45.

42. Rau P. A note on *Lecontella cancellata* Lec. (Coleoptera: Cleridae) in cells of the mud-daubing wasp. Ent News. 1944;55(8): 197.

43. Linsley EG. Studies in the genus *Aulicus* Spinola (Coleoptera-Cleridae). Univ Calif Publ Ent. 1936;6: 249–262.

44. Mawdsley JR. Ecological notes on species of Cleridae (Insecta: Coleoptera) associated with the prairie flora of Central North America. Gt Lakes Entomol. 2002;35(1): 15–22. https://doi.org/10.22543/0090-0222.2056

45. Price JR. Post-release assessment of classical biological control of Canada thistle (*Cirsium arvense*) in the western United States. M.Sc. Thesis, University of Idaho. 2014. Available from https://objects.lib.uidaho.edu/etd/pdf/Price_idaho_0089M_10185.pdf

46. Eikenbary RD, Fox RC. Arthropod predators of the Nantucket pine tip moth, *Rhyacionia frustrana*. Ann Entomol Soc Am. 1968;61(5): 1218–1221. https://doi.org/10.1093/aesa/61.5.1218

47. Powell JA. Prolonged diapause in *Enoclerus zonatus* (Cleridae). Coleopt Bull. 1975;29(1): 44. http://www.jstor.org/stable/3999837

48. Foster DE, Barr WF. Notes on the distribution and bionomics of some North American Cleridae. J Kansas Entomol Soc 1972;45(1): 122–125 http://www.jstor.org/stable/25082471

49. Balduf WV. The bionomics of entomophagous Coleoptera. St Louis. 1935.

50. Böving AG, Champlain AB. Larvae of North American beetles of the family Cleridae. Proc US Natl Mus. 1920; 57(2323): 575–649. https://doi.org/10.5479/si.00963801.57-2323.575
51. Foster DE. Revision of North American Trichodes (Herbst) (Coleoptera: Cleridae). Spec Publ Mus Texas Tech Univ1976; 11: 1–86.
52. Essig EO. *Trichodes ornatus* Say. J Econ Entomol. 1934;27: 724.
53. Linsley EG. Notes on the habits of some beetles from the vicinity of Yosemite National Park. Bull So Calif Acad Sci. 1942;41: 164–166.
54. Linsley EG, MacSwain JW. Observations on the life history of *Trichodes ornatus* (Coleoptera, Cleridae), a larval predator in the nests of bees and wasps. Ann Entomol Soc Am. 1943;36(4): 589–601. https://doi.org/10.1093/aesa/36.4.589
55. Davidson A. On the nesting habits of *Anthidium consimile*. Ent News. 1896;7(1): 22–26.
56. Baker CF. Biological notes on some Colorado Coleoptera. Ent News 1895;6(1): 27–29.
57. Sabrosky CW. Notes on the larva and larval habit of *Isohydnocera curtipennis* (Newmn.) (Coleoptera, Cleridae). J Kansas Entomol Soc. 1934;7(2): 65–68. https://www.jstor.org/stable/25081380
58. Swenson SJ, Bell-Clement J, Schroeder S, Prischmann-Voldseth DA. Predatory and parasitic insects associated with *Urophora cardui* L. (Diptera: Tephritidae) galls on Canada thistle, *Cirsium arvense* L. (Asterales, Asteraceae) in North Dakota. Insects. 2022;13: 646. https://doi.org/10.3390/insects13070646
59. Foster DE. A new species of *Phyllobaenus* (Coleoptera: Cleridae). J Kansas Entol Soc. 1981;54(3): 661–664. https://www.jstor.org/stable/25084203
60. Goeden RD, Ricker DW, Müller H. Introduction, recovery, and limited establishment of *Coleophora klimeschiella* (Lepidoptera: Coleophoridae) on Russian thistles, *Salsola*

australis, in Southern California. Environ Entomol. 1987;16(4): 1027–1029. https://doi.org/10.1093/ee/16.4.1027

61. Hawkins BA, Goeden RD. Organization of a parasitoid community associated with a complex of galls on *Atriplex* spp. in southern California. Ecol Entomol. 1984;9(3): 271–292. https://doi.org/10.1111/j.1365-2311.1984.tb00851.x
62. Baine Q, Casares EE, Hughes DWW, Martinson VG, Martinson EO. Arthropod communities associated with gall-inducing *Aciurina bigeloviae* and *Aciurina trixa* (Diptera: Tephritidae) in New Mexico, Ann Entomol Soc Am. 2024;117(2): 107–117. https://doi.org/10.1093/aesa/saad037
63. Morrill WL, Weaver DK, Irish NJ, Barr WF. *Phyllobaenus dubiu*s (Wolcott) (Coleoptera: Cleridae), a new record of a predator of the wheat stem sawfly (Hymenoptera: Cephidae). J Kansas Entomol Soc. 2001;74(3): 181–183. https://www.jstor.org/stable/25086016
64. Farrar RR. A new record of the clerid, *Phyllobaenus pubescens* (LeConte) (Coleoptera), as a predator of *Heliothis zea* (Boddie) (Lepidoptera: Noctuidae) on cotton. Coleopt Bull. 1985;39(1): 33–34. https://www.jstor.org/stable/4008186
65. Pierce WD, Cushman RA, Hood CE, Hunter WD. The insect enemies of the cotton boll weevil. USDA Bur Entomol Bull. 1912;100: 1–99.
66. Rogers CE, Perkins WD, Seiler GT. Bionomics of *Stibadium spumosum* (Lepidoptera: Noctuidae), a pest of sunflower in the southern plains. Environ Entomol 1986;15(6): 1275–1278. https://doi.org/10.1093/ee/15.6.1275
67. Coquillet DW. Notes on the habits of some California Coleoptera. Insect Life. 1912;4: 260–262.
68. Tilden JW. Notes on the habits of *Phyllobaenus scabra* (Le Conte) (Cleridae). Coleopt Bull. 1950;4(2): 17–19. https://www.jstor.org/stable/3998796

69. Osten Sacken R. On the Cynipidae of North American oaks and their galls. Proc Entomol Soc Phil. 1861;1: 47–72.

70. Cros A. Le *Trichodes umbellatarum* Ol., ses moeurs, son évolution. Bull Mus Natl Hist Nat. 1908;14: 215–221.

71. Cros A. Note sur le *Trichodes umbellatarum* Ol. Bull Soc Hist Nat Afr Nord. 1911;2(7-9): 111–115, 135–140, 142–149.

72. Leavengood JM Jr, Pinkerton MG, Rifkind J. Description of the new genus *Tarsobaenus* and three new species from Costa Rica (Coleoptera: Cleridae: Hydnocerinae). Zootaxa. 2022;5138(2): 167–176. http://doi.org/10.11646/zootaxa.5138.2.4

73. Letourneau DK. Code of ant-plant mutualism broken by parasite. Science 1990;248(4952): 215–217. https://doi.org/10.1126/science.248.4952.215

74. Brown WL Jr. Contributions toward a reclassification of the Formicidae. II. Tribe Ectatommini (Hymenoptera). Bull Mus Comp Zool. 1958; 118(5): 175–362. https://biostor.org/reference/21040

75. Kugler C, Brown WL Jr. Revisionary & other studies on the ant genus *Ectatomma*, including the descriptions of two new species. Search: Agriculture. 1982; 24: 1–8.

76. Nettel-Hernanz A, Lachaud J-P, Fresneau D, Lopez- Muñoz RA, Poteaux C. Biogeography, cryptic diversity, and queen dimorphism evolution of the Neotropical ant genus *Ectatomma* Smith 1858 (Formicidae: Ectatomminae). Org Divers Evol 2015;15(3): 543–553. https://doi.org/10.1007/s13127-015-0215-9

77. Aguilar-Velasco RG, Poteaux C, Meza-Lázaro R, Lachaud J-P, Dubovikoff D, Zaldívar-Riverón A. Uncovering species boundaries in the Neotropical ant complex *Ectatomma ruidum* (Ectatomminae) under the presence of nuclear mitochondrial paralogues. Zool J Linn Soc. 2016;178(2): 226–240. https://doi.org/10.1111/zoj.12407

78. Breed MD, Abel P, Bleuze TJ, Denton SE. Thievery, home ranges, and nestmate recognition in *Ectatomma ruidum*. Oecologia 1990;84(1): 117–121. https://doi.org/10.1007/BF00665604

79. De Carli P, Lachaud J-P, Beugnon G, López-Méndez JA. Études en milieu naturel du comportement de cleptobiose chez la fourmi néotropicale *Ectatomma ruidum* (Hymenoptera, Ponerinae). Actes Coll Insectes Soc 1998;11: 29–32.

80. Guénard B, McGlynn TP. Intraspecific thievery in the ant *Ectatomma ruidum* is mediated by food availability. Biotropica 2013;45(4): 497–502. https://doi.org/10.1111/btp.12031

81. Meza-Lázaro RN, Poteaux C, Bayona-Vásquez NJ, Branstetter MG, Zaldívar-Riverón A. Extensive mitochondrial heteroplasmy in the neotropical ants of the *Ectatomma ruidum* complex (Formicidae: Ectatomminae). Mitochondrial DNA A. 2018;29(8): 1203–1214. https://doi.org/10.1080/24701 394.2018.1431228

82. Meza-Lázaro RN, Peña-Carrillo KI, Poteaux C, Lorenzi MC, Wetterer JK, Zaldívar-Riverón A. Genome and cuticular hydrocarbon-based species delimitation shed light on potential drivers of speciation in a Neotropical ant species complex. Ecol Evol. 2022;12: e8704. https://doi.org/10.1002/ece3.8704

83. Peña-Carrillo KI, Poteaux C, Leroy C, Meza-Lázaro RN, Lachaud J-P, Zaldívar-Riverón A, Lorenzi MC. Highly divergent cuticular hydrocarbon profiles in the clepotobiotic ants of the *Ectatomma ruidum* species complex. Chemoecology. 2022;31(2): 125–135. https://doi.org/10.1007/s0004 9-020-00334-0

84. Peña-Carrillo KI, Lorenzi MC, Brault M, Devienne P, Lachaud J-P, Pavan G, Poteaux C. A new putative species in the *Ectatomma ruidum* complex (Formicidae: Ectatomminae) produces a species-specific distress call. Bioacoustics. 2022;31(3): 332–347. https://doi.org/10.1080/09524 622.2021.1938226

85. McGlynn TP, Graham R, Wilson J, Emerson J, Jandt JM, Jahren AH. Distinct types of foragers in the ant *Ectatomma ruidum*: typical foragers and furtive thieves. Anim Behav. 2015;109: 243–247. https://doi.org/10.1016/j.anbehav.2015.08.024
86. Lachaud J-P. *Ectatomma*. In: Starr CK, editor. Encyclopedia of Social Insects. Cham: Springer; 2021. pp. 358–365. https://doi.org/10.1007/978-3-319-90306-4_41-1
87. Santamaría C, Armbrecht I, Lachaud J-P. Nest architecture and colony composition in two populations of *Ectatomma ruidum* sp. 2 (*E. ruidum* species complex) in southwestern Colombia. PLoS ONE. 2022;17(2): e0263382. https://doi.org/10.1371/journal.pone.0263382
88. Lachaud J-P, Fresneau D, García-Pérez J. Étude des stratégies d'approvisionnement chez 3 espèces de fourmis ponérines (Hymenoptera, Formicidae). Folia Entomol Mex. 1984;61: 159–177.
89. Lachaud J-P. Foraging activity and diet in some neotropical ponerine ants. I. *Ectatomma ruidum* Roger (Hymenoptera, Formicidae). Folia Entomol Mex. 1990;78: 241–256.
90. Lachaud J-P. Recruitment by selective activation: an archaic type of mass recruitment in a ponerine ant (*Ectatomma ruidum*). Sociobiology. 1985;11(2): 133–142.
91. Schatz B, Lachaud J-P. Effect of high nest density on spatial relationships in two dominant ectatommine ants (Hymenoptera: Formicidae). Sociobiology. 2008;51(3): 623–643.
92. Lachaud J-P, Cadena A, Schatz B, Pérez-Lachaud G, Ibarra-Núñez G. Queen dimorphism and reproductive capacity in the ponerine ant, *Ectatomma ruidum* Roger. Oecologia 1999;120(4): 515–523. https://doi.org/10.1007/s004420050885
93. Lenoir J-C, Lachaud J-P, Nettel A, Fresneau D., Poteaux C. The role of microgynes in the reproductive strategy of the neotropical ant *Ectatomma ruidum*. Naturwissenschaften 2011; 98(4): 347–356. https://doi.org/10.1007/s00114-011-0774-3

94. Lachaud J-P, Pérez-Lachaud G. Ectaheteromorph ants also host highly diverse parasitic communities: a review of parasitoids of the Neotropical genus *Ectatomma*. Insect Soc 2015;62(2): 121–132. https://doi.org/0.1007/s00040-015-0390-x

95. García E. Modificaciones al sistema de clasificación climática de Köppen (para adaptarlo a las condiciones de la república Mexicana). 5th ed. Mexico: Instituto de Geografía. UNAM; 2004.

96. Salas-Morales SH, Meave JA. Elevational patterns in the vascular flora of a highly diverse region in southern Mexico. Plant Ecol. 2012;213(8): 1209–1220. https://doi.org/10.1007/s11258-012-0077-6

97. Silva TSR, Silva-Freitas JM, Schoeninger K. A synopsis of the Brazilian Eucharitidae (Hymenoptera: Chalcidoidea) fauna: an annotated checklist of the family in the country, with a revised key for the New World genera. Zootaxa. 2019;4564(2): 347–366. https://doi.org/10.11646/zootaxa.4564.2.3

98. Leavengood JM Jr. Nomenclatural and distributional notes regarding some North American species of *Phyllobaenus* (Coleoptera: Cleridae: Hydnocerinae). G It Ent 2014;13(59): 471–480.

99. Leavengood JM Jr. *Phyllobaenus thomasi* and *P. turnbowi*, two new species from Mexico and Belize (Coleoptera: Cleridae: Hydnocerinae: Hydnocerini). Insecta Mundi. 2020;0833: 1–6. https://journals.flvc.org/mundi/article/view/127561/127813

100. Leavengood JM Jr. Living a lie: The truth behind *Isohydnocera curtipennis* (Newman) and other historically confused species of *Phyllobaenus* Dejean, with the description of the new genus *Neohydnocera* (Coleoptera: Cleridae: Clerinae: Hydnocerini: Hydnocerina). Insecta Mundi. 2025;1105: 1–17. https://digitalcommons.unl.edu/insectamundi/1613

101. Leavengood JM Jr, Rifkind J. *Phyllobaenus inusitatotibialis*, a new species from southern Arizona (Coleoptera: Cleridae: Hydnocerinae: Hydnocerini). Proc Entomol Soc Wash. 2020;122(3): 673–677. https://doi.org/10.4289/0013-8797.122.3.673

102. Gorham HS. Insecta. Coleoptera. Malacodermata. Cleridae. In: Godman FD, Salvin O, editors. Biologia Centrali-Americana. London: Dulau; 1882;3(2): 113–168.

103. Mawdsley JR. Mimicry in Cleridae (Coleoptera). Coleopt Bull. 1994;48(2): 115–125. https://www.jstor.org/stable/4009121

104. Leavengood JM Jr, Gemmill RM, Raber B, Quinn MA. Notes on the identification, distribution and natural history of *Phyllobaenus corticinus* (Gorham, 1883) (Coleoptera: Cleridae: Hydnocerinae) including the first report from the United States. Coleopt Bull. 2012;66(4): 351–356. https://doi.org/10.1649/072.066.0410

105. Wheeler AG Jr, Stocks IC. *Wolcottia* (= *Isohydnocera*) *aegra* (Coleoptera: Cleridae): association with grasses (Poaceae), native *Spartina* spp. and the introduced *Eragrostis curvula*, and resemblance to co-occurring pseudomyrmecine ants. Fla Entomol. 2009;92(4): 648–652. https://doi.org/10.1653/024.092.0419

106. Gorham HS. Insecta. Coleoptera. Malacodermata. Cleridae. In: Godman FD, Salvin O, editors. Biologia Centrali-Americana. London: Dulau; 1883;3(2): 169–193.

107. Wolcott AB. A review of the Cleridae of Costa Rica. Coleopt Contrib. 1927;1(10):1–104.

108. Leavengood JM Jr, Garner BH. Nomenclatural notes on some checkered beetle (Coleoptera: Cleridae) types of the Natural History Museum, London (BMNH). Zootaxa. 2014;3760(3): 301–335. http://dx.doi.org/10.11646/zootaxa.3760.3.1

109. Toledo-Hernández VH, Rifkind J, Corona-López AM, Flores-Palacios A, Leavengood JM Jr. Faunistic composition of Cleridae (Coleoptera) in El Limón de Cuauchichinola, Morelos, Mexico. Ann Entomol Soc Am. 2015;108(5): 771–776. https://doi.org/10.1093/aesa/sav039

110. Araujo Castillo S, Romero Napoles J, Rifkind J, Flores Dávila M, García Martínez O, Cerna Chávez E. Biodiversidad de la familia Cleridae (Coleoptera: Cleroidea) de México. Folia Entomol Mex Nueva Ser. 2024;10: 20241001. https://doi.org/10.53749/fem.2024.10.01
111. Leavengood JM Jr. Corrections and additions to the Hydnocerina (Coleoptera: Cleridae: Clerinae: Hydnocerini) of Mexico. Insecta Mundi. 2024;1054: 1–5. https://digitalcommons.unl.edu/insectamundi/1555
112. Hernández-May MA, Pérez-De la Cruz M, Toledo-Hernández VH, Rifkind J, Leavengood JM Jr, Guzmán-Vega GA, Niño-Maldonado S. Preliminary survey of the checkered beetles (Coleoptera: Cleridae) of Tamaulipas, Mexico. Coleopt Bull 2021;75(3): 661–669. https://doi.org/10.1649/0010-065X-75.3.661
113. Hernández-May MA, Leavengood JM Jr, Gerónimo-Torres JDC, Pérez-De La Cruz M, Niño-Maldonado S. New records of *Phyllobaenus* Dejean (Coleoptera: Cleridae: Clerinae: Hydnocerini) from Mexico. Southwest Entomol. 2022;47(4): 865–870. https://doi.org/10.3958/059.047.0409
114. Fikáček M, Maruyama M, Komatsu T, von Beeren C, Vondráček D, Short AEZ. Protosternini (Coleoptera: Hydrophilidae) corroborated as monophyletic and its larva described for the first time: a review of the myrmecophilous genus *Sphaerocetum*. Invertebr Syst 2015; 29(1): 23–36. http://dx.doi.org/10.1071/IS14026
115. Hölldobler B, Wilson EO. The ants. Cambridge, MA Harvard University Press; 1990.
116. Kistner DH. The social insects' bestiary. In: Hermann HR, editor. Social insects. New York: Academic Press; 1982. pp. 1–244.
117. von Beeren C, Brückner A, Hoenle PO, Ospina-Jara B, Kronauer DJC, Blüthgen N. Multiple phenotypic traits as triggers of host attacks towards ant symbionts: body size, morphological

gestalt, and chemical mimicry accuracy. Front Zool. 2021;18: 46. https://doi.org/10.1186/s12983-021-00427-8

118. Pérez-Lachaud G, Degallier N, Gomy Y, Elías-Gutiérrez M, Rocha FH, Lachaud J-P. Cohabitation with aggressive hosts: description of a new microhisterid species in nests of a ponerine ant with ecological notes. Sci Rep. 2023;13: 18484. https://doi.org/10.1038/s41598-023-45692-1

119. Pérez-Lachaud G, Jervis MA, Reemer M, Lachaud J-P. An unusual, but not unexpected, evolutionary step taken by syrphid flies: the first record of true primary parasitoidism of ants by Microdontinae. Biol J Linn Soc. 2014;111(2): 462–472. https://doi.org/10.1111/bij.12220

120. Godfray HCJ. Parasitoids: behavioral and evolutionary ecology. Princeton: Princeton University Press; 1994.

Figure captions

Fig 1. Location of the study sites in the coastal plain of Oaxaca, Mexico. A) Ranchería Yerba Santa; B) Piedras negras; C) El Zapotal (Mazunte); D) Puente Cuatode; E) Bajos de Coyula. Map credits: Holger Weissenberger.

Fig 2. *Phyllobaenus obscurus* (Coleoptera: Cleridae). A) A third instar larvae that have just exited its host cocoon. B) Remains of an *Ectatomma* sp. 3 pupa from which a mature beetle larva has emerged (the dissected cocoon can be observed). C) *P. obscurus* adult reared from the larva that parasitized the pupae in (B). Photo credits: (A-B) G. Pérez-Lachaud, (C) Humberto Bahena-Basave.

Fig 3. *Phyllobaenus obscurus* larva and remains of the host within the host cocoon. A) Third instar *P. obscurus* larva, dorsal view. B) Same, ventral view. C) Dorsal view of the head of a third instar larva, low vacuum SEM photography. D) An *Ectatomma* sp. 6 cocoon showing an exit hole after departing of a *P. obscurus* larva; the ant remains are visible. Photo credits: (A-B, D) Humberto Bahena-Basave; (C) G. Pérez-Lachaud and M. Elías-Gutiérrez.

Table Captions

Table 1. Summary of known rearing records among North American Cleridae and larval feeding mode on insects other than wood, stem and twig borers.

Table 2. Summary of the collection sites in the Oaxaca coast (Mexico) and number of colonies of the different *Ectatomma ruidum* species sampled during 2015-2017.

Table 3. Infestation rate of *Ectatomma ruidum* spp. cocoons by *Phyllobaenus obscurus*.

Supplementary material

S1 Video. A *Phyllobaenus obscurus* larva that has just emerged from its *Ectatomma ruidum* sp. 3 host cocoon.

S1Table. Detailed collection data for the three species of the *Ectatomma ruidum* complex studied: species concerned, location, date of collection, colony composition. μQ: number of microgynes.

Figure 1

Click here to access/download;Figure;Fig1.tiff

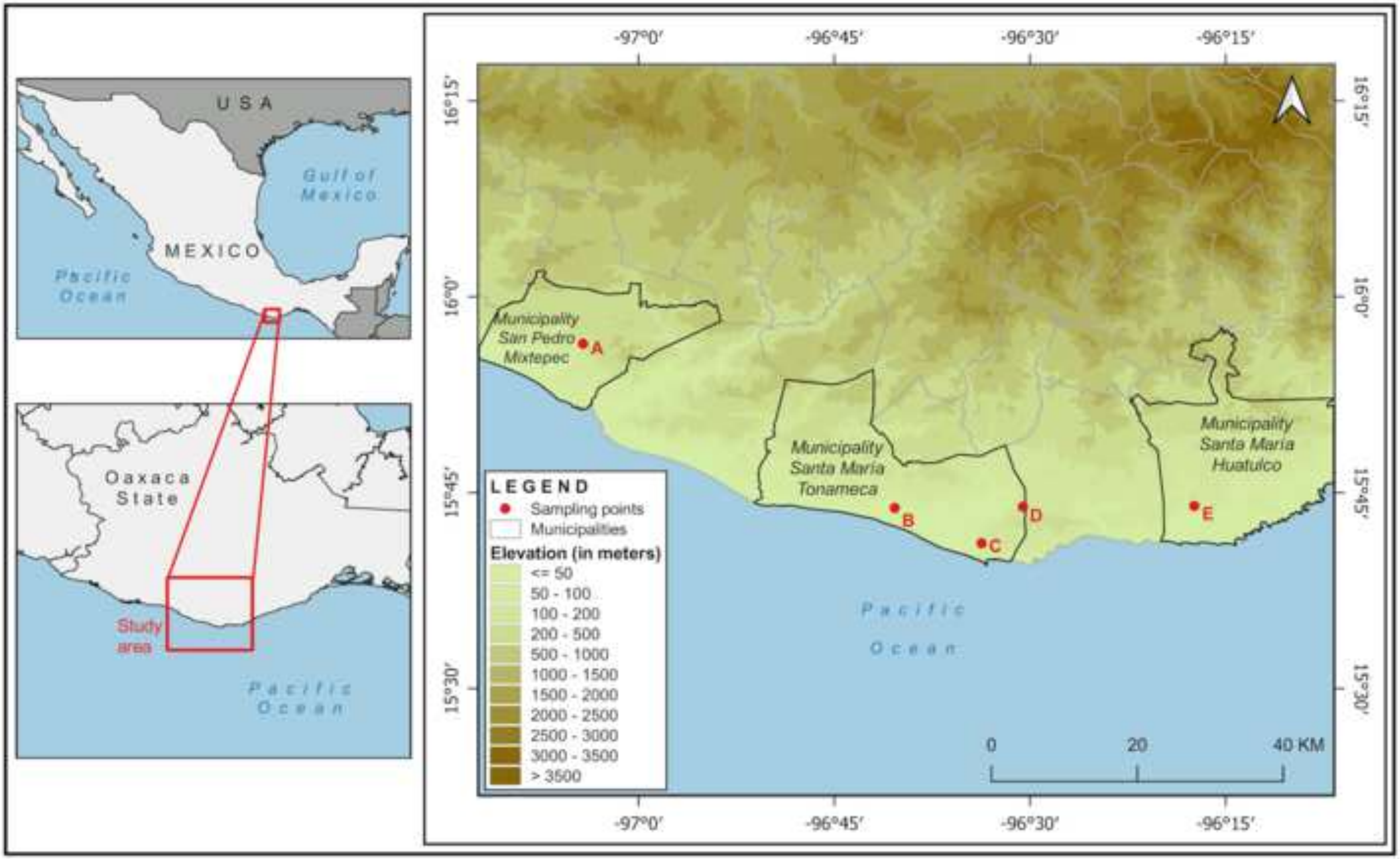

Figure 2

Click here to access/download;Figure;Fig2.tiff

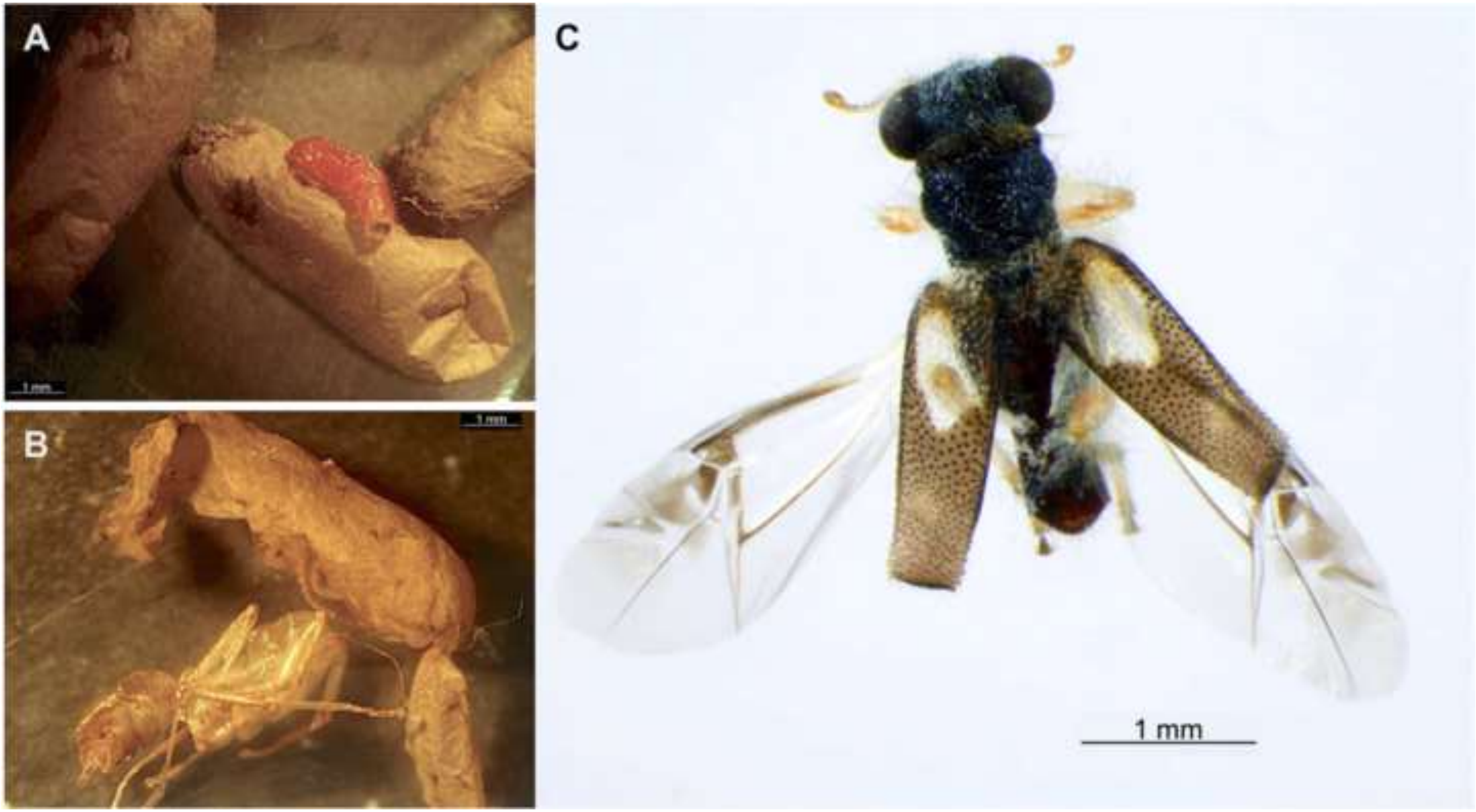

Figure 3

Click here to access/download;Figure;Fig3.tiff

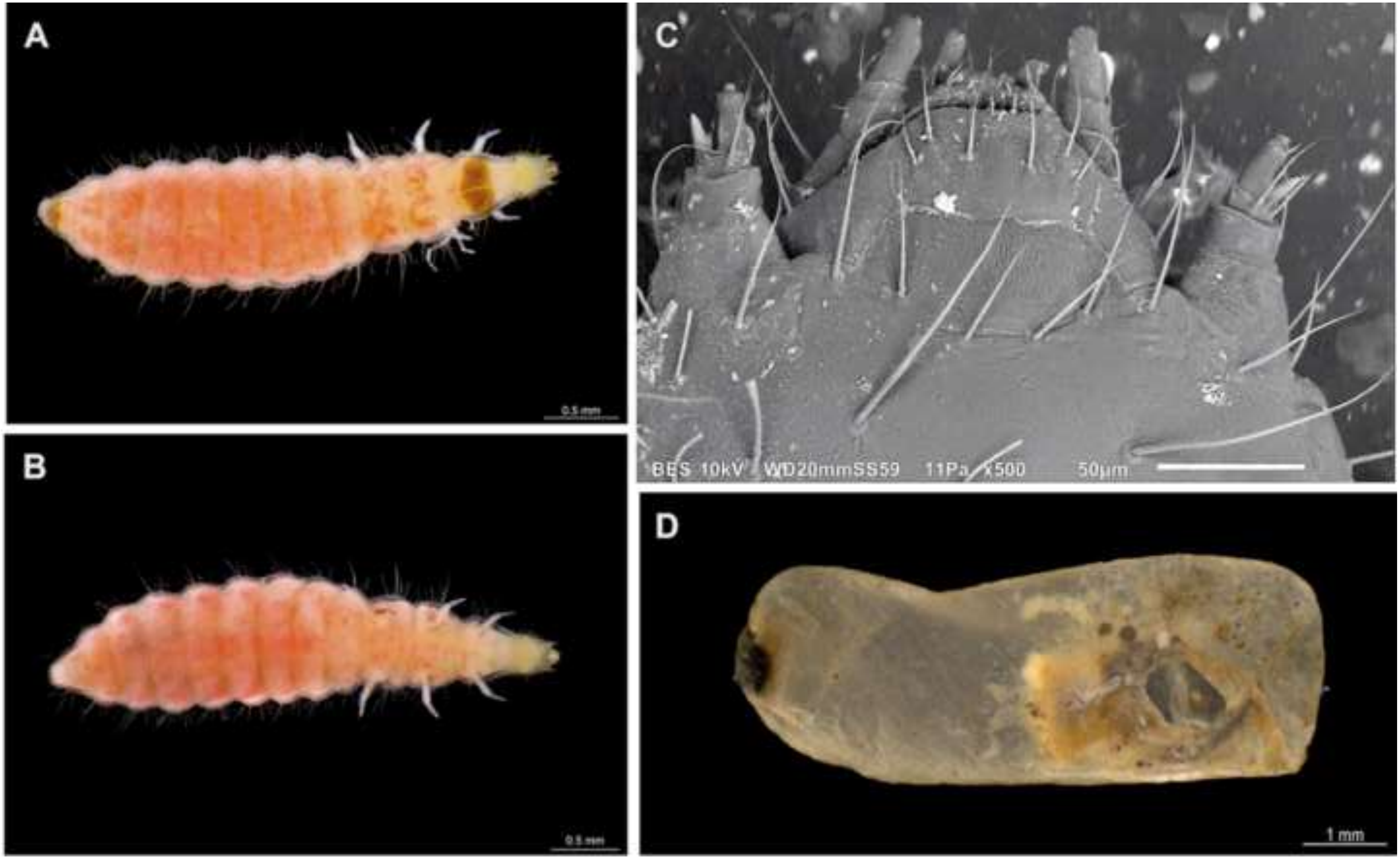

S1 Table

Click here to access/download
Supporting Information
S1Table.xlsx

S1 Video

Click here to access/download
Supporting Information
S1Video.mov

Click here to access/download
Supporting Information
Personal communication confirmation letter.pdf
