# Supplementary material for: Ant parasitoidism in checkered beetles: Phyllobaenus obscurus developing inside intact cocoons of two species of the Ectatomma ruidum species complex
Source: PLoS One. 2026 Mar 20;21(3):e0335300. doi: 10.1371/journal.pone.0335300 (PMC13004353; doi:10.1371/journal.pone.0335300)
Supplement: Attachment — Submitted filename: Manuscript Review PONE-D-25-54426.docx [file pone.0335300.s003.docx]

**Review by Exélis Moïse Pierre**

**Manuscript PONE-D-25-54426, entitled "Ant parasitoidism in checkered beetles: *Phyllobaenus obscurus* developing inside intact cocoons of two species of the *Ectatomma ruidum* species complex".**

**Overall assessment statement**

Thank you for inviting me to review this interesting and well-documented article.

The authors reported a rare case of parasitism from the predatory *Phyllobaenus obscurus* beetles on the *Ectatomma ruidum* species of ant cocoons. The discovery represents a novel adaptation for the beetle family.

The presentation of the complex and yet-to-be-further-classified ant host species adds more interest to the study findings. Based on molecular, chemical, and acoustic monikers, and on behavior, the existence of cryptic species is strongly suggested—an ant species whose description is difficult due to the high similarity of morphometric parameters. If the main objective is to demonstrate the parasitism trait, it gives an insight into further opportunities for taxonomic investigation of the species known for food thievery (cleptobiosis). This kind of research is valuable to the academic and student researcher community as a whole. For this reason, I strongly recommend the study for consideration for publication.

However, the complex nature of the topic requires robust knowledge to understand it at best, hence I provided some basic suggestions to improve and facilitate the first-time readers' comprehension.

**Abstract**

Well presented. It helped to understand the subject of research for a novice reader.

With the low occurrence of inferior to 0.6% parasitism of ant brood, could this be a case of accidental disorientation? Or just a facultative parasitism strategy for survival benefit? The authors clearly stated that the facultative or obligate pattern of the beetles is not defined in this study and needs to be carried out in the future. However, the absence of more comments on this poor occurrence value left me pondering on the elusive character of this newly discovered opportunistic parasite. See more comments in the methodology and results section review.

This point could be discussed briefly at the end of the discussion with robust citations to support it.

**Introduction**

This is a very technical and lengthy presentation of both the host and the parasite agent.

This was necessary due to the lack of documentation involving the concerned beetle's family.

Expertise is demonstrated in the literature review, and the study objectives and relevant aims are clearly explained. The authors offer to contribute to filling the knowledge gap on the natural histories of the checkered beetles of the genus *Trichodes*, better known as cleptoparasitoids and dominantly act as predators.

*Phyllobaenus obscurus*, the species under study here, is investigated for shifting from its habitual predatory behaviour to a rare case of undefined facultative or obligate parasitism on the immature stage of the brood ant species *Ectatomma ruidum*. I do not find any serious points missing in this manuscript. It looks almost perfect to me. The work is consistent.

However, I have some suggestions as follows -

Looking at the following:

- [Luong Lien T.](https://royalsocietypublishing.org/author/Luong%2C+Lien+T) and

- [Mathot Kimberley J.](https://royalsocietypublishing.org/author/Mathot%2C+Kimberley+J)

2019Facultative parasites as evolutionary stepping-stones towards parasitic lifestyles*Biol. Lett.***15**20190058[http://doi.org/10.1098/rsbl.2019.0058](https://doi.org/10.1098/rsbl.2019.0058)

I was searching for a point that could be interesting to add to the manuscript. I understand that, in fact, the study findings exposed a case without defining it as facultative or obligate parasitism. A complete mystery.

In light of the cited article above, I believe that it is judicious to give a brief & sound explanation of the ecological role and reason behind the occurrence of facultative & obligate parasitism.

So the general public can relate the facts better and have an added value, helping to see in a broader sense the context being investigated.

So the readers are invited from the very beginning to the core subject and avoid any potential confusion to better comprehend the impact and significance of the study.

Because the suggestion and hypothesis that facultative parasitism is a prelude to achieving the transition to obligate parasitoids is very strong and indeed never been reported in any insects (Luong Lien & Kimberley, 2019).

I understand that such adoption of parasitism for a predator is linked to the environmental challenges (natural food resources depletion), and a selected response to ensure the survival of a species. The core study aim is not directly related to this topic, but there is an indirect association that needs to be addressed. I am confident that the authors will know how best to fit it without falling into an overly extended discourse.

This article sounds good to look at:
Yeh, Y. H., Deto, H., & Miyashita, T. (2025). Disentangling biotic and abiotic factors influencing host–parasitoid interactions of butterflies, tachinid flies, and nematodes. *Ecological Research*, *40*(5), e70000.

In light of the following article:

Lachaud, J. P., & Pérez-Lachaud, G. (2012). Diversity of species and behavior of hymenopteran parasitoids of ants: a review. *Psyche: A Journal of Entomology*, *2012*(1), 134746.

A summary addition of the why facultative or obligate parasitism behaviour to the manuscript would facilitate a better comprehension of this difficult subject due to information scarcity for a novel behaviour of this species of checkered beetles acting as parasitoids of ants. The flow of ideas becomes smoother, better interconnected for beginners (my humble opinion). It renders the text all public readers friendly. Some people are not keen to do the extra documentation research and expect the “golden plate detailed manuscript” to avoid time-consuming reading activity & analysis.

**Methodology**

The ant species host is understudied for its potential of existing cryptic species, according to a majority of reports. This point gives more interest to this study and could trigger more willingness for further investigations.

Because the concept of ecological competition among co-dominant ant species within the same occupied territory, in light of their responses to their phorid parasitism, is paramount (Orr et al., 2003). This included *Ectatomma brunneum* acting as the main competitor of *Linepithema piliferum*.

Orr, M. R., Dahlsten1, D. L., & Benson, W. W. (2003). Ecological interactions among ants in the genus *Linepithema*, their phorid parasitoids, and ant competitors. *Ecological Entomology*, *28*(2), 203-210.

The methodology is straightforward to replicate.

Lines 175-176: The Pacific Coastal Plain of Oaxaca, Mexico. Could the authors precise if the zone has been safe from anthropogenic extensive activity that could alter the natural insect fauna population? Are there any agricultural land or industrial-level operations?

There is no statistical analysis proposed. In view of the findings, which are more descriptive of a novel behavior, it might not be compulsory to do so.

**Results**

The findings are convincing. The authors could not decide on the facultative or obligate nature of the checkered beetles' behaviour.

The very low occurrence of 0.6% needs to be discussed further, and tentative explanations provided in light of previous reports in the discussion.

**Discussion**

Line. 260: … “or their very movement patterns” – I suggest specifying which they are by giving a couple of examples.

Lines 340-345:

The report of the American checkered bark beetle destroyer *Thanasimus dubius* predation, acting as a biological control agent of the Southern pine beetle Dendroctonus frontalis, the most damaging native insect pest in the USA Southern forests (Cronin et al., 2000), is another indicator. It could be possible that an unknown ability, such as being a natural enemy of specific, limited pest taxa, has not yet been identified for this species *Phyllobaenus obscurus.*

Cronin, J. T., Reeve, J. D., Wilkens, R., & Turchin, P. (2000). The pattern and range of movement of a checkered beetle predator relative to its bark beetle prey. *Oikos*, *90*(1), 127-138.

Could it be that *Phyllobaenus obscurus* is a specialized predator that preys on a target population that is depleted or at low population density compared to the beetle? The application of extensive pesticides and agricultural toxic chemicals can collapse the population dynamics of insects.

Hence, for survival, a desperate move of parasitism at low occurrence is detected.

In the manuscript, the result of 0.6% parasitism occurrence is not discussed. There is no statistical analysis, but this value is important. I hope the authors could propose some ideas based on their expertise in this field of research. Otherwise, without a minimum of specific suggestions on this, it leaves the study report incomplete, as to leaves the readers to imagine by themselves. I believe this point to be a cornerstone of the study and needs to be addressed with a valuable analysis based on related existing reports, such as a few of them proposed in the review.

The study is worth publishing, but please let us see the best inspiration of comments to solve this mystery with some solid citations.

I hope that this review may add some elements enhancing the broader impact of the study for further research.
