# Supplementary material for: Ant parasitoidism in checkered beetles: Phyllobaenus obscurus developing inside intact cocoons of two species of the Ectatomma ruidum species complex
Source: PLoS One. 2026 Mar 20;21(3):e0335300. doi: 10.1371/journal.pone.0335300 (PMC13004353; doi:10.1371/journal.pone.0335300)
Supplement: Attachment — Submitted filename: Responses to reviewers.docx [file pone.0335300.s006.docx]

**[All references to lines in the revised version text refer to the revised version with tracked changes]**

**Journal Requirements**

When submitting your revision, we need you to address these additional requirements.

1. Please ensure that your manuscript meets PLOS ONE's style requirements, including those for file naming. The PLOS ONE style templates can be found at

<https://journals.plos.org/plosone/s/file?id=wjVg/PLOSOne_formatting_sample_main_body.pdf> and

[https://journals.plos.org/plosone/s/file?id=ba62/PLOSOne_formatting_sample_title_authors_affiliations.pdf](https://nam11.safelinks.protection.outlook.com/?url=https%3A%2F%2Ftrack.editorialmanager.com%2FCL0%2Fhttps%3A%252F%252Fjournals.plos.org%252Fplosone%252Fs%252Ffile%253Fid%3Dba62%252FPLOSOne_formatting_sample_title_authors_affiliations.pdf%2F1%2F010f019ab3710471-b839b3a1-2227-4856-9bff-b921b6f8d515-000000%2Fz5m09XxU55IL-f-os1j5GxiLVrpKdzx5u5wdM8pVF-k%3D238&data=05%7C02%7Cjlachaud%40ecosur.mx%7C017b0f55a59b4abe0a6008de2af76785%7C8d3be4b9be65459887c3af3137f93535%7C0%7C0%7C638995439599019767%7CUnknown%7CTWFpbGZsb3d8eyJFbXB0eU1hcGkiOnRydWUsIlYiOiIwLjAuMDAwMCIsIlAiOiJXaW4zMiIsIkFOIjoiTWFpbCIsIldUIjoyfQ%3D%3D%7C0%7C%7C%7C&sdata=%2BV8da2klZ5h8uydZMAftdK%2BmUYvnd4Qk22TiMWucnkc%3D&reserved=0)

R: This has been checked.

2. In your Methods section, please provide additional information regarding the permits you obtained for the work. Please ensure you have included the full name of the authority that approved the field site access and, if no permits were required, a brief statement explaining why.

R: Done (see l. 263-272 in the revised version).

3. Thank you for stating in your Funding Statement:

This study was partially funded by the project number M12A01, Ecos-Nord-CONACYT Program granted to CP and J-PL.

Please provide an amended statement that declares *all* the funding or sources of support (whether external or internal to your organization) received during this study, as detailed online in our guide for authors at <http://journals.plos.org/plosone/s/submit-now>.  Please also include the statement “There was no additional external funding received for this study.” in your updated Funding Statement.

Please include your amended Funding Statement within your cover letter. We will change the online submission form on your behalf.

R: Done (see the Cover Letter).

4. Thank you for stating the following financial disclosure:
This study was partially funded by the project number M12A01, Ecos-Nord-CONACYT Program granted to CP and J-PL.

Please state what role the funders took in the study. If the funders had no role, please state: "The funders had no role in study design, data collection and analysis, decision to publish, or preparation of the manuscript."
If this statement is not correct you must amend it as needed.
Please include this amended Role of Funder statement in your cover letter; we will change the online submission form on your behalf.

R: Done (see the Cover Letter).

5. We note that Figure 1 in your submission contain map images which may be copyrighted. All PLOS content is published under the Creative Commons Attribution License (CC BY 4.0), which means that the manuscript, images, and Supporting Information files will be freely available online, and any third party is permitted to access, download, copy, distribute, and use these materials in any way, even commercially, with proper attribution. For these reasons, we cannot publish previously copyrighted maps or satellite images created using proprietary data, such as Google software (Google Maps, Street View, and Earth). For more information, see our copyright guidelines: <http://journals.plos.org/plosone/s/licenses-and-copyright>.

We require you to either (a) present written permission from the copyright holder to publish these figures specifically under the CC BY 4.0 license, or (b) remove the figures from your submission:

a. You may seek permission from the original copyright holder of Figure 1 to publish the content specifically under the CC BY 4.0 license.

We recommend that you contact the original copyright holder with the Content Permission Form (http://journals.plos.org/plosone/s/file?id=7c09/content-permission-form.pdf) and the following text:

“I request permission for the open-access journal PLOS ONE to publish XXX under the Creative Commons Attribution License (CCAL) CC BY 4.0 (http://creativecommons.org/licenses/by/4.0/). Please be aware that this license allows unrestricted use and distribution, even commercially, by third parties. Please reply and provide explicit written permission to publish XXX under a CC BY license and complete the attached form.”

Please upload the completed Content Permission Form or other proof of granted permissions as an ""Other"" file with your submission.

In the figure caption of the copyrighted figure, please include the following text: “Reprinted from [ref] under a CC BY license, with permission from [name of publisher], original copyright [original copyright year].”

b. If you are unable to obtain permission from the original copyright holder to publish these figures under the CC BY 4.0 license or if the copyright holder’s requirements are incompatible with the CC BY 4.0 license, please either i) remove the figure or ii) supply a replacement figure that complies with the CC BY 4.0 license. Please check copyright information on all replacement figures and update the figure caption with source information. If applicable, please specify in the figure caption text when a figure is similar but not identical to the original image and is therefore for illustrative purposes only.

The following resources for replacing copyrighted map figures may be helpful:

USGS National Map Viewer (public domain): <http://viewer.nationalmap.gov/viewer/>

The Gateway to Astronaut Photography of Earth (public domain): <http://eol.jsc.nasa.gov/sseop/clickmap/>

Maps at the CIA (public domain): <https://www.cia.gov/library/publications/the-world-factbook/index.html> and <https://www.cia.gov/library/publications/cia-maps-publications/index.html>

NASA Earth Observatory (public domain): <http://earthobservatory.nasa.gov/>

Landsat: <http://landsat.visibleearth.nasa.gov/>

USGS EROS (Earth Resources Observatory and Science (EROS) Center) (public domain): [http://eros.usgs.gov/#](http://eros.usgs.gov/)

Natural Earth (public domain): <http://www.naturalearthdata.com/>

R: All permission letters have been joined (see uploaded files noted as "Other files").

6. If the reviewer comments include a recommendation to cite specific previously published works, please review and evaluate these publications to determine whether they are relevant and should be cited. There is no requirement to cite these works unless the editor has indicated otherwise.

R: All articles suggested by one of the reviewers (Reviewer #1) were checked before responding to his comments.

7. Please review your reference list to ensure that it is complete and correct. If you have cited papers that have been retracted, please include the rationale for doing so in the manuscript text, or remove these references and replace them with relevant current references. Any changes to the reference list should be mentioned in the rebuttal letter that accompanies your revised manuscript. If you need to cite a retracted article, indicate the article’s retracted status in the References list and also include a citation and full reference for the retraction notice.

R: This has been checked.

**Additional Editor Comments**

The claim that this is the first beetle species acting as a parasitoid of ants may be too definitive. Several beetle groups show predatory or brood-infiltrating behaviors inside ant nests, and some approach the boundary between predation and parasitism. Without a clear explanation of how those cases are excluded, the statement risks sounding broader than the available evidence supports.

The line separating predation, facultative parasitoid behavior, and true parasitoid development is not sharply drawn in the manuscript. Cleridae are known for flexible feeding strategies, and the observations described could fit multiple life history modes. The interpretation would benefit from clearer criteria distinguishing these possible strategies to support the argument for parasitoidism.

R: While parasitoidism was clearly defined in the previous version (lines 44-46), its distinction from predation was not clearly stated. We have corrected this omission (l. 52-53 of the revised version). We also agree that the discussion in the previous version of our manuscript did not sufficiently emphasize the arguments in favor of interpreting our results as evidence of parasitoidism by *P. obscurus*, and we thank you for pointing this out. We have thoroughly revised the discussion, rewording some parts and, we hope, presenting strong arguments in favor of this lifestyle in a more convincing manner. In addition, we now present further evidence that reduces uncertainty regarding the species identity. The (COI) sequence of one larva was obtained and used to connect the adult and the early stages of this species.

Although the number of cases reported here is small, the evidence currently available clearly indicates that we are dealing with a case of parasitoidism. Indeed, each of the three *P. obscurus* larvae found on an ant pupa was enclosed in an intact cocoon (neither pierced nor opened) of *E. ruidum* sp. 4, suggesting that an early larval stage was already anchored to the ant larva when the latter weaved its cocoon. Furthermore, at least one adult was reared from a larva that had emerged from a cocoon of *E. ruidum* sp. 3 by piercing an exit hole. This larva pupated and attained adulthood without further feeding upon ant larvae or pupae. The fact that *P. obscurus* eggs or any triungulin larvae have never been found inside *E. ruidum* nests does not detract from the fact that each beetle larva developed at the expense of a single ant pupa, which as a result was emptied of its substance and died, which is the very definition of the term parasitoid. The facts observed and reported in our manuscript do not correspond in any way to the cases of predation reported for many other beetles, such as rove beetles and other checkered beetles, where several individual hosts are commonly preyed upon. In our case, inside the cocoons, only one host was available and large enough to allow the development of at least one parasitoid the size of *P. obscurus*. These arguments have been presented and discussed in a paragraph added to the discussion.

The absence of triungulin larvae, eggs, or other early developmental stages associated with ant nests leaves an important gap. Many Cleridae with parasitic or phoretic life cycles rely on triungulin stages for host access, so not finding these forms makes the developmental pathway uncertain. Further clarification of early stages would help support the proposed life history model.

R: We agree with the editor that the most likely mode of infestation of nests is via triungulin larvae, as is the case with various other parasitic Cleridae. We had already included a sentence in the discussion clarifying this point (see l. 334-336 in the previous version).

This study is part of a larger project focusing on genetic/behavioral/acoustic/chemical/morphological variability of species in the *Ectatomma ruidum* complex. At the time of collection, we were unaware of the presence of the clerid, and the workers and larvae were only inspected externally. The planidia of some eucharitids may be found in the infrabucal pocket of foraging workers and then transmitted to the ant larvae during feeding. We did not check the workers as thoroughly and this will need to be done in the future. However, as noted above, *P obscurus* larvae were found feeding on ant pupae inside their cocoons. Since the cocoon is woven by the late-instar ant larva, we assume that the triungulin had already attached itself to its host at that moment and was enclosed in the cocoon where it was invisible to the workers of these aggressive ants.

The identification of larvae as belonging to the same species as the reared adult remains tentative without molecular confirmation. Only one adult was successfully obtained, and the remaining larvae are attributed to the same species based solely on morphology and context. Genetic verification would help solidify this assignment and reduce uncertainty regarding species identity.

R: Thank you for highlighting this point. You are right, when we submitted our previous version, we assigned the adult and the larvae to the same species based solely on morphology and context, comforted by our experience and knowledge of ant parasitoids. However, we were in the process of sequencing some specimens. Unfortunately, not all specimens amplified. We have now obtained a sequence for one of the beetle larvae, which is close to, but distinct from, the sequences of several *Phyllobaenus* species available in public databases. We have added this information both in the “Material and Methods” and “Results” sections (see l. 248-256 and 327-337 of the revised version). Unfortunately, we were unable to obtain a sequence for the adult (poor condition of the voucher and fungal contamination), and there are no sequences for *P. obscurus* in public databases. We hope that, despite the sequencing problems, the new results will reduce uncertainty regarding the identity of the species.

Some sections present evolutionary explanations in a way that can appear more conclusive than the evidence permits. Arguments about predispositions toward myrmecophily or transitions toward parasitoidism would benefit from phrasing that distinguishes speculation from established fact. Clarifying these boundaries would provide a more balanced interpretation.

The discussion of how larvae or adults avoid ant aggression is presented without accompanying behavioral observations. Without direct evidence of interactions between the beetle life stages and the ants, these explanations rely on inference rather than documentation. A more cautious description would better reflect what is known and what remains to be studied.

R: Thank you for bringing this issue to our attention. We have deleted some sentences and reworded some paragraphs accordingly. We have also expanded on the points that should be clarified in future studies.

**Reviewers' comments**

**Reviewer #1:**

Thank you for inviting me to review this interesting and well-documented article. The authors reported a rare case of parasitism from the predatory Phyllobaenus obscurus beetles on the Ectatomma ruidum species of ant cocoons. The discovery represents a novel adaptation for the beetle family.
The presentation of the complex and yet-to-be-further-classified ant host species adds more interest to the study findings. Based on molecular, chemical, and acoustic monikers, and on behavior, the existence of cryptic species is strongly suggested—an ant species whose description is difficult due to the high similarity of morphometric parameters. If the main objective is to demonstrate the parasitism trait, it gives an insight into further opportunities for taxonomic investigation of the species known for food thievery (cleptobiosis). This kind of research is valuable to the academic and student researcher community as a whole. For this reason, I strongly recommend the study for consideration for publication.

However, the complex nature of the topic requires robust knowledge to understand it at best, hence I provided some basic suggestions to improve and facilitate the first-time readers' comprehension.
See the attached review document please.

**Additional Comments:**

**Abstract**

Well presented. It helped to understand the subject of research for a novice reader.

With the low occurrence of inferior to 0.6% parasitism of ant brood, could this be a case of accidental disorientation? Or just a facultative parasitism strategy for survival benefit? The authors clearly stated that the facultative or obligate pattern of the beetles is not defined in this study and needs to be carried out in the future. However, the absence of more comments on this poor occurrence value left me pondering on the elusive character of this newly discovered opportunistic parasite. See more comments in the methodology and results section review.

This point could be discussed briefly at the end of the discussion with robust citations to support it.

R: It seems unlikely that we observed larvae inside intact cocoons by chance or that this was a case of accidental disorientation, especially since ants of the genus *Ectatomma* are efficient opportunistic predators and attack most insects they encounter, and even more so if they are near or inside their nests (see Dejean and Lachaud 1992 [Insect Soc. 1992;39(2):129–143]; Schatz et al. 1996 [Insect Soc. 1996;43(2):111–118]). It is therefore completely implausible that a beetle such as *P. obscurus*, or any other foreign individual, could land by chance (or by mistake) inside the colony, in the middle of the brood. Moreover, this happened five times in total in our study, including the case where we found a cocoon of *E. ruidum* sp. 4 with an exit hole and the remains of the host ant’s pupa inside. Although in some case studies myrmecophiles may be very abundant, in fact extremely low levels of parasitism prevalence are quite common among many ant parasitoids (Lachaud & Passera 1982; Hansson et al. 2011; von Beeren et al. 2011; Pérez-Lachaud & Lachaud 2014, 2017, 2021; Rocha et al. 2020; Pérez-Lachaud et al. 2021 [see references [9,128–134] in the revised version]). For example, only eight cocoons were found to be parasitized by eucharitid wasps in this study, while the presence of certain beetles in the raids of army ants of the genus *Leptogenys* could be very rare, such as *Witteia dentilabrum* and *Togpelenys gigantea* associated with *Leptogenys distinguenda*, or *Leptogenonia roslii* associated with *Leptogenys borneensis* (von Beeren et al. 2011 [see reference [130] in the revised version]). Although parasitoidism causes the death of the host at the individual level, in the case of social insects as host, the outcome at the colony level is more akin to that of a chronic disease. This is certainly one of the reasons why so few parasitoids actually have the potential to act as biological control agents against pest ants. This clarification has been added and expanded upon in an additional paragraph in the discussion (see l. 439-464 of the revised version).

**Introduction**

This is a very technical and lengthy presentation of both the host and the parasite agent.

This was necessary due to the lack of documentation involving the concerned beetle's family.

Expertise is demonstrated in the literature review, and the study objectives and relevant aims are clearly explained. The authors offer to contribute to filling the knowledge gap on the natural histories of the checkered beetles of the genus *Trichodes*, better known as cleptoparasitoids and dominantly act as predators.

*Phyllobaenus obscurus*, the species under study here, is investigated for shifting from its habitual predatory behaviour to a rare case of undefined facultative or obligate parasitism on the immature stage of the brood ant species *Ectatomma ruidum*. I do not find any serious points missing in this manuscript. It looks almost perfect to me. The work is consistent.

However, I have some suggestions as follows -

Looking at the following: [Luong Lien T.](https://royalsocietypublishing.org/author/Luong%2C+Lien+T) and [Mathot Kimberley J.](https://royalsocietypublishing.org/author/Mathot%2C+Kimberley+J) 2019. Facultative parasites as evolutionary stepping-stones towards parasitic lifestyles. *Biol. Lett.***15:** 20190058 <http://doi.org/10.1098/rsbl.2019.0058>

I was searching for a point that could be interesting to add to the manuscript. I understand that, in fact, the study findings exposed a case without defining it as facultative or obligate parasitism. A complete mystery.

In light of the cited article above, I believe that it is judicious to give a brief & sound explanation of the ecological role and reason behind the occurrence of facultative & obligate parasitism.

So the general public can relate the facts better and have an added value, helping to see in a broader sense the context being investigated.

So the readers are invited from the very beginning to the core subject and avoid any potential confusion to better comprehend the impact and significance of the study.

Because the suggestion and hypothesis that facultative parasitism is a prelude to achieving the transition to obligate parasitoids is very strong and indeed never been reported in any insects (Luong Lien & Kimberley, 2019).

I understand that such adoption of parasitism for a predator is linked to the environmental challenges (natural food resources depletion), and a selected response to ensure the survival of a species. The core study aim is not directly related to this topic, but there is an indirect association that needs to be addressed. I am confident that the authors will know how best to fit it without falling into an overly extended discourse.

This article sounds good to look at: Yeh, Y. H., Deto, H., & Miyashita, T. (2025). Disentangling biotic and abiotic factors influencing host–parasitoid interactions of butterflies, tachinid flies, and nematodes. *Ecological Research*, *40*(5), e70000.

In light of the following article: Lachaud, J. P., & Pérez-Lachaud, G. (2012). Diversity of species and behavior of hymenopteran parasitoids of ants: a review. *Psyche: A Journal of Entomology*, *2012*(1), 134746.

A summary addition of the why facultative or obligate parasitism behaviour to the manuscript would facilitate a better comprehension of this difficult subject due to information scarcity for a novel behaviour of this species of checkered beetles acting as parasitoids of ants. The flow of ideas becomes smoother, better interconnected for beginners (my humble opinion). It renders the text all public readers friendly. Some people are not keen to do the extra documentation research and expect the “golden plate detailed manuscript” to avoid time-consuming reading activity & analysis.

R: This observation is obviously relevant, but taking it into account would take us considerably further away from our main objective (demonstrating the parasitoid trait of *Phyllobaenus obscurus* against *Ectatomma* ants), especially since we have no concrete evidence to support either hypothesis (facultative or obligatory parasitism) without resorting to pure speculation, even if facultative parasitism seems the most likely here. It would be essential to have a much larger sample of colonies, but this would be difficult to achieve without substantial financial resources and without the cooperation of the owners of the land where the ant colonies were excavated, an aspect that has already caused us quite a few problems in conducting this study (see l. 226-230 in the revised version and our response to comment "C4-L. 213" by Reviewer #4).

**Methodology**

The ant species host is understudied for its potential of existing cryptic species, according to a majority of reports. This point gives more interest to this study and could trigger more willingness for further investigations.

Because the concept of ecological competition among co-dominant ant species within the same occupied territory, in light of their responses to their phorid parasitism, is paramount (Orr et al., 2003). This included *Ectatomma brunneum* acting as the main competitor of *Linepithema piliferum*.

Orr, M. R., Dahlsten1, D. L., & Benson, W. W. (2003). Ecological interactions among ants in the genus *Linepithema*, their phorid parasitoids, and ant competitors. *Ecological Entomology*, *28*(2), 203-210.

The methodology is straightforward to replicate.

R: Such a study on the role of parasitism in competition between sympatric co-dominant species would of course be interesting, but it strays too far from the main objective of our study. Furthermore, given the extremely low prevalence of parasitism observed in our parasites, we are far from the conditions observed in the study conducted by Orr et al. (2003), where the phorid fly *Pseudacteon lontrae* was very abundant. And even under such favorable conditions, Orr and his colleagues were unable to draw definitive conclusions about the influence of parasitism prevalence on competition between *Ectatomma brunneum* and *L. piliferum*.

Lines 175-176: The Pacific Coastal Plain of Oaxaca, Mexico. Could the authors precise if the zone has been safe from anthropogenic extensive activity that could alter the natural insect fauna population? Are there any agricultural land or industrial-level operations?

R: The state of Oaxaca is located in southern Mexico, where the Eastern Sierra Madre and the Southern Sierra Madre mountain ranges merge. It covers an area of 36,820 square miles with 370 miles of Pacific coastline. There are several tourism conglomerates in this zone, but sampling was performed in relatively undisturbed area (see l. 205-208 in the revised version). It is one of the least developed states of Mexico in terms of industry, and only about 9% of Oaxaca’s land area is arable, with 6.3% of Oaxaca’s gross domestic product devoted to agriculture. We did not expand on this in our manuscript, but we clearly stated how the sites were selected.

There is no statistical analysis proposed. In view of the findings, which are more descriptive of a novel behavior, it might not be compulsory to do so.

**Results**

The findings are convincing. The authors could not decide on the facultative or obligate nature of the checkered beetles' behaviour.

The very low occurrence of 0.6% needs to be discussed further, and tentative explanations provided in light of previous reports in the discussion.

R: Please, see our response to the first comment.

**Discussion**

Line. 260: … “or their very movement patterns” – I suggest specifying which they are by giving a couple of examples.

R: Done (see l. 377-378 in the revised version).

Lines 340-345:

The report of the American checkered bark beetle destroyer *Thanasimus dubius* predation, acting as a biological control agent of the Southern pine beetle Dendroctonus frontalis, the most damaging native insect pest in the USA Southern forests (Cronin et al., 2000), is another indicator. It could be possible that an unknown ability, such as being a natural enemy of specific, limited pest taxa, has not yet been identified for this species *Phyllobaenus obscurus.*

Cronin, J. T., Reeve, J. D., Wilkens, R., & Turchin, P. (2000). The pattern and range of movement of a checkered beetle predator relative to its bark beetle prey. *Oikos*, *90*(1), 127-138.

Could it be that *Phyllobaenus obscurus* is a specialized predator that preys on a target population that is depleted or at low population density compared to the beetle? The application of extensive pesticides and agricultural toxic chemicals can collapse the population dynamics of insects.

Hence, for survival, a desperate move of parasitism at low occurrence is detected.

In the manuscript, the result of 0.6% parasitism occurrence is not discussed. There is no statistical analysis, but this value is important. I hope the authors could propose some ideas based on their expertise in this field of research. Otherwise, without a minimum of specific suggestions on this, it leaves the study report incomplete, as to leaves the readers to imagine by themselves. I believe this point to be a cornerstone of the study and needs to be addressed with a valuable analysis based on related existing reports, such as a few of them proposed in the review.

The study is worth publishing, but please let us see the best inspiration of comments to solve this mystery with some solid citations.

I hope that this review may add some elements enhancing the broader impact of the study for further research.

R: The considerations raised here by reviewer #1 are interesting but highly speculative, not to say overly speculative given the limited certainty obtained to date regarding the exact nature of the parasitoidism carried out by *Phyllobaenus obscurus* at the expense of the pupae of *E. ruidum* sp. 3 and sp. 4. Indeed, we cannot yet determine whether this parasitoidism is facultative or obligatory, although given the very low prevalence of parasitism found within populations of *E. ruidum* sp. 3 and sp. 4, and the endemism of these populations compared to the much wider distribution of *P. obscurus*, the strict specificity of the association with ants of the *E. ruidum* complex or the possibility of obligatory parasitism seem very unlikely. Furthermore, and for the same reasons, it is unlikely that *P. obscurus* is a predator specializing in prey whose distribution is extremely limited compared to that of the checkered beetle. The evidence currently available, namely the presence of various larval stages of *P. obscurus* inside intact (unpierced or unopened) cocoons of *E. ruidum* sp. 3 and sp. 4, and the rearing of at least one adult from one of these larval stages, clearly indicates that we are dealing with a case of parasitoidism. However, given the small number of cases reported here, any extrapolation to the impact of this parasitoid on the entire population of *E. ruidum* sp. 3 and sp. 4 would be risky, and any hypotheses formulated on this basis would therefore necessarily be speculative. We therefore considered it would be preferable to refrain from doing so.

**Reviewer #2:**

An interesting paper that should be published. In the absence of actually seeing beetle larvae attack and develop on the ants, the presented information is as good evidence of parasitoidism in this system as could be expected.

R: We would just like to point out here that, while it is true that the initial attack by *P. baenus* on its host could not be observed, its development at the expense of ant pupae was indeed observed on four nymphs: one of *E. ruidum* sp. 3 (collected in 2015), where the beetle larva was able to reach a stage of development that allowed it to emerge from the host cocoon and later transform into an adult, and three of *E. ruidum* sp. 4 (collected in 2016), where one first-instar larva and two second-instar larvae of *Phyllobaenus* were observed, each one on a nymph enclosed in an intact cocoon.

This paper is well written, and I cannot find anything significant I would want to see changed.

My only comment is italicize Trichodes on L 487.

R: Done (see l. 644 in the revised version).

**Reviewer #3: [Suprakash Pal; see PONE-D-25-54426_reviewed.pdf]**

The article has been written very well first time reporting the parasitization of Ectatomma ants by the beetle Phyllobaenus obscurus. This novel information will enrich the knowledge of Myrmecophily evolution in Checkered beetle belonging to the family Cleridae. I have found one typographical mistake which has been marked in the reviewed original manuscript attached with this report.

R: See response to Comment 1, below.

I suggest you to improve the quality of the photographs in Fig 2. The article deserves publication after minor correction. I congratulate all the authors for writing this article.

R: See response to Comment 2, below.

**Additional Comments:**

C1-L. 119: Change ‘wich’ by ‘which’.

R: Done (see l. 132 in the revised version).

C2-L. 689: ‘Fig 2’ Picture quality could have been better, if possible provide good quality photo with more clarity.

R: The photos presented in Fig. 2 are the only ones we have available. They were taken at the time of the unexpected observation of the first case indicating possible parasitoidism, and with the limited resources available at that time. However, we would like to point out that the quality of the photos presented in .TIFF format is much better than that of the photos appearing in the .PDF version that the reviewers had at their disposal when evaluating our manuscript. Unfortunately, we cannot do better in the case of this figure (now Figure 1 in the revised version).

**Reviewer #4: [see Ant parasitoidism in checkered beetles.pdf]**

Comments to the Author,
The manuscript "Ant parasitoidism in checkered beetles: Phyllobaenus obscurus developing inside intact cocoons of two species of the Ectatomma ruidum species complex" deals with the new record of Phyllobaenus obscurus as a parasitoid of ants of the Ectatomma ruidum species complex. The manuscript is generally very well written and easy to read. The comments I have to improve the manuscript and its presentation are, in my opinion, easy to address. The most significant would relate to the details of the methodology and the results. For example, it is not mentioned how many colonies were collected for recording until the emergence of the adult parasitoids, nor how many colonies were separated for dissection (details in the PDF).

R: See responses to Comments 2 and 3, below.

Regarding methodology and results, several aspects lack detail (see details in the PDF), for example, why are there many more colony records in 2016 compared to 2015? Was there a greater effort in the collections? Is that due to a larger area covered or a greater number of people recording colonies?

R: See response to Comment 4, below.

Finally, I think the discussion is quite long and doesn't always address the actual field results.

R: See response to Comment 5, below.

Furthermore, issues related to the low rate of parasitism weren't discussed or even compared with other studies (details of which are also included in the PDF).

R: See response to Comment 6, below.

**Additional Comments:**

C1-L. 46: Change ‘whereas adults’ by ‘whereas adults parasitoids organism…’

R: Done (see l. 55 in the revised version).

C2-L. 193: It is important to report the number of cocoons of each colony that were observed until the emergence of the parasitoid

R: All the information was already available in the “Results” section and in Tables 2, 3, and S1, but in a way that was apparently too dense or compact to be fully understandable. We have slightly revised the presentation of our Material and Methods and Results in the body of the manuscript, providing a little more detail on the various important elements obtained (attacks by nematodes and eucharitid wasps in particular). All the information is summarized in Tables 2 and 3, which have not been modified, and is detailed colony by colony, as requested, in Table S1, which was also already present in the initial version as supplementary material. However, some annotations that inadvertently appeared in Spanish in the previous version of this table S1 have been translated into English,

C3-L. 197: Does this mean that some cocoons were only observed until the emergence of the adult parasitoid, while others were dissected to examine the contents? If so, further clarification is needed, including the number of cocoons in each group.

R: No. The cocoons were not separated into two batches and all of them were examined under a stereomicroscope but in two successive stages. We have added further details in the Material and Methods section (see l. 234-243 of the revised version). In 2015 and 2017, all collected cocoons were first checked one by one to ensure they were all intact, then stored in Petri dishes. After waiting a few days to see if any parasitoid emerged from the cocoons (which was only the case for the *P. obscurus* larva observed in 2015), all remaining cocoons were dissected. In 2016, when sampling efforts were intensified, the number of complete colonies collected and the limited time available in the field to examine the cocoons, forced us to preserve all cocoons in ethanol. Back at our institution, all cocoons were individually inspected and dissected and their contents examined.

C4-L. 213: Some details are missing regarding the colony collections. For example, why are there many more colony records in 2016 compared to 2015? Was there a greater effort in the collections? That is, a larger area covered or a greater number of people recording colonies?

What explains the higher number of E. ruidum sp. 4 records in the collections? Was it the most abundant in the region? Would that partly explain the fact that it presented the highest number of parasitoids?

R: First, as stated in the previous version (lines 171-174), we must emphasize that the colonies excavations reported in this manuscript were carried out as part of a larger project aimed at characterizing/revealing the cryptic species of the *E. ruidum* complex and assessing the diversity of some of their already cataloged parasitoids. Collecting an equal number of nests from the different cryptic species of this ant complex was not a prerequisite for this program, and therefore no effort was made in this regard. Additional information has been added to the Materials and Methods section concerning the distribution/abundance of the different species of *E. ruidum* and the workforce available to conduct nest excavation (some nest chambers reached a depth of approximately 1.7 m). In 2015, only three people were available to carry out excavations, and 12 complete colonies were collected from three sites. In 2016, six people were available and we were able to excavate more nests at more sites (5), with a total of 71 complete colonies collected. In 2017, only two people were available and, in addition, access to certain collecting sites was impossible or denied due to socio-political tensions in the study area, reducing the number of collecting sites to just one, where 15 complete colonies were excavated. Furthermore, the most abundant and accessible species in the study area is *E. ruidum* sp. 4. The species *E. ruidum* sp. 2 (although the most common in Mexico) was only found in a low coastal forest area, and the distribution of *E. ruidum* sp. 3 is extremely limited, as it is known to occur at only one site that is no longer accessible. For more details on collection sites, see Peña-Carillo et al. 2022a, b; Meza-Lázaro et al. 2022 [see references [82–84] in the revised version]).

E. ruidum sp. 2 was not recorded as beetle larvae. Any idea why?

R: During this study, the species *E. ruidum* sp. 2 was the least sampled, with only 18 complete colonies collected with cocoons (a value close to that for *E. ruidum* sp. 3). However, this species has the widest distribution in Mexico, and we have had the opportunity to carry out numerous other excavations in Chiapas and Quintana Roo (more than 1,400 nests collected in 44 years), and no cases of attack by *P. obscuru*s have ever been observed, which is still quite significant. That said, we have no evidence to support any hypothesis to explain this result nor to explain the absence of mermithergates in *E. ruidum* sp. 4.

Finally, although this may remain somewhat speculative, what could explain the low rate of parasitism in the nests?

R: Please, see our response to reviewer #1 first comment: Although in some case studies myrmecophiles may be very abundant, in fact extremely low levels of parasitism prevalence are quite common among many ant. This clarification has been added and expanded upon in an additional paragraph in the discussion (see l. 439-464 of the revised version).

C5-L. 257: The first paragraph of the discussion seems more like an introduction; I suggest either shortening this paragraph or placing it in the introduction so that the discussion begins with what really matters.

R: We have modified the first paragraph of the discussion to highlight the results of this study but we have not followed the reviewer’s suggestion because, for reasons of logical consistency in the succession of the arguments presented, we feel it is inappropriate to introduce the species *P. obscurus* in the introduction when it has not yet been mentioned.

C6-L. 277: This entire paragraph can be shortened; it presents important information but does not discuss the results obtained regarding ant parasitism or their low infection rate.

R: We disagree with this comment because we believe that, in fact, what we present in this paragraph is quite pertinent. It is true that we do not discuss the low rate of parasitism, but we do delineate how our *P. obscurus* is an outlier among its congeners, and how such behaviors also align with the opportunity to encounter and evolve to capitalize upon ground-nesting ants. We do not see how to shorten this paragraph without having to tediously rephrase it to achieve a minimal reduction (using compound sentences).

**Reviewer #5:**

The paper deals with a potential case of ant parasitoidism by a clerid beetle, this is quite interesting since if confirmed would be the first case among coleoptera. The text is clear, and a good literature overview has been reported, although I suggest the Authors to better develop the case of other myrmecophilous parasite beetles (e.g. staphylinids) whose larvae feed on ant brood as a comparative case study.

R: A few examples of other myrmecophilous beetles have already been presented in the introduction. However, we did not want to delve deeper into the subject of social parasitism in beetles because we did not want to get sidetracked by the topic of predation and stray too far from our main objective, as it is parasitoidism that interests us most here.

The study is primarily descriptive and reports a general and detailed check of many ant colonies of the putative host species. The sample is quite large but only in a few cases beetle larvae emerging form ant cocoons (suggesting a case of parasitoidism) where recorded. Given the few evidence and the lack of information on the habits of adults, I suggest the Authors to not emphasize excessively the certain discovery of a new species of ant parasitoid. Hence, I suggest to better modulate the discussion, as well as the title of the paper accordingly, limiting to what exactly the observational data allow to state, such as first data on a possible case of parasitoidism by coleoptera on ant brood.

R: We disagree with this comment because, even though the number of cases is small, the evidence currently available clearly indicates that this is a case of parasitoidism (see our response to the academic editor's first comment). We also did not follow the reviewer's suggestion to change the title of the manuscript, but instead modified the discussion, providing more strong arguments to support our interpretation of the presence of *P. obscurus* in the nests of *E. ruidum* sp. 3 and sp. 4 as a genuine case of parasitoidism (see l. 362–373 in the revised version).

It should be noted that ant parasitoids are generally very rare symbionts that develop in well-defended fortresses which, in many cases, are located or built at places of limited access (high in the canopy or below ground level with chambers sometimes several meters deep). It is only by sampling entire colonies, which can number several thousand individuals, that researchers have been able to uncover such symbionts, and that new first observations at the family level have been reported over the two last decade. See for example the report of the first case of a cecidomyiid as parasitoid of weaver adult ants (*Oecophylla longinoda*) by Mekonnen et al. (2020) or our own works on the first case of encyrtid wasps as parasitoids of *Neoponera goldii* (Pérez-Lachaud et al. 2012) and on *Hypselosyrphus trigonus*, the only syrphid known to parasitize ant brood (Pérez-Lachaud et al. 2014, 2017) [see references [119–121,131] in the revised version. We are convinced that these discoveries are only the tip of the iceberg when it comes to the diversity of ant parasitoids.

A couple of other minor points on the text:
Lines 69-70: Diplopoda is not an Order.

R: Of course, the reviewer is right, since Diplopoda is a class. We have corrected this by simply removing the word “orders.”

Lines 138-140: revise the writing, the sentence is not clear.

R: Done (see l. 151–154 in the revised version).
