# Supplementary material for: Ant parasitoidism in checkered beetles: Phyllobaenus obscurus developing inside intact cocoons of two species of the Ectatomma ruidum species complex
Source: PLoS One. 2026 Mar 20;21(3):e0335300. doi: 10.1371/journal.pone.0335300 (PMC13004353; doi:10.1371/journal.pone.0335300)
Supplement: Attachment — Submitted filename: Response to editors comments.docx [file pone.0335300.s007.docx]

Responses to the editor

1- The adult specimen identified morphologically as Phyllobaenus obscurus was not sequenced, because extraction failed due to poor condition, and no adult COI is available for direct comparison. This leaves a critical gap in the identity chain: the adult diagnosis rests on morphology alone, and the larval barcode cannot be anchored to that adult voucher. The statement that DNA data connect adult and immature stages is not supported when the adult sequence is missing. A practical improvement is to revise the text to separate three claims: adult identification by morphology, larval placement by COI, and the inferred association based on rearing and co-occurrence.

R: We have rewritten the text to clearly separate the reliable results (morphological identification of the adult clerid beetle) from those inferred from our data and our knowledge of the host ants (identity of the immature stages and match with the adult *Phyllobaenus obscurus*).

2- The COI BLAST outcome reported in the Results creates a second identity vulnerability. The closest available sequence is described as only 88.74% identical, paired with a Kimura 2-parameter distance of about 0.116, which is far outside typical species-level barcode expectations for insects and can be large even for many congeneric comparisons. That pattern does not automatically invalidate genus placement, but it does mean that phrases like “placed it without any ambiguity” and “connect adult and immature stages” are overstated relative to the reported similarity metrics. A more defensible interpretation is that the COI fragment supports assignment to Cleridae and is consistent with Phyllobaenus, with species-level confirmation pending a reference sequence for P. obscurus and successful sequencing of the adult voucher.

R: Thank you for your comment. We have re-worded the corresponding parts of the results and discussion, being more cautious and conservative about the inferences that can be drawn from our data.

However, because of the different evolutionary histories of divergent taxa, it is difficult to find a fixed threshold for species/genus delimitation that is suitable to all species/genera. In the case of insects, there is high intraspecific genetic variation. For example, Zhang & Bu (2022) found that approximately one quarter (26.6%) of the species of Insecta (duly identified and with high-quality sequences n = 64,414) in BOLD Systems exhibited high intraspecific genetic variation (> 3%). For Coleoptera, this study found 8,968 species with intraspecific genetic distances greater than 3%.

But you are right, confirmation at the species level of our clerid requires a reference sequence for *P. obscurus*. This is beyond the scope of this study, and in the revised version, we have taken a more cautious approach in both the results and the discussion.

Zhang H, Bu W. 2022. Exploring large-scale patterns of genetic variation in the COI gene among Insecta: Implications for DNA barcoding and threshold-based species delimitation studies. Insects 13, 425). <https://doi.org/10.3390/insects13050425>

3- The Discussion repeats a strong linkage claim that is difficult to justify given the single larval sequence and the lack of adult sequencing, stating that adult and larval stages have been reliably connected through DNA data, morphology, and context. The evidence described supports reliable connection through morphology, rearing, and context; the DNA component supports genus-level placement of one larva, not an adult–larva match. This distinction matters because the novelty depends on accurately attributing the life history to a named species rather than to an unidentified Phyllobaenus species. A clean fix is to revise statements of certainty so that DNA supports genus placement and does not serve as the primary bridge between life stages.

R: Thank you for pointing that out. You are right, we can only say that available evidence (morphology, rearing and co-occurrence) supports the existence of a reliable link between the larva and the adult. However, in accordance with the principle of parsimony, which states that among several competing hypotheses, the simplest (the one requiring the fewest assumptions) is generally the best, it is reasonable to attribute the different life stages observed in our study to the same species of *Phyllobaenus*. We have modified some of our statements accordingly and toned down our assertions.

4- Basic barcode quality control information is not presented, and that omission increases the risk that the COI sequence will be challenged as a numt, low-quality read, or contaminant. The manuscript provides the analytic approach and distance model used for the tree, plus BLAST comparison, but it does not state the amplified fragment length, whether the sequence was translated to check for stop codons, whether chromatograms were inspected for mixed peaks, or whether independent amplification was attempted. The primer choice can be acceptable, but it increases the need to document QC explicitly. Adding a short QC paragraph in Methods and stating chromatogram availability would substantially reduce skepticism.

R: Thank you for pointing this out and for guiding us with your advice throughout the review process. Following this comment, we performed a FastQC analysis, and the graphs for both reads have been attached for your information (see below). The two warning flags arise from the fact that the analysis was performed on a single sequence, whereas this method is primarily designed for datasets containing multiple sequences. This shows that the sequence analyzed in this study unambiguously fits the 40 threshold criteria of nucleotide site quality. The chromatograms were examined for mixed peaks, and any ambiguities were resolved by comparing the forward and reverse sequences. We have also added to the revised text that the COI fragment was sequenced in both directions and that its translation revealed no internal stop codons (see l. 233-240 in the revised version).

5- The phylogenetic presentation is a neighbor-joining tree built from Kimura 2-parameter distances using the ten closest BLAST hits. This construction is acceptable as an illustrative similarity figure, but it is not a strong inferential framework for taxonomic placement when the closest match is distant and sampling is restricted to BLAST neighbors. The absence of support values and broader clerid outgroups means the tree cannot robustly test alternative placements within Cleridae, and it cannot support species-level identity. Recasting the figure as a distance visualization, adding bootstrap support, and supplementing with a likelihood-based analysis with broader Hydnocerinae sampling would strengthen the molecular component without changing the biological story.

R: In the previous version of the manuscript, we presented a neighbor-joining tree constructed using Kimura 2-parameter distances and limited to the ten closest BLAST hits, mainly to illustrate sequence similarity. In fact, the distances are based only on COI variation, and initial analyses performed with MEGA did not produce a topology with bootstrap values supporting the nodes.

Following your suggestion, we expanded the sampling of taxa to include additional Hydnocerinae sequences available in GenBank. The resulting phylogenetic reconstructions are shown below: Figure A shows the neighbor-joining tree, and Figure B shows the maximum likelihood tree, both analyses having been performed with 1,000 bootstrap replications to assess nodal support.

However, the bootstrap values are very low for all nodes, mainly due to the very limited number of Hydnocerinae sequences available and the quality of these sequences (we realigned some of them). Since our aim was not to produce a phylogenetic hypothesis for the subfamily (which would require sequencing several genes), but to illustrate the similarities between sequences, we prefer to keep the previous tree for illustrative purposes. We have explicitly indicated this in the figure caption.

Figure A. Neighbor-joining tree.

Figure B. Maximum likelihood tree.

6- The argument that obligate parasitism or host specificity is unlikely because prevalence is very low and the ant populations are endemic compared to the beetle’s wider distribution is not logically compelled by the data. Rare prevalence is compatible with high specialization in social-insect associates, and broad geographic ranges do not exclude specialized local host use if the parasite uses different hosts across regions or if suitable conditions are patchy. The manuscript also acknowledges context dependence in other parasite systems, which makes a strong inference against obligate parasitism less consistent. A safer approach is to label facultative versus obligate status and host range as open questions, then present multiple plausible explanations for rarity, such as low encounter rate, strong colony defenses, seasonal timing, limited access stage, or sampling detection limits.

R: Thank you for your thorough reading of our text. We have followed your suggestion. In the previous version, we left this question unresoved (see l. 411-414 of the previous version), but you are right, we discussed facultative parasitoidism in depth without addressing obligate parasitoidism. We have reworded certain parts of this paragraph to leave this as open questions needing future research endeavor (see l. 419-431 of the revised version).
